# Kinetics and mechanism of vanadium catalysed asymmetric cyanohydrin synthesis in propylene carbonate

**DOI:** 10.3762/bjoc.6.119

**Published:** 2010-11-03

**Authors:** Michael North, Marta Omedes-Pujol

**Affiliations:** 1 School of Chemistry and University Research Centre in Catalysis and Intensified Processing, Bedson Building, University of Newcastle, Newcastle upon Tyne, UK, NE1 7RU

**Keywords:** cyanohydrin, Hammett, kinetics, propylene carbonate, vanadium

## Abstract

Propylene carbonate can be used as a green solvent for the asymmetric synthesis of cyanohydrin trimethylsilyl ethers from aldehydes and trimethylsilyl cyanide catalysed by VO(salen)NCS, though reactions are slower in this solvent than the corresponding reactions carried out in dichloromethane. A mechanistic study has been undertaken, comparing the catalytic activity of VO(salen)NCS in propylene carbonate and dichloromethane. Reactions in both solvents obey overall second-order kinetics, the rate of reaction being dependent on the concentration of both the aldehyde and trimethylsilyl cyanide. The order with respect to VO(salen)NCS was determined and found to decrease from 1.2 in dichloromethane to 1.0 in propylene carbonate, indicating that in propylene carbonate, VO(salen)NCS is present only as a mononuclear species, whereas in dichloromethane dinuclear species are present which have previously been shown to be responsible for most of the catalytic activity. Evidence from ^51^V NMR spectroscopy suggested that propylene carbonate coordinates to VO(salen)NCS, blocking the free coordination site, thus inhibiting its Lewis acidity and accounting for the reduction in catalytic activity. This explanation was further supported by a Hammett analysis study, which indicated that Lewis base catalysis made a much greater contribution to the overall catalytic activity of VO(salen)NCS in propylene carbonate than in dichloromethane.

## Introduction

**Scheme 1:**
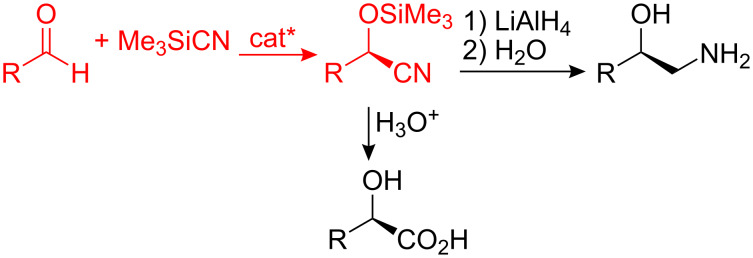
Synthesis and transformation of nonracemic silyl-protected cyanohydrins.

The last 15 years have witnessed an explosion of activity in the area of asymmetric cyanohydrin synthesis [1], mostly using trimethylsilyl cyanide (TMSCN) as the cyanide source to produce enantiomerically enriched silyl-protected cyanohydrins, which can readily be converted into other, pharmaceutically important, bifunctional units, such as α-hydroxy acids and β-amino alcohols [2] (Scheme 1). Asymmetric cyanohydrin synthesis can be achieved by the use of a suitable chiral catalyst, and a wide range of catalysts have been found to catalyse this reaction including enzymes [3–4], organocatalysts [5–6] and metal-based catalysts [1]. All of the most effective catalysts for asymmetric cyanohydrin synthesis have been found to involve cooperative catalysis [7–9], in which the aldehyde is activated by an acidic group and the cyanide source is activated by a basic group. The acid and base catalysts can be present within a single catalyst unit, or can be in separate catalysts, either or both of which may be chiral.

**Figure 1:**
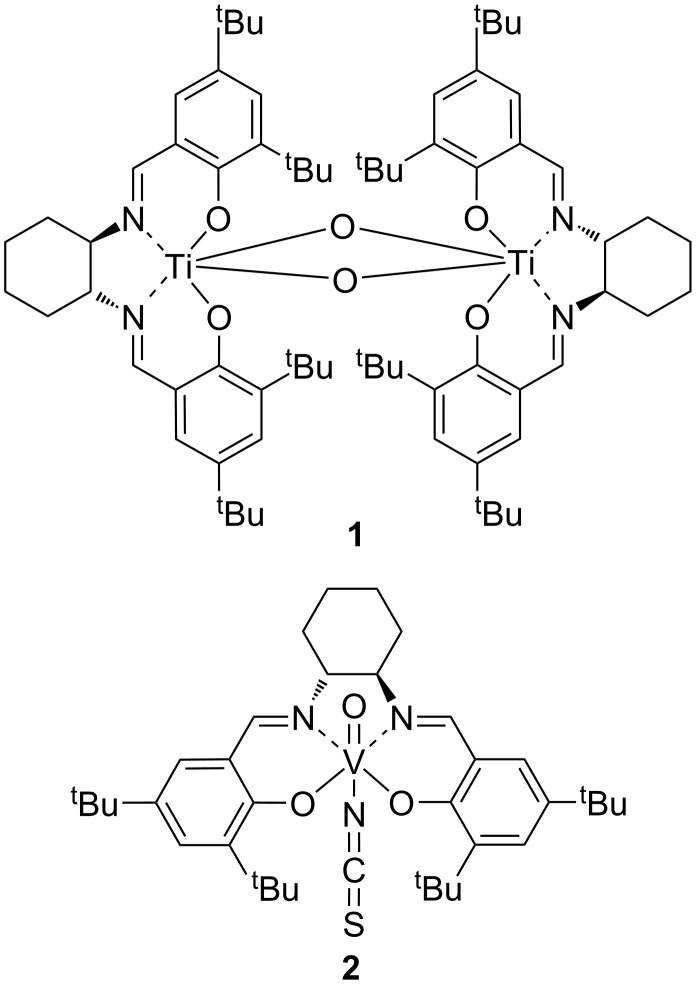
Highly active metal(salen) complexes for asymmetric cyanohydrin synthesis.

Whilst enzymatic catalysts (oxynitrilases) have been extensively developed [3–4] and commercialized [10], they do have the disadvantage of requiring hydrogen cyanide which is toxic and difficult to handle in a laboratory environment, and gives unprotected cyanohydrins which are prone to racemization. Pre-eminent amongst the synthetic catalysts are metal(salen) complexes, especially those based on titanium (**1**) and vanadium (**2**) [1] (Figure 1). Titanium complex **1** will catalyse the asymmetric addition of TMSCN to aromatic aldehydes with 80–90% enantiomeric excess at room temperature with just 0.1 mol % of catalyst [11]. Complex **1** also catalyses the asymmetric addition of other cyanide sources including potassium cyanide [12–15], cyanoformates [15–20] and acyl cyanides [17,19,21] to aldehydes, and will accept some ketones as substrates [22–23]. Recently, a modified version of complex **1**, in which the two salen ligands are covalently linked together has been developed which allows the amount of catalyst used to be reduced to 0.0005 mol % [24–25].

Vanadium based catalysts such as **2** also catalyse the asymmetric addition of TMSCN [26–28] and KCN [13] to aldehydes and are more enantioselective, but less reactive than the titanium based catalyst **1**. Complexes **1** and **2** have been commercialized [10,29–30], immobilized to facilitate their recycling [31–43] and used by other groups as part of synthetic studies [44–46]. Mechanistically, the mode of action of catalyst **1** is well understood [15,20,47–49], involving a bimetallic transition state in which one titanium atom acts as a Lewis acid, coordinating to the aldehyde, and the other forms a titanium–cyanide bond, thus allowing transfer of cyanide to the carbonyl to occur intramolecularly in a highly organized transition state structure. The mode of action of vanadium based catalysts such as **2** is believed to involve two parallel catalytic cycles, the slower of which involves only monometallic species, whilst the other involves bimetallic complexes [28]. Experimental evidence has shown that formation of vanadium(IV) complexes in situ is important [28,50], as is the formation of bimetallic complexes involving vanadium ions in both the +4 and +5 oxidation states [28,51]. Both Lewis acid and Lewis base catalysis are known to be involved in the catalytic cycle, the latter possibly involving the isothiocyanate counterion [52].

**Scheme 2:**
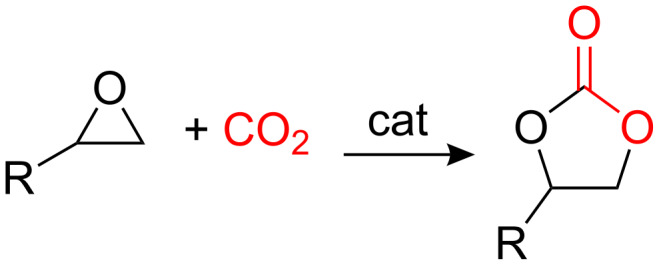
Synthesis of cyclic carbonates.

Despite their many favourable properties, there is one drawback associated with catalysts **1** and **2**; they exhibit highest activity and highest enantioselectivity in chlorinated solvents, optimally dichloromethane. Recently however, we showed that catalyst **2** could be used in propylene carbonate [53]. Propylene carbonate and other cyclic carbonates are starting to attract significant interest as green solvents [54–64], since they can be prepared by a 100% atom economical reaction between epoxides and CO_2_ (Scheme 2) [65]. The green credentials of propylene carbonate are enhanced by the commercialization of a low temperature synthesis of propylene oxide from propene and hydrogen peroxide [66–70], by the development of a greener synthesis of hydrogen peroxide [71], and by the combination of these processes into a one-pot synthesis of propylene oxide from propene, hydrogen and oxygen [72–73]. In addition, it has been shown that in the presence of an appropriate catalyst, the reaction between epoxides and carbon dioxide can be achieved at atmospheric pressure and room temperature [74–77], or in a gas-phase continuous flow reactor [78], thus facilitating the use of waste carbon dioxide in this process [79].

In this paper we give full details of the use of catalyst **2** in propylene carbonate, and describe kinetic studies, which allow differences in the relative importance of Lewis acid and Lewis base catalysis between reactions carried out in dichloromethane and propylene carbonate to be elucidated.

## Results and Discussion

### Synthetic Studies

**Scheme 3:**
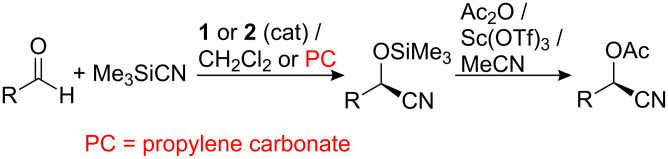
Synthesis of cyanohydrin trimethylsilyl ethers and acetates.

Initially, the compatibility of catalysts **1** and **2** with propylene carbonate was investigated by carrying out the asymmetric addition of TMSCN to a range of aldehydes in both dichloromethane and propylene carbonate under identical reaction conditions. These reactions were all carried out at room temperature for two hours with 0.1 mol % of catalyst, 1.1 equiv of TMSCN and a substrate concentration of 0.56 M. In each case, the enantiomeric excess of the cyanohydrin product was determined by chiral GC after conversion of the trimethylsilyl ether into the corresponding acetate by the method of Kagan [80], a process which is known to cause no racemization (Scheme 3). The results of this study are presented in Table 1.

**Table 1:** Influence of solvent on cyanohydrin synthesis using catalysts **1** and **2**.

Aldehyde	Solvent^a^	Catalyst **1**		Catalyst **2**	
		Conversion^b^	ee^c^	Conversion^b^	ee^c^

PhCHO	CH_2_Cl_2_	95	78	100	86
PhCHO	PC	33	40	73	80
4-FC_6_H_4_CHO	CH_2_Cl_2_	40	76	81	91
4-FC_6_H_4_CHO	PC	24	35	67	76
4-ClC_6_H_4_CHO	CH_2_Cl_2_	98	83	90	93
4-ClC_6_H_4_CHO	PC	20	25	73	76
3-ClC_6_H_4_CHO	CH_2_Cl_2_	83	84	83	89
3-ClC_6_H_4_CHO	PC	53	46	56	62
2-MeC_6_H_4_CHO	CH_2_Cl_2_	76	89	81	96
2-MeC_6_H_4_CHO	PC	47	36	78	73
3-MeC_6_H_4_CHO	CH_2_Cl_2_	95	97	100	99
3-MeC_6_H_4_CHO	PC	30	57	67	93
4-MeC_6_H_4_CHO	CH_2_Cl_2_	82	68	86	87
4-MeC_6_H_4_CHO	PC	16	49	56	86
Me(CH_2_)_7_CHO	CH_2_Cl_2_	71	73	88	83
Me(CH_2_)_7_CHO	PC	98	45	96	67
Me_3_CCHO	CH_2_Cl_2_	93	47	100	86
Me_3_CCHO	PC	100	10	99	76
CyCHO	CH_2_Cl_2_	100	66	100	88
CyCHO	PC	97	19	97	67

^a^PC = propylene carbonate; ^b^Conversions were determined by ^1^H NMR spectroscopy; ^c^Enantiomeric excesses were determined by chiral GC analysis of the cyanohydrin acetates (data presented in Supporting Information File 1). The predominant cyanohydrin derivative always had the (*S*)-configuration.

**Scheme 4:**
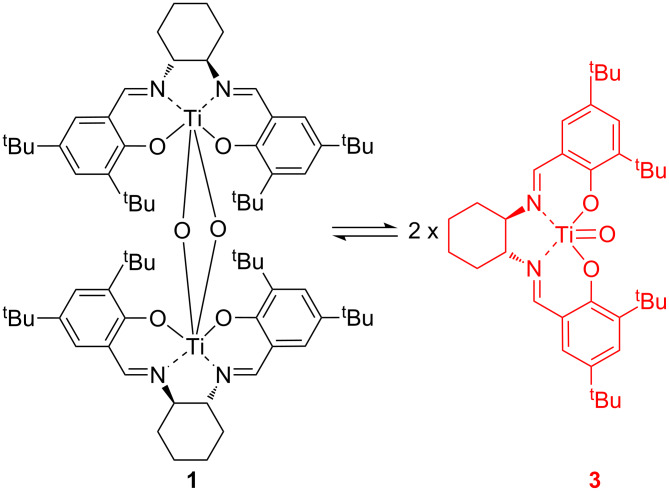
Equilibrium between bimetallic and monometallic Ti(salen) complexes.

It is apparent from Table 1, that for reactions catalysed by titanium based catalyst **1**, changing the solvent to propylene carbonate had a severely detrimental effect on the enantioselectivity of the reactions. In some cases, the enantiomeric excess of the cyanohydrin was more than halved when reactions were carried out in propylene carbonate. For aromatic aldehydes, there was also a substantial reduction in the conversion obtained from reactions carried out in propylene carbonate, though this was not apparent with the aliphatic aldehydes studied. The reason for the lower reactivity and enantioselectivity displayed by catalyst **1** in propylene carbonate can be related to the dissociation of the catalytically active bimetallic complex **1** into the catalytically inactive monometallic complex **3** (Scheme 4). The position of this equilibrium is known to be solvent dependent, with polar solvents favouring the formation of the monometallic species [15]. Propylene carbonate is a polar aprotic solvent with a dielectric constant of 65 [81], and therefore the concentration of catalytically active bimetallic complex **1** will be reduced in this solvent resulting in less effective catalysis.

Reactions catalysed by complex **2** also proceeded more slowly and less enantioselectively in propylene carbonate than in dichloromethane (Table 1). However, the reduction in conversion and enantioselectivity was much less pronounced than for reactions catalysed by complex **1**. Therefore, attempts were made to optimize the reaction conditions for reactions catalysed by complex **2** in propylene carbonate by reducing the reaction temperature to enhance the enantioselectivity and by increasing the reaction time to optimize the conversion. The results of this study are shown in Table 2.

**Table 2:** Optimization of asymmetric cyanohydrin synthesis catalysed by complex **2** in propylene carbonate.

Entry	Aldehyde	*T* (°C)	Time (h)	**2** (mol %)	Conversion^a^	ee^b^

1	PhCHO	rt	4	0.1	83	83
2	PhCHO	rt	24	0.1	92	80
3	PhCHO	rt	2	0.2	86	85
4	PhCHO	0	18	0.1	73	86
5	4-FC_6_H_4_CHO	0	24	0.1	88	88
6	3-ClC_6_H_4_CHO	0	24	0.1	89	82
7	4-ClC_6_H_4_CHO	0	24	0.1	86	80
8	4-MeC_6_H_4_CHO	0	18	0.1	63	90
9	2-MeC_6_H_4_CHO	rt	24	0.1	100	81
10	3-MeC_6_H_4_CHO	rt	24	0.1	93	89
11	4-MeC_6_H_4_CHO	rt	24	0.1	90	83
12	Me(CH_2_)_7_CHO	0	18	0.1	100	61
13	CyCHO	0	18	0.1	92	76
14	Me_3_CCHO	0	18	0.1	100	80
15	Me(CH_2_)_7_CHO	−20	24	0.1	98	75
16	CyCHO	−20	24	0.1	88	77
17	Me_3_CCHO	−20	24	0.1	100	80

^a^Conversions were determined by ^1^H NMR spectroscopy; ^b^Enantiomeric excesses were determined by chiral GC analysis of the cyanohydrin acetates (data presented in Supporting Information File 1). The predominant cyanohydrin derivative always had the (*S*)-configuration.

Reactions carried out with benzaldehyde at room temperature (entries 1 and 2) showed that increasing the reaction time increased the conversion without lowering the enantioselectivity. The conversion could also be increased by doubling the catalyst concentration (entry 3), though this did not enhance the enantioselectivity. Reducing the reaction temperature to 0 °C (entry 4) reduced the rate of reaction so that a reaction time of 18 hours was required to achieve the same conversion as could be achieved in two hours at room temperature (Table 1), but the lower temperature did restore the enantioselectivity to that observed in dichloromethane at room temperature. Electron-deficient aromatic aldehydes also gave good results at 0 °C (entries 5–7), as did 4-methylbenzaldehyde (entry 8), although in this case, whilst the enantioselectivity was higher than that obtained at room temperature in dichloromethane, the conversion was not as high. Therefore, to ensure good conversions, the optimal conditions for electron-rich aromatic aldehydes were taken as room temperature for 24 hours (entries 9–11). The three aliphatic aldehydes studied (entries 12–14) all gave high conversions at 0 °C, but the enantioselectivities were not as high as those obtained at room temperature in dichloromethane. Therefore, for these substrates, the reaction temperature was further reduced to −20 °C (entries 15–17), but this resulted in only a modest improvement in the enantioselectivity, except when nonanal was used as the substrate.

Propylene carbonate has a boiling point of 242 °C and could not be separated from the cyanohydrin trimethylsilyl ethers by distillation. Since the cyanohydrin ethers are liquids and are unstable during chromatography, it was impossible to purify the cyanohydrin trimethylsilyl ethers produced in propylene carbonate. However, one of the main applications of nonracemic cyanohydrins is in the synthesis of α-hydroxy acids [2,29–30], and (*S*)-mandelic acid could be obtained in 60% isolated yield simply by refluxing the mixture of propylene carbonate and mandelonitrile trimethylsilyl ether (81% ee) with 12 N hydrochloric acid for six hours followed by crystallization from ether/hexane. That no racemization occurred during this process was demonstrated by conversion of the mandelic acid into methyl mandelate followed analysis by chiral HPLC (data presented in Supporting Information File 1), which gave an enantiomeric excess of 81%.

### Kinetic and NMR Studies using benzaldehyde

Previous work [28] has shown that the asymmetric addition of TMSCN to benzaldehyde in dichloromethane catalysed by complex **2** follows overall second-order kinetics, the reaction being first order in both benzaldehyde and TMSCN. The rate equation is then represented by Equation 1:

[1]
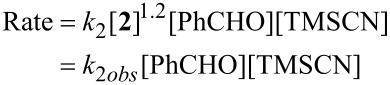


The order with respect to the catalyst (1.2) in Equation 1 provides information on the relative importance of mononuclear and binuclear species in the catalytic cycle [28,47]. A value greater than one implies that the catalyst exists in solution as a mixture of mononuclear and binuclear species, but that the binuclear species is predominantly responsible for the catalysis. The ability of catalyst **2** to function in propylene carbonate provided the opportunity to extend this study to a second solvent system with very different polarity to dichloromethane, and thus offered the potential to obtain a better understanding of the factors that are important for high catalyst activity.

**Figure 2:**
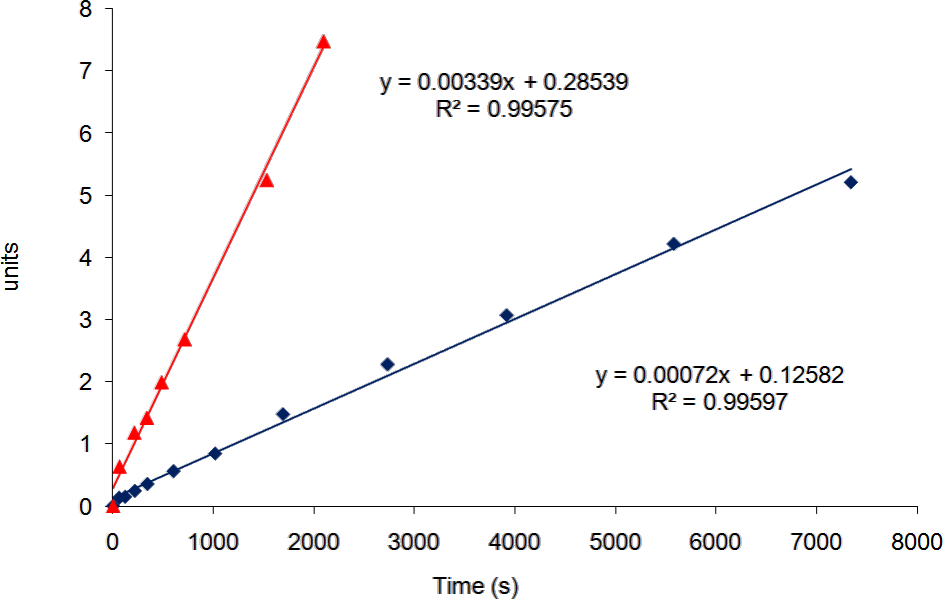
Second-order kinetics plot for the addition of TMSCN to benzaldehyde at 0 °C catalysed by complex **2** in dichloromethane (red) and propylene carbonate (blue). The units for the vertical scale are ln[(B_0_A*_t_*)/(B*_t_*A_0_)]/(A_0_–B_0_), where A = [PhCHO], B = [Me_3_SiCN], and the subscripts 0 and t refer to initial concentrations and concentrations at time *t*, respectively.

Initially, the kinetics of reactions carried out in dichloromethane and propylene carbonate were compared. These reactions were carried out at 0 °C with 0.2 mol % of catalyst **2**, and initial concentrations of benzaldehyde and TMSCN of 0.49 M and 0.52 M, respectively. Reactions were monitored over a period of two hours by removing samples at regular intervals and monitoring the absorbance of residual benzaldehyde at 240–260 nm as previously described [28]. The reaction carried out in propylene carbonate was found neither to follow zero- nor first-order kinetics, but gave an excellent fit to second-order kinetics as shown in Figure 2, which shows the kinetic data obtained in both solvents for comparison. It is apparent from Figure 2 that the reactions in dichloromethane and propylene carbonate obey the same rate equation, but the reaction in propylene carbonate has an observed second-order rate constant a factor of four smaller than the reaction in dichloromethane, consistent with the lower conversions observed for reactions carried out in propylene carbonate (Table 1).

**Figure 3:**
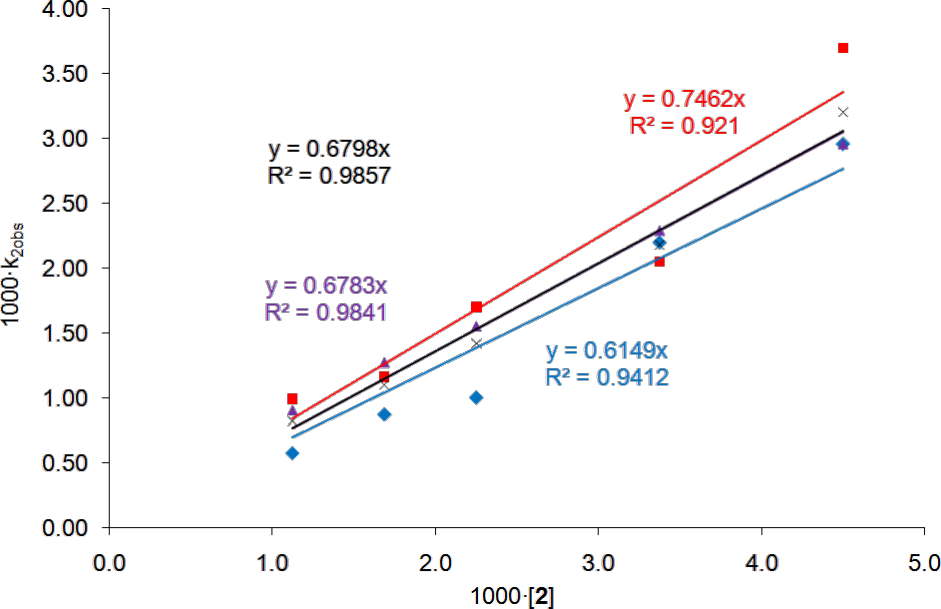
Plot of *k*_2obs_ against [2], showing that the reactions are first order with respect to the concentration of catalyst **2** (data presented in Supporting Information File 1). The red, blue and purple data and best-fit lines correspond to the three individual data sets given in Table 3. The black data and best-fit line correspond to the average data.

To determine the order with respect to catalyst **2** and hence to investigate if changing the solvent from dichloromethane to propylene carbonate affected the aggregation state of the catalyst, reactions were carried out in propylene carbonate at 0 °C with five different concentrations of catalyst **2** (Table 3). The kinetics at each catalyst concentration were determined in triplicate, using two different batches of propylene carbonate, and the average value of the rate constant was calculated from all three data points for each concentration. As shown in Figure 3, plots of *k*_2obs_ against the concentration of catalyst **2** could be fitted to a straight line, showing that in propylene carbonate the reactions are first order with respect to the concentration of the catalyst (since *k*_2obs_ = *k*_2_[**2**]^x^ where x is the order with respect to the catalyst). This was further supported by a plot of log(*k*_2obs_) against log([2]), which had a slope of 0.997 (data presented in Supporting Information File 1). Thus, the order with respect to the catalyst decreases from 1.2 in dichloromethane [28] to 1.0 in propylene carbonate. This implies that in propylene carbonate, the catalyst exists only as mononuclear species and that these are exclusively responsible for the catalysis. Since it is known that catalysis by binuclear complexes formed from catalyst **2** is faster than catalysis by mononuclear complexes [28], this is therefore consistent with the reduction in reaction rate when the solvent is changed from dichloromethane to propylene carbonate. The reason for the lack of formation of bimetallic complexes in propylene carbonate is probably due to the polarity of the solvent (dielectric constant 65 [81]), which will stabilise the highly polar V=O bonds present in the mononuclear species.

**Table 3:** Second-order rate constants at 0 °C for the addition of TMSCN to benzaldehyde obtained at different concentrations of complex **2**.^a^

Entry	[2] (mol %)	*k*_2obs1_ (M^−1^s^−1^)	*k*_2obs2_ (M^−1^s^−1^)	*k*_2obs3_ (M^−1^s^−1^)	*k*_2obs avg_ (M^−1^s^−1^)

1	1.13 mM (0.2)	0.00057	0.00099	0.00090	0.00083 ± 0.00016
2	1.69 mM (0.3)	0.00087	0.00116	0.00127	0.00110 ± 0.00023
3	2.25 mM (0.4)	0.00100	0.00170	0.00155	0.00142 ± 0.00042
4	3.38 mM (0.6)	0.00220	0.00205	0.00229	0.00218 ± 0.00013
5	4.50 mM (0.8)	0.00296	0.00370	0.00296	0.00321 ± 0.00049

^a^*k*_2obs1–3_ refer to the results of three separate experiments at the specified catalyst concentration (data presented in Supporting Information File 1). *k*_2obs avg_ is the average value of the three separate measurements.

**Figure 4:**
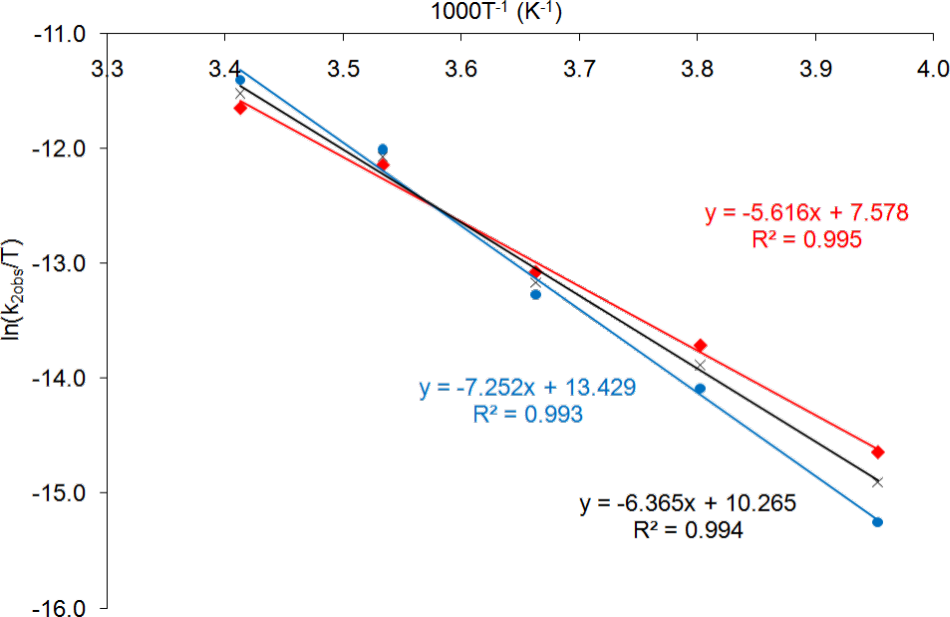
Eyring plot to determine the activation parameters for catalyst **2** in propylene carbonate. The red and blue data and best-fit lines correspond to the two individual data sets given in Table 4. The black data and best-fit line correspond to the average data.

Having determined the order with respect to catalyst **2** in propylene carbonate, a variable temperature kinetics study was carried out to determine the activation parameters in propylene carbonate and to allow these to be compared with those previously reported for the use of catalyst **2** in dichloromethane [28]. Thus, reactions were carried out at five temperatures between 253 and 293 K. The resulting rate data are presented in Table 4. The corresponding Eyring plot is shown in Figure 4.

**Table 4:** Second-order rate constants at 253 K to 293 K for the addition of TMSCN to benzaldehyde.^a^

Temperature (K)	*k*_2obs1_ (M^−1^s^−1^)	*k*_2obs2_ (M^−1^s^−1^)	*k*_2obs avg_ (M^−1^s^−1^)

253	0.00011	0.00006	0.00009 ± 0.00003
263	0.00029	0.00020	0.00025 ± 0.00005
273	0.00057	0.00047	0.00052 ± 0.00005
283	0.00150	0.00172	0.00161 ± 0.00011
293	0.00255	0.00325	0.00290 ± 0.00035

^a^[PhCHO]_0_ = 0.49 M, [Me_3_SiCN]_0_ = 0.49 and [2] = 0.98 mM (data presented in Supporting Information File 1).

The Eyring equation (Equation 2) relates the rate constant for a reaction to the enthalpy and entropy of activation. Replacing the actual rate constant in Equation 2 with *k*_2obs_ (*k*_2obs_ = *k*_2_[**2**]^x^) and rearranging gives Equation 3, which, after taking the logarithm of both sides, gives Equation 4. The enthalpy of activation (Δ*H*^‡^) can then be obtained from the slope of the best-fit line from the data plotted in Figure 4 and was found to be 67.8 (±0.2) kJ mol^−1^. The entropy of activation (Δ*S*^‡^) can be obtained from the y-axis intercept, once the contributions of the fundamental constants and x·ln[2] are subtracted. The latter was only possible as the reaction order with respect to catalyst **2** (x) had been determined to be 1.0 as discussed above. This gave a value for Δ*S*^‡^ of −54 (±26) J mol^−1^ K^−1^. The values for Δ*H*^‡^ and Δ*S*^‡^ are very different to those previously determined for the asymmetric addition of trimethylsilyl cyanide to benzaldehyde catalysed by complex **2** in dichloromethane [28] (Δ*H*^‡^ = 20.4 kJ mol^−1^ and Δ*S*^‡^ = −136 J mol^−1^ K^−1^), though the corresponding Gibbs free energies of activation (Δ*G*^‡^) at 273 K are similar at 53.1 and 57.5 kJ mol^−1^ for reactions carried out in propylene carbonate and dichloromethane, respectively.

[2]
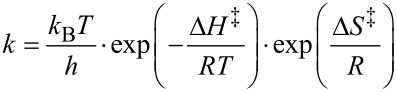


[3]
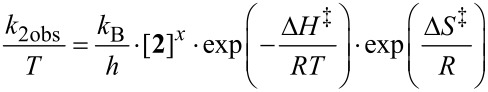


[4]
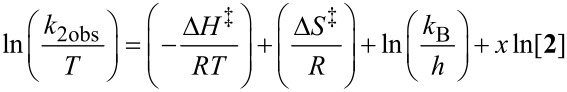


(*k*_B_ = Boltzmann’s constant, *h* = Planck’s constant, *R* = gas constant)

Since differences in the Gibbs free energy of activation could not account for the reduction in rate constant between reactions in dichloromethane and propylene carbonate, the most likely explanation for the observed rate reduction is due to differences in the efficiency with which complex **2** is converted into species which are involved in the catalytic cycle. If propylene carbonate inhibits the conversion of complex **2** into catalytically competent species, then there will be a lower concentration of catalytically active species present in propylene carbonate than in dichloromethane, thus resulting in the observed lower rate of reaction.

**Figure 5:**
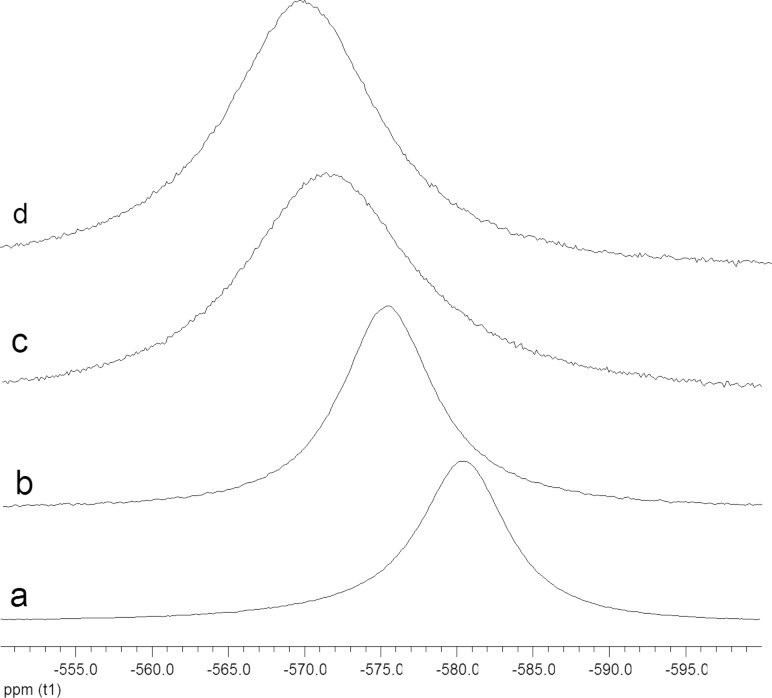
^51^V NMR spectra of complex **2** recorded at 50 °C. a) Spectrum in CDCl_3_; b) spectrum in CDCl_3_ with 500 equiv of PhCHO added; c) spectrum in propylene carbonate; d) spectrum in propylene carbonate with 500 equiv of PhCHO added. All spectra were recorded with a complex **2** concentration of 24 mM and for spectra b and d, the concentration of benzaldehyde was 4.8 M.

**Figure 6:**
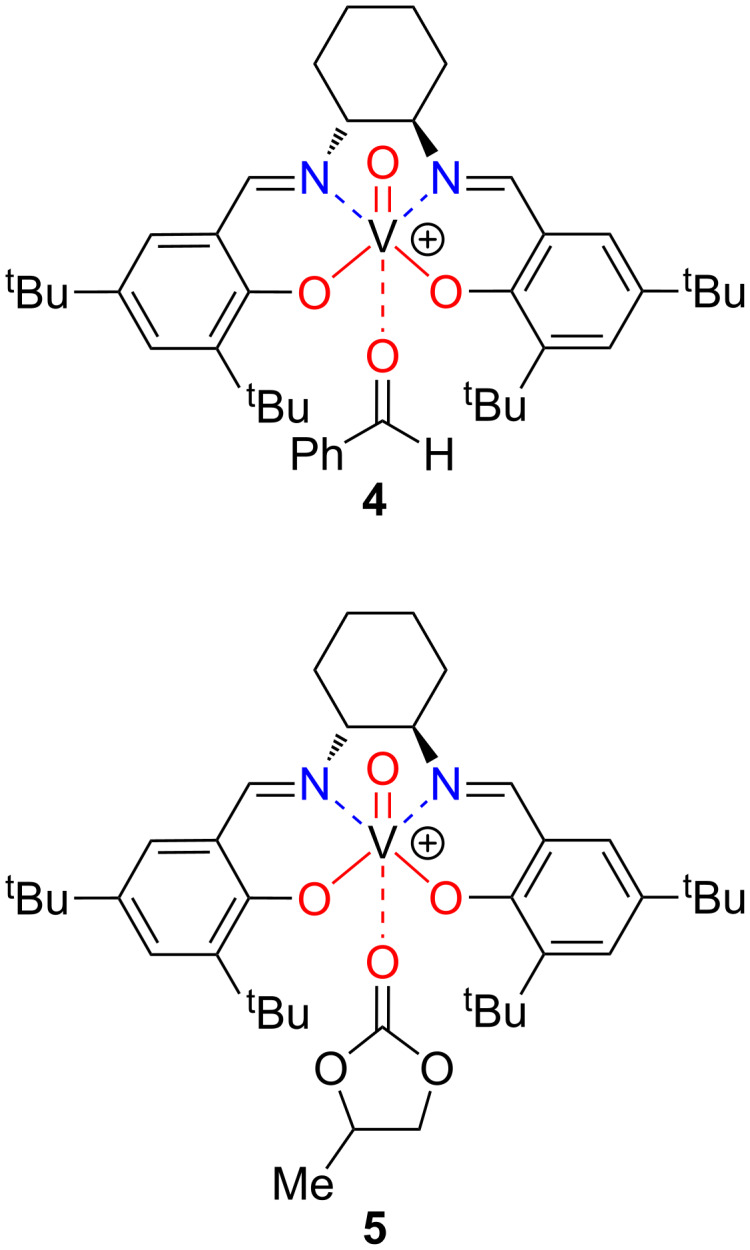
Structures consistent with the ^51^V NMR spectra.

Evidence to support this hypothesis came from ^51^V NMR studies of complex **2** (Figure 5). The spectrum of complex **2** in CDCl_3_ shows a resonance at −580 ppm (Figure 5a). It is known from X-ray crystallography that the isothiocyanate unit in complex **2** is directly bound to the vanadium ion through the nitrogen atom. Thus, the vanadium ion is six-coordinate, and is bound to three oxygen atoms and three nitrogen atoms. Addition of benzaldehyde (500 equiv) to this solution results in a change in the chemical shift of the vanadium ion to −575 ppm (Figure 5b), consistent with the formation of a complex **2**/benzaldehyde (see complex **4** in Figure 6), in which the vanadium ion is bound to four oxygen atoms and two nitrogen atoms. When the ^51^V NMR spectrum of complex **2** is recorded in propylene carbonate, the ^51^V NMR signal is observed at −571 ppm (Figure 5c), again indicative of formation of a species, such as **5**, in which the vanadium ion is bound to four oxygen atoms and two nitrogen atoms. The competitive formation of structure **5** would reduce the amount of complex **4** present in solution, thus reducing the concentration of catalytically competent species and hence reducing the rate of asymmetric cyanohydrin synthesis in propylene carbonate compared to dichloromethane. Addition of benzaldehyde (500 equiv) to the propylene carbonate spectrum resulted in only a small additional change in the chemical shift to −569 ppm (Figure 5d).

The half-widths of the signals recorded in propylene carbonate (1730 Hz and 1510 Hz for Figure 5c and Figure 5d, respectively) are much greater than the corresponding half-widths of the signals recorded in dichloromethane (930 Hz and 950 Hz for Figure 5a and Figure 5b, respectively). This is also indicative of exchange processes involving species **2**, **4** and **5** occurring in propylene carbonate.

The enthalpy of activation for the asymmetric addition of trimethylsilyl cyanide to benzaldehyde was found to be much higher in propylene carbonate than in dichloromethane, which is consistent with only one of the two reaction components (benzaldehyde or TMSCN) being activated by the mononuclear, catalytically active species present in propylene carbonate, whilst both reaction components are activated and pre-organized for reaction by the binuclear, catalytically active species present in dichloromethane [28]. The less negative value for the entropy of activation in propylene carbonate compared to that determined in dichloromethane is also consistent with a less tightly organized transition state, again consistent with only one of the reaction components interacting with the catalyst. To investigate this further, a Hammett analysis was undertaken using a range of substituted benzaldehydes.

### Hammett analysis

It is well established that the asymmetric addition of TMSCN to aldehydes can be catalysed by both Lewis acids and Lewis bases [1]. A Lewis acid catalyst activates the aldehyde by formation of an aldehyde-Lewis acid complex (e.g., **4**) whilst a Lewis base catalyst activates the TMSCN through formation of a hypervalent silicon species [82] or the formation of cyanide anions. The most effective catalysts possess both Lewis acidity and Lewis basicity and so can simultaneously activate both the aldehyde and TMSCN [1].

**Figure 7:**
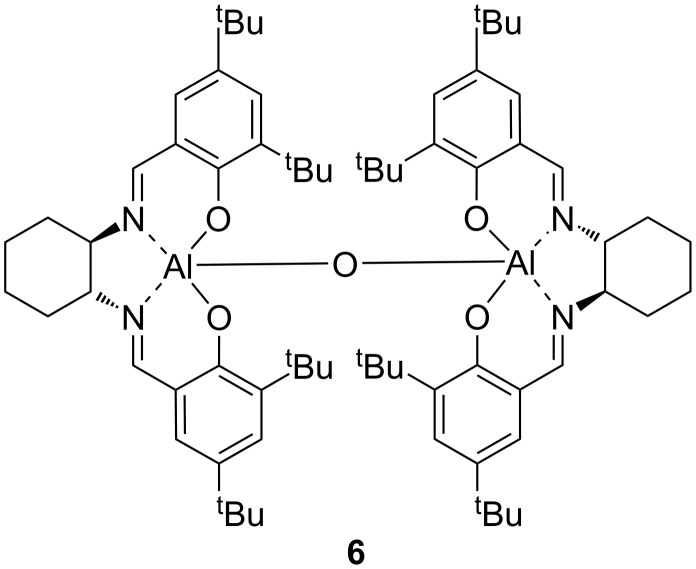
Bimetallic aluminium(salen) complex for asymmetric cyanohydrin synthesis.

**Figure 8:**
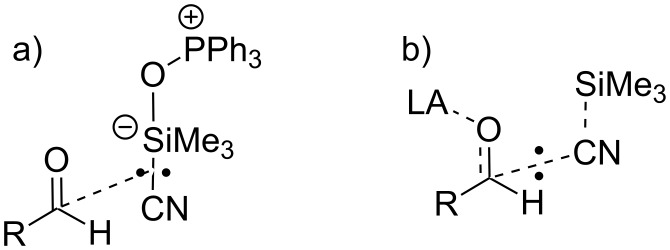
Rate determining transition states for asymmetric cyanohydrin synthesis: a) when Lewis base catalysis is dominant; and b) when Lewis acid catalysis is dominant.

**Figure 9:**
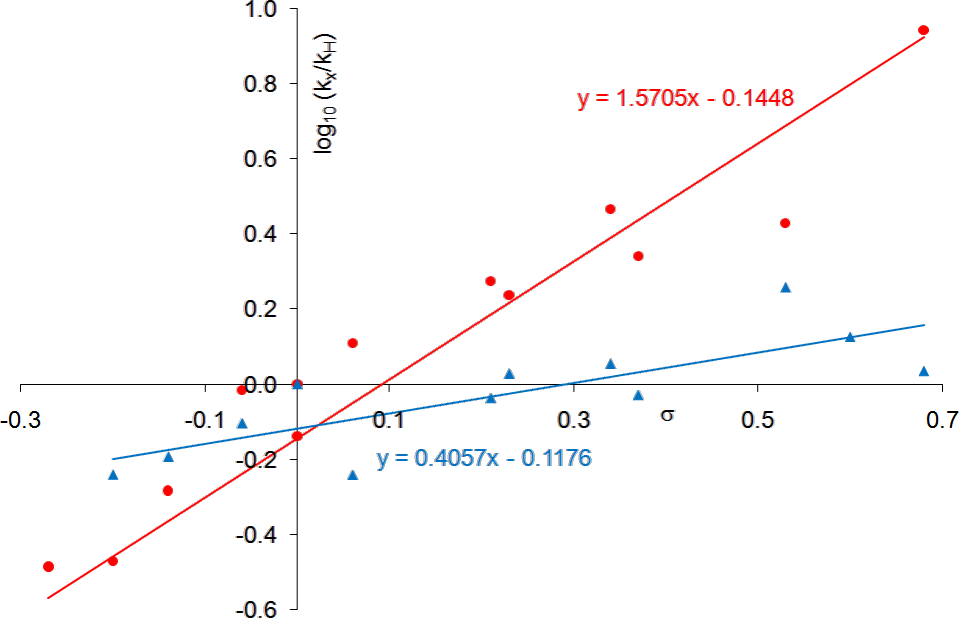
Hammett correlations with catalyst **2** at 0 °C. Data in red are obtained in dichloromethane [52], whilst data in blue are obtained in propylene carbonate.

We have recently shown [52] that a Hammett analysis correlating the rate of reaction of *meta*- and *para*-substituted benzaldehydes with their substituent constants can be used to investigate the relative importance of Lewis acid and Lewis base catalysis in a catalysed reaction. This methodology was developed using the asymmetric addition of TMSCN to aldehydes catalysed by complexes including **1, 2** and **6** (Figure 7) in dichloromethane. A reaction which is predominantly Lewis base catalysed would be expected to produce a Hammett plot with a reaction constant (ρ) close to zero, since the aldehyde is not activated in the catalytic step; so during the rate determining transition state, the negative charge will largely be located on silicon as shown in Figure 8a. This was found to be the case (ρ = +0.4) for asymmetric cyanohydrin synthesis catalysed by bimetallic aluminium(salen) complex **6** in the presence of triphenylphosphine oxide [83–84], indicating that most of the catalysis in this case was due to activation of the TMSCN by the triphenylphosphine oxide rather than activation of the aldehyde by the metal(salen) complex. In contrast, reactions catalysed by complex **1** gave a Hammett plot with a reaction constant of +2.4, indicating that there was a significant increase in negative charge at the benzylic position of the aldehyde during the transition state, and hence that complex **1** functioned predominantly as a Lewis acid catalyst, activating the aldehyde towards attack by cyanide, and resulting in more charge transfer to the benzylic position of the aldehyde during the transition state for formation of the new carbon-carbon bond as shown in Figure 8b. Asymmetric cyanohydrin synthesis catalysed by complex **2** in dichloromethane was found to give a Hammett plot with an intermediate reaction constant of +1.6, indicating that both Lewis acid and Lewis base catalysis were operative in this case. Since the kinetic and NMR data suggested that changing the solvent to propylene carbonate was inhibiting the Lewis acidity of complex **2**, this should be reflected in a reduction in the reaction constant of a Hammett analysis. Therefore, the kinetics of the asymmetric addition of TMSCN to 12 *meta*- and *para*-substituted benzaldehydes were determined (Table 5) and used to construct a Hammett plot (Figure 9).

**Table 5:** Rate constants used to construct the Hammett plot.^a^

Entry	Aldehyde	*k*_(a)_ (M^−1^s^−1^)	*k*_(b)_ (M^−1^s^−1^)	*k*_(avg)_ (M^−1^s^−1^)	ee^b^

1	PhCHO	0.00090	0.00090	0.00090	85
2	3,4-Cl_2_C_6_H_3_CHO	0.00117	0.00124	0.00120 ± 0.00004	40^c^
3	4-ClC_6_H_4_CHO	0.00093	0.00099	0.00096 ± 0.00003	74
4	4-MeC_6_H_4_CHO	0.00058	0.00058	0.00058	77
5	4-FC_6_H_4_CHO	0.00056	0.00047	0.00052 ± 0.00005	84
6	3-FC_6_H_4_CHO	0.00104	0.00100	0.00102 ± 0.00002	72
7	3-MeC_6_H_4_CHO	0.00068	0.00070	0.00069 ± 0.00001	90
8	4-F_3_CC_6_H_4_CHO	0.00153	0.00173	0.00163 ± 0.00010	44
9	4-BrC_6_H_4_CHO	0.00089	0.00076	0.00083 ± 0.00007	70
10	3,5-F_2_C_6_H_3_CHO	0.00084	0.00110	0.00097 ± 0.00013	45
11	3,4-Me_2_C_6_H_3_CHO	0.00055	0.00048	0.00052 ± 0.00004	85^c^
12	3-ClC_6_H_4_CHO	0.00078	0.00090	0.00084 ± 0.00006	57

^a^All reactions were carried out in duplicate (to give k_(a)_ and k_(b)_, respectively) in propylene carbonate at 0 °C with [aldehyde]_0_ = 0.5 M, [Me_3_SiCN]_0_ = 0.55 M and [2] = 1.0 mM. ^b^Enantiomeric excesses were determined by chiral GC analysis of the cyanohydrin acetates (data presented in Supporting Information File 1) unless stated otherwise. The predominant cyanohydrin derivative always had the (*S*)-configuration. ^c^Determined by ^1^H NMR spectroscopy in the presence of (*R*)-mandelic acid and DMAP.

All of the aldehydes included in Table 5 gave nonracemic cyanohydrin trimethylsilyl ethers, confirming that in each case the reaction was catalysed by complex **2**. In most cases the enantiomeric excess of the cyanohydrin trimethylsilyl ether was determined by chiral GC analysis after conversion to the corresponding cyanohydrin acetate [80] as discussed above. However, the cyanohydrin acetates derived from 3,4-dichlorobenzaldehyde and 3,4-dimethylbenzaldehyde were not separated by chiral GC, therefore the enantiomeric excess was determined by ^1^H NMR analysis of the free cyanohydrin obtained by hydrolysis of the acetate [85], in the presence of (*R*)-mandelic acid and DMAP [86].

It is apparent from Figure 9 that the reaction constant for cyanohydrin synthesis catalysed by complex **2** does indeed decrease significantly (from +1.6 to +0.4) when the solvent is changed from dichloromethane to propylene carbonate. The results obtained in propylene carbonate are almost identical to those previously obtained with complex **6** and triphenylphosphine oxide as catalyst [52], and are entirely consistent with a significant reduction in the Lewis acidity of complex **2** in propylene carbonate compared to dichloromethane. This is manifested as an increase in the relative importance of Lewis base catalysis and hence a decrease in the reaction constant. It is important to note however, that complex **2** must still possess some Lewis acidity in propylene carbonate, otherwise cyanohydrin synthesis would not occur in the chiral environment around the vanadium ion and racemic cyanohydrin trimethylsilyl ethers would be obtained.

## Conclusion

Asymmetric cyanohydrin synthesis catalysed by VO(salen)NCS complex **2** can be carried out in propylene carbonate, thus providing a green, alternative solvent to dichloromethane. Reactions in propylene carbonate are slower and less enantioselective than those carried out in dichloromethane; though by optimization of the reaction conditions, high enantioselectivities can still be obtained. A study of the reaction kinetics showed that complex **2** is active only as mononuclear species in propylene carbonate. Kinetic and NMR studies also showed that the propylene carbonate can coordinate to the vanadium ion of complex **2**, thus reducing its Lewis acidity and accounting for the decrease in reaction rate observed in propylene carbonate. The lower Lewis acidity of complex **2** in propylene carbonate was confirmed by a Hammett analysis using substituted benzaldehydes, which gave a reaction constant of only 0.4 in propylene carbonate compared to 1.6 in dichloromethane.

The lower enantioselectivities observed in propylene carbonate under identical reaction conditions to those used in dichloromethane can be explained in two ways. It is possible that in propylene carbonate, some addition of TMSCN to aldehydes occurs exclusively by Lewis base catalysis (using the thiocyanate anion as the Lewis base) and hence is independent of the chiral VO(salen) unit, thus forming some racemic cyanohydrin trimethylsilyl ether. Alternatively, all of the catalysis may occur within the coordination sphere of the VO(salen) unit by cooperative Lewis acid/Lewis base catalysis, but the aldehyde may be less tightly bound to the vanadium ion in the more polar propylene carbonate than in dichloromethane. This would result in the aldehyde being further from the chiral salen ligand during the key transition state and hence less effective transfer of chirality from the ligand to the newly formed stereocentre.

## Experimental

### 

#### General procedure for the synthesis and analysis of cyanohydrin trimethylsilyl ethers in propylene carbonate

The aldehyde (0.98 mmol) was added to a solution of catalyst **1** or **2** (0.98 μmol, 0.1 mol %) in propylene carbonate (1.75 mL) at the specified temperature. Me_3_SiCN (1.12 mmol, 0.15 mL) was then added and the reaction mixture stirred for the specified time. The solution was then passed through a short silica plug eluting with CH_2_Cl_2_. The eluent was evaporated in vacuo to remove the CH_2_Cl_2_, and the residue analysed by ^1^H NMR spectroscopy to determine the conversion. To determine the enantiomeric excess, Ac_2_O (2.0 mmol, 0.15 mL) and Sc(OTf)_3_ (5 mg, 0.01 mmol) were added to the stirred residue. After 20 min, the reaction mixture was passed through a short silica plug eluting with MeCN. The resulting solution was analysed by chiral GC using a Supelco Gamma DEX 120 fused silica capillary column (30 m x 0.25 mm) with hydrogen as a carrier gas. Details of the analysis of each cyanohydrin acetate are given in the Supporting Information File 1. When no separation could be achieved by chiral GC, the cyanohydrin acetate (0.985 mmol) was dissolved in ethanol (3 mL), *p*-TsOH·H_2_O (187 mg, 0.985 mmol) was added, and the mixture stirred at room temperature for 2 days. The solvent was evaporated in vacuo and the residue purified by column chromatography eluting with a gradient from 1:15 EtOAc/hexane to 1:6 EtOAc/hexane to give the deprotected cyanohydrin. (*R*)-Mandelic acid (2.74 mg, 18 μmol), DMAP (1.73 mg, 18 μmol) and CDCl_3_ (0.6 mL) were mixed in an NMR tube. The cyanohydrin (18 μmol) was then added and the solution analysed by ^1^H NMR spectroscopy.

#### (*S*)-Mandelic acid

To a solution of mandelonitrile trimethylsilyl ether in propylene carbonate, obtained following the general procedure above, was added 12 N HCl (10 mL). The mixture was heated at reflux for 6 h, cooled to rt and basified with 10% aqueous NaOH solution. The aqueous solution was extracted with ether (3 x 10 mL), acidified with 12 N HCl and extracted again with ether (3 x 10 mL). The last three ethereal extracts were combined, dried (Na_2_CO_3_) and evaporated in vacuo to give a yellow solid which was recrystallised at 4 °C from ether/hexane and the resulting solid washed with hexane to give mandelic acid (91 mg, 60%) as white crystals. δ_H_ (300 MHz, CDCl_3_) 5.26 (1H, s, CHO), 7.3–7.5 (5H, m, ArH).

#### (*S*)-Methyl mandelate

Mandelic acid (50 mg, 0.33 mmol) was suspended in toluene (10 mL), then methanol (2 mL) was added to give a homogenous solution. One drop of concentrated H_2_SO_4_ was added and the mixture heated at reflux for 4 h. The reaction mixture was then cooled to room temperature, the solvents were evaporated in vacuo and the residue was dissolved in water (10 mL). The aqueous solution was extracted with ether (3 x 5 mL). The ethereal extract was dried over anhydrous Na_2_SO_4_ and concentrated in vacuo to give methyl mandelate (28 mg, 50%) as a pale yellow solid. δ_H_ (300 MHz, CDCl_3_) 3.77 (3H, s, CH_3_), 5.20 (1H, s, CHO), 7.3–7.5 (5H, m, ArH).

#### General procedure for kinetics experiments in propylene carbonate

A solution of catalyst **2** (0.2–0.8 mol %) in freshly distilled propylene carbonate (1.75 mL) was added to a round-bottomed flask fitted with a magnetic stirrer bar and a SubaSeal stopper. The reaction temperature was adjusted by a water bath (water/ice for 0 °С) or cryostat for reactions below 0 °C; temperatures other than 0 °C were kept within a ±0.5 °С range. A 0.5 μL aliquot was taken and diluted with dry CH_2_Cl_2_ (3.5 mL). This solution was used for UV-baseline calibration at 240–260 nm. Freshly distilled aldehyde (0.96 mmol) was then added, and a *t* = 0 aliquot was taken and diluted as described for the baseline calibration sample. Me_3_SiCN (0.15 mL, 1.125 mmol) was added, and aliquots of the reaction were taken and diluted at appropriate intervals for a period of 2 h. After completion of the kinetics analysis, the reaction mixture was passed through a short silica plug eluting with CH_2_Cl_2_. The solvent was evaporated and the residue converted into mandelonitrile acetate as described above to allow the enantiomeric excess of the cyanohydrin to be determined.

## Supporting Information

Chiral GC traces for all chiral cyanohydrin acetates and chiral HPLC data for methyl mandelate. NMR spectra of cyanohydrins in the presence of mandelic acid. Additionally, all of the kinetic data used to determine the catalyst order, the activation parameters and construct the Hammett plot are given.

File 1 Analytical data for all chiral compounds.
