# Supplementary material for: Kinetics and mechanism of vanadium catalysed asymmetric cyanohydrin synthesis in propylene carbonate
Source: Beilstein J Org Chem. 2010 Nov 3;6:1043–55. doi: 10.3762/bjoc.6.119 (PMC2982084; doi:10.3762/bjoc.6.119)

Supporting Information

for

Kinetics and mechanism of vanadium catalysed asymmetric cyanohydrin synthesis in propylene carbonate

Michael North*[1] and Marta Omedes-Pujol[1]

School of Chemistry and University Research Centre in Catalysis and Intensified Processing, Bedson Building, University of Newcastle, Newcastle upon Tyne, UK, NE1 7RU.

Contents

Chiral GC traces

Chiral GC conditions	3
Cyanohydrin acetate derived from benzaldehyde	4
Cyanohydrin acetate derived from 4-fluorobenzaldehyde	8
Cyanohydrin acetate derived from 4-chlorobenzaldehyde	11
Cyanohydrin acetate derived from 3-chlorobenzaldehyde	14
Cyanohydrin acetate derived from 2-methylbenzaldehyde	17
Cyanohydrin acetate derived from 3-methylbenzaldehyde	20
Cyanohydrin acetate derived from 4-methylbenzaldehyde	23
Cyanohydrin acetate derived from nonanal	26
Cyanohydrin acetate derived from pivaldehyde	29
Cyanohydrin acetate derived from cyclohexane carboxaldehyde	32
Cyanohydrin acetate derived from 3-fluorobenzaldehyde	35
Cyanohydrin acetate derived from 4-trifluoromethylbenzaldehyde	36
Cyanohydrin acetate derived from 4-bromobenzaldehyde	37
Cyanohydrin acetate derived from 3,5-difluorobenzaldehyde	38

Chiral HPLC traces

HPLC of methyl mandelate	39

NMR spectra of (*R*)-mandelic acid complexes used to determine enantiomeric excesses

(3,4-Dichlorophenyl)hydroxyacetonitrile	40
(3,4-Dimethylphenyl)hydroxyacetonitrile	41

Kinetic plots used to determine the order with respect to catalyst concentration

0.2 mol % catalyst	42
0.3 mol % catalyst	43
0.4 mol % catalyst	44
0.6 mol % catalyst	45
0.8 mol % catalyst	46
plot of log([**2**]) against log($k_{2obs\ avg}$)	47

Kinetic plots used to determine the activation parameters in propylene carbonate

Kinetics at 253 K	48
Kinetics at 263 K	49
Kinetics at 273 K	50

Kinetics at 283 K 51
Kinetics at 293 K 52

Kinetic plots used to construct the Hammett plot

Benzaldehyde 53
3,4-Dichlorobenzaldehyde 54
4-Chlorobenzaldehyde 55
4-Methylbenzaldehyde 56
4-Fluorobenzaldehyde 57
3-Fluorobenzaldehyde 58
3-Methylbenzaldehyde 59
4-Trifluoromethylbenzaldehyde 60
4-Bromobenzaldehyde 61
3,5-Difluorobenzaldehyde 62
3,4-Dimethylbenzaldehyde 63
3-Chlorobenzaldehyde 64

Chiral GC conditions

Chiral gas chromatography was performed on a Varian450-GC instrument with a TCD detector using Supelco Gamma DEX 120 fused silica capillary columns (26–30 m × 0.25 mm × 0.25 μm film thickness) with hydrogen as a carrier gas. Analyses were performed using one of the following methods:

Method 1: initial temperature 95 °C, hold for 2 minutes then ramp rate of 3 °C/minute to 180 °C, then hold for another 5 minutes. Flow rate: 2 mL/min.

Method 2: initial temperature 95 °C, hold for 2 minutes then ramp rate of 5 °C/minute to 180 °C, then hold for another 5 minutes. Flow rate: 1 mL/min.

Method 3: initial temperature 95 °C, hold for 2 minutes then ramp rate of 2 °C/minute to 180 °C, then hold for another 5 minutes. Flow rate: 2 mL/min.

Method 4: initial temperature 95 °C, hold for 5 minutes then ramp rate of 0.5 °C/minute to 180 °C, then hold for another 5 minutes. Flow rate: 2 mL/min.

Method 5: initial temperature 95 °C, hold for 2 minutes then ramp rate of 5 °C/minute to 180 °C, then hold for another 5 minutes. Flow rate: 2 mL/min.

Retention times were found to vary as the chiral GC column aged and were also different from column to column. In many cases it was necessary to change the method used to analyse a particular cyanohydrin acetate when the chiral GC column was replaced. The GC traces presented in the following pages were recorded over a period of three years on three different chiral columns, which accounts for the variation in retention times.

Cyanohydrin acetate derived from benzaldehyde (Analysed using GC methods 1 or 5).

Racemate (method 1)

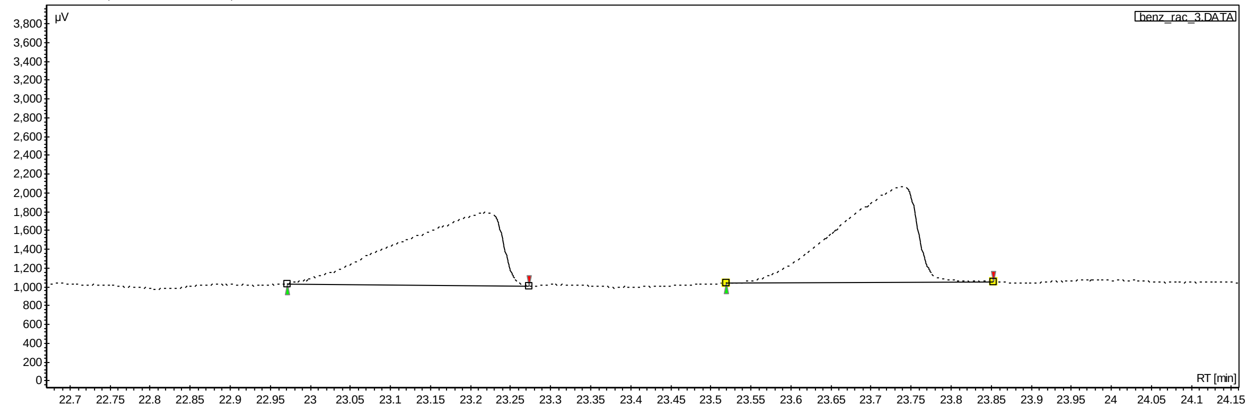

#	Name	Time [Min]	Quantity [% Area]	Height [µV]	Area [µV.Min]	Area % [%]
2	UNKNOWN	23.22	49.25	779.2	113.7	49.252
1	UNKNOWN	23.74	50.75	1021.3	117.1	50.748
Total			100.00	1800.5	230.8	100.000

Racemate (method 5)

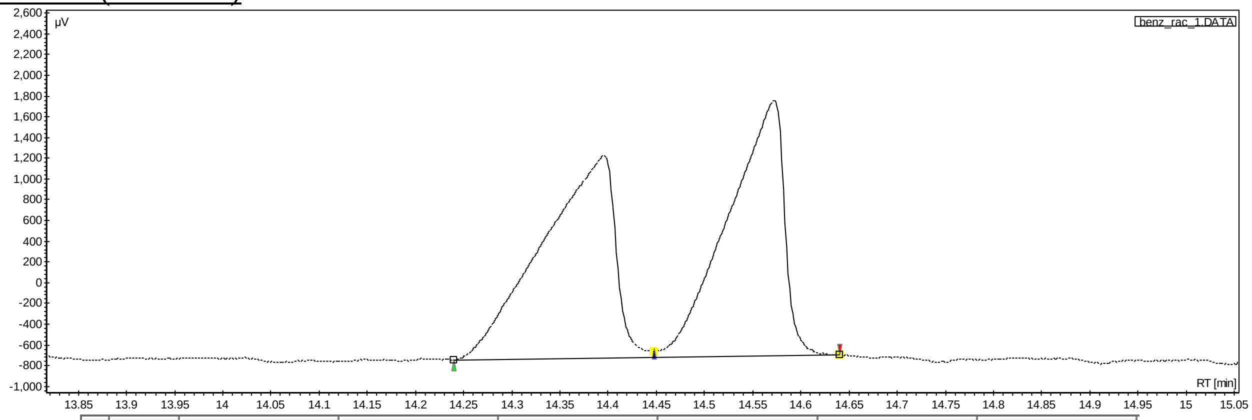

#	Name	Time [Min]	Quantity [% Area]	Height [µV]	Area [µV.Min]	Area % [%]
1	UNKNOWN	14.40	50.99	1949.6	173.4	50.985
2	UNKNOWN	14.57	49.01	2461.5	166.7	49.015
Total			100.00	4411.1	340.1	100.000

Sample prepared using catalyst **1** in dichloromethane (Table 1)

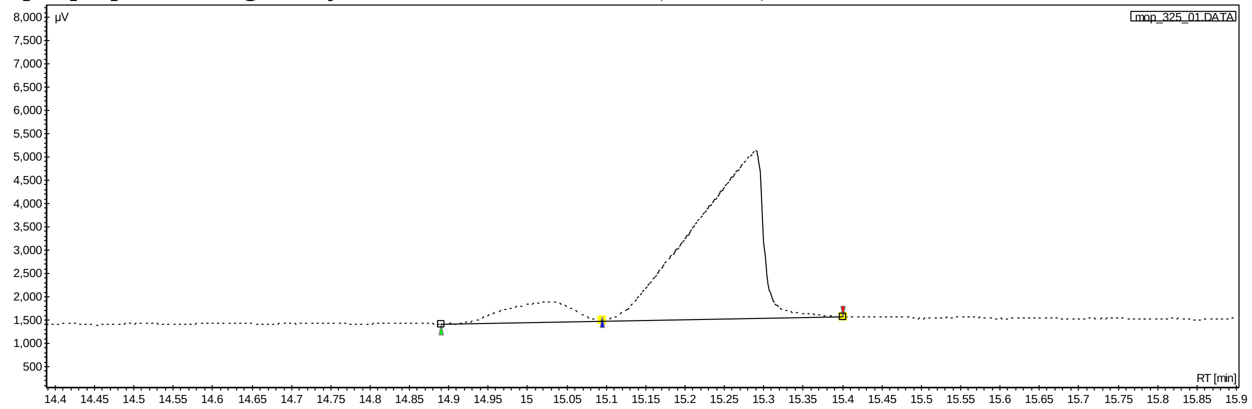

#	Name	Time [Min]	Quantity [% Area]	Height [µV]	Area [µV.Min]	Area % [%]
1	UNKNOWN	15.03	10.95	437.2	45.3	10.951
2	UNKNOWN	15.29	89.05	3599.5	368.5	89.049
Total			100.00	4036.6	413.9	100.000

Sample prepared using catalyst **1** in propylene carbonate (Table 1)

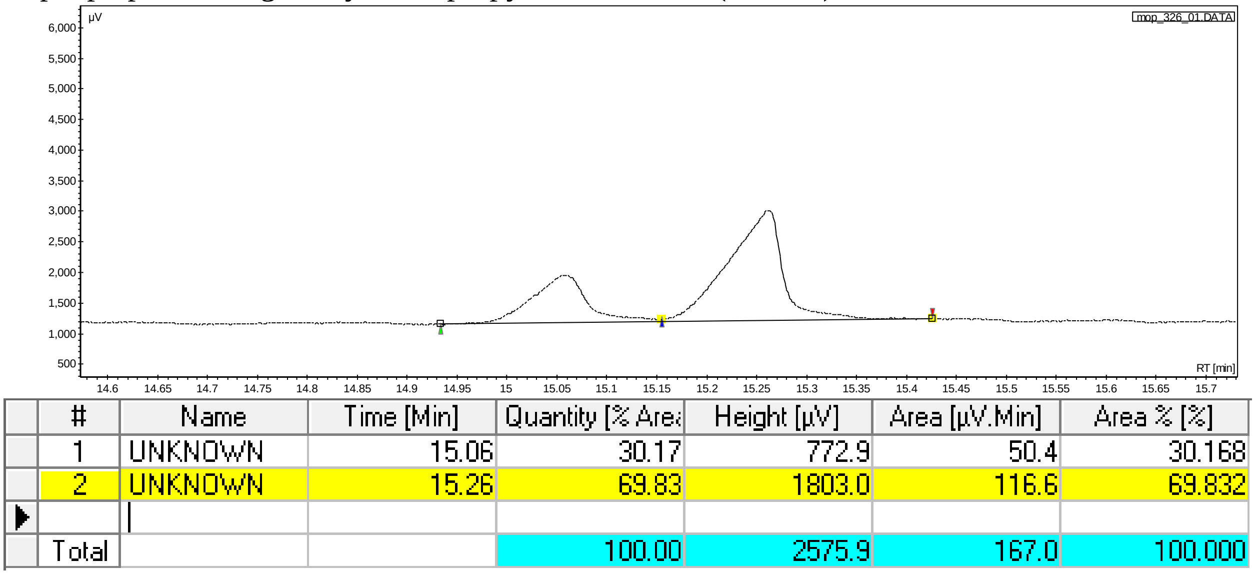

#	Name	Time [Min]	Quantity [% Are	Height [µV]	Area [µV.Min]	Area % [%]
1	UNKNOWN	15.06	30.17	772.9	50.4	30.168
2	UNKNOWN	15.26	69.83	1803.0	116.6	69.832
Total			100.00	2575.9	167.0	100.000

Sample prepared using catalyst **2** in dichloromethane (Table 1)

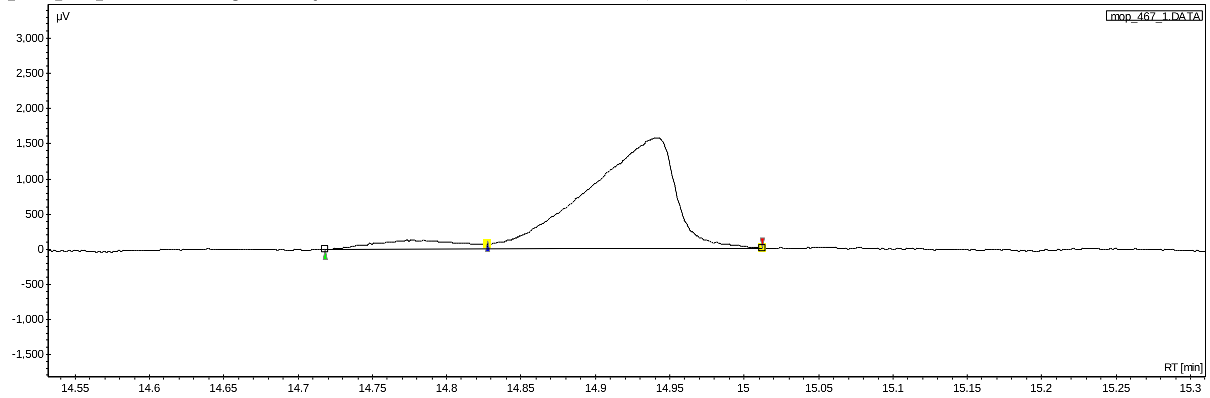

#	Name	Time [Min]	Quantity [% Are	Height [µV]	Area [µV.Min]	Area % [%]
1	UNKNOWN	14.78	7.23	115.5	8.3	7.233
2	UNKNOWN	14.94	92.77	1576.7	106.9	92.767
Total			100.00	1692.2	115.3	100.000

Sample prepared using catalyst **2** in propylene carbonate (Table 1)

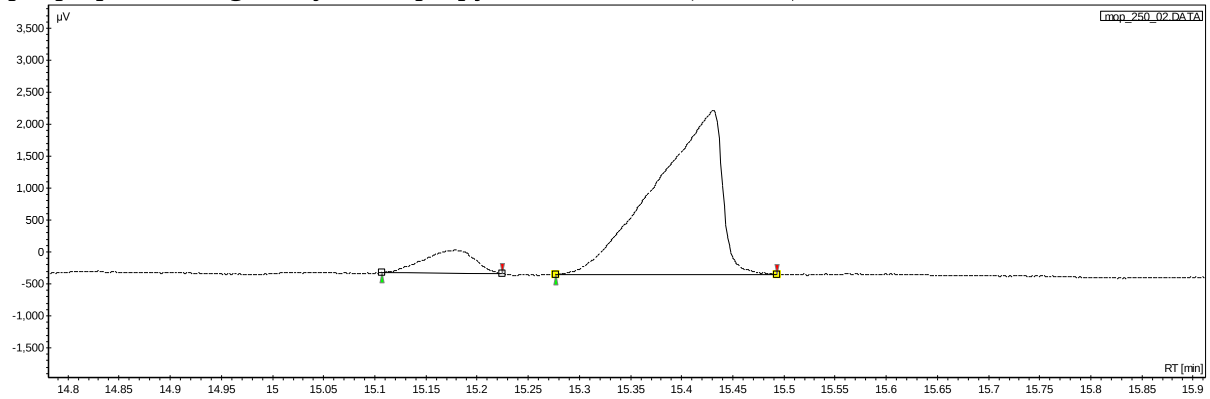

#	Name	Time [Min]	Quantity [% Are	Height [µV]	Area [µV.Min]	Area % [%]
2	UNKNOWN	15.18	9.93	360.4	21.2	9.931
1	UNKNOWN	15.43	90.07	2562.2	192.7	90.069
Total			100.00	2922.6	213.9	100.000

Sample prepared using catalyst **2** in propylene carbonate (Table 2, entry 1)

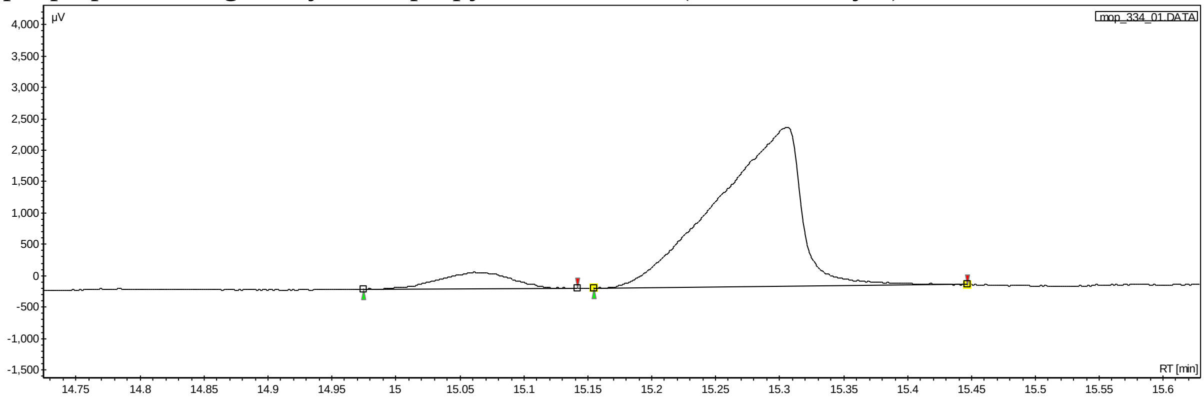

#	Name	Time [Min]	Quantity [% Area]	Height [µV]	Area [µV.Min]	Area % [%]
1	UNKNOWN	15.07	8.34	262.9	17.7	8.341
2	UNKNOWN	15.31	91.66	2537.0	194.0	91.659
Total			100.00	2799.9	211.7	100.000

Sample prepared using catalyst **2** in propylene carbonate (Table 2, entry 2)

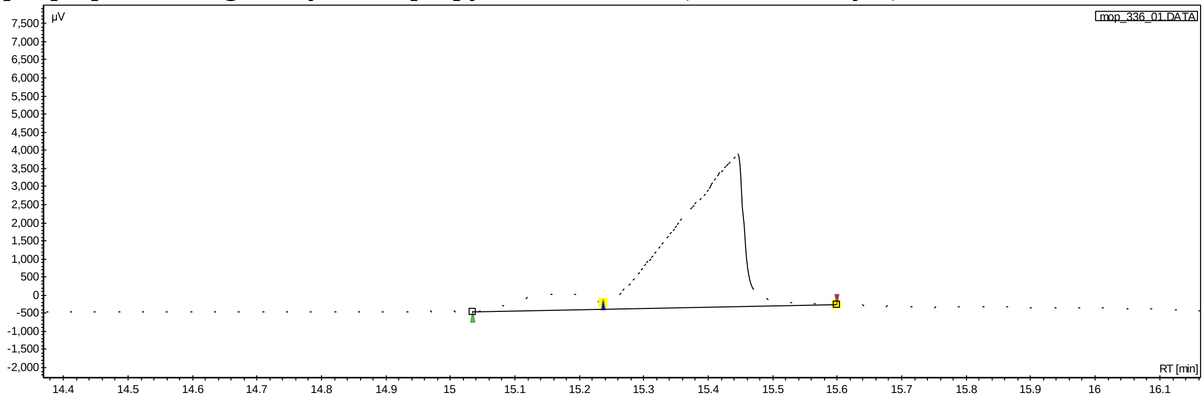

#	Name	Time [Min]	Quantity [% Area]	Height [µV]	Area [µV.Min]	Area % [%]
1	UNKNOWN	15.18	10.14	438.9	55.3	10.144
2	UNKNOWN	15.45	89.86	4189.4	490.2	89.856
Total			100.00	4628.3	545.6	100.000

Sample prepared using catalyst **2** in propylene carbonate (Table 2, entry 3)

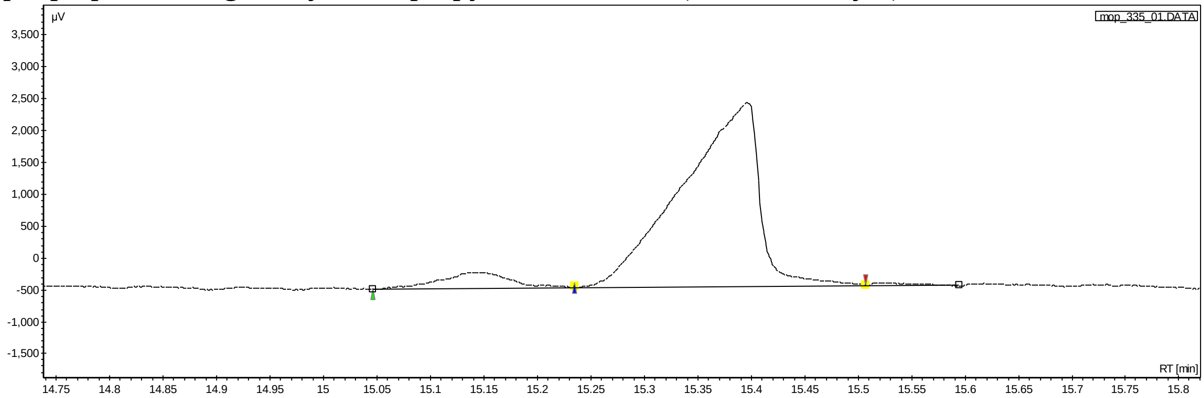

#	Name	Time [Min]	Quantity [% Area]	Height [µV]	Area [µV.Min]	Area % [%]
1	UNKNOWN	15.15	7.54	249.8	20.0	7.544
2	UNKNOWN	15.40	92.46	2899.2	245.0	92.456
Total			100.00	3149.0	265.0	100.000

Sample prepared using catalyst **2** in propylene carbonate (Table 2, entry 4)

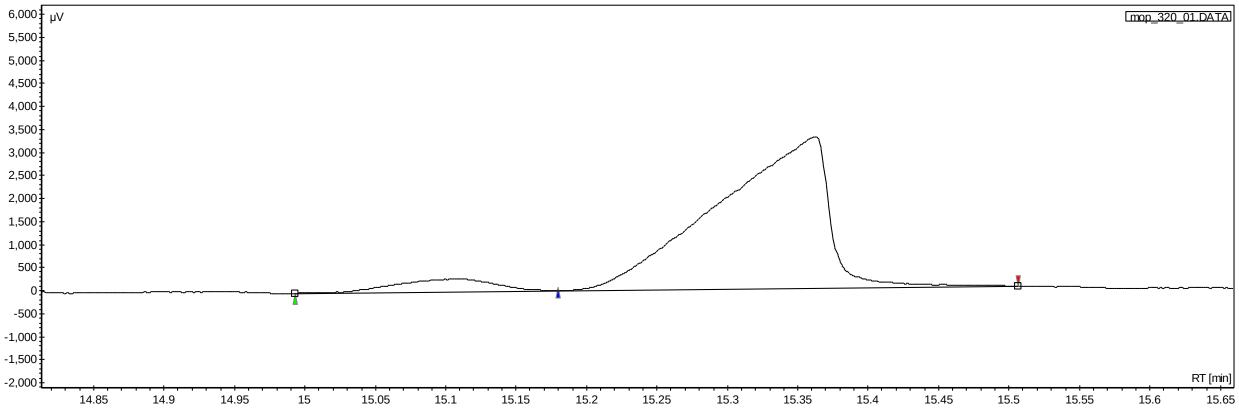

	#	Name	Time [Min]	Quantity [% Are	Height [µV]	Area [µV.Min]	Area % [%]
	1	UNKNOWN	15.11	6.98	279.3	22.7	6.981
	2	UNKNOWN	15.36	93.02	3292.9	302.0	93.019
▶							
	Total			100.00	3572.2	324.6	100.000

Sample prepared using catalyst **2** in propylene carbonate (Table 5, entry 1)

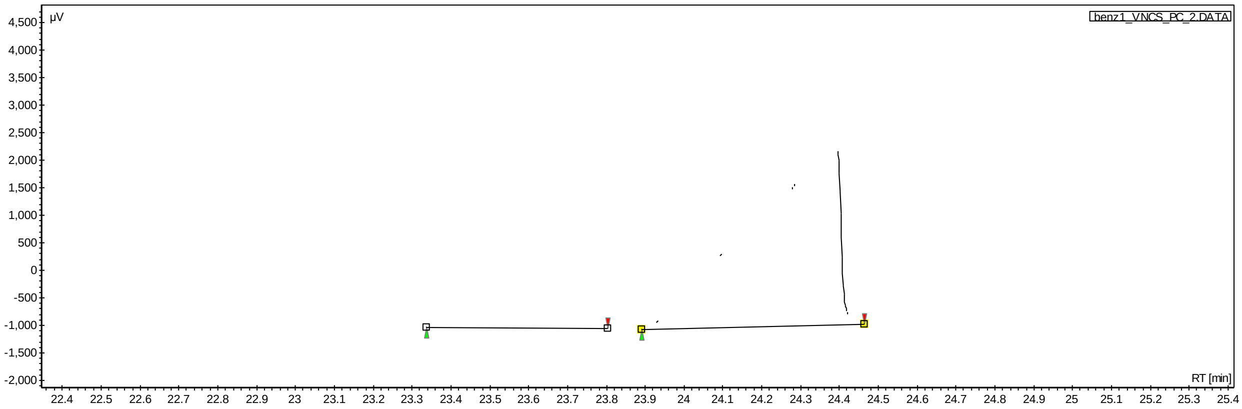

	#	Name	Time [Min]	Quantity [% Are	Height [µV]	Area [µV.Min]	Area % [%]
	2	UNKNOWN	23.58	7.41	240.0	67.6	7.409
	1	UNKNOWN	24.39	92.59	3147.5	845.1	92.591
▶							
	Total			100.00	3387.6	912.7	100.000

Cyanohydrin acetate derived from 4-fluorobenzaldehyde

Analysed using GC method 2.

Racemate

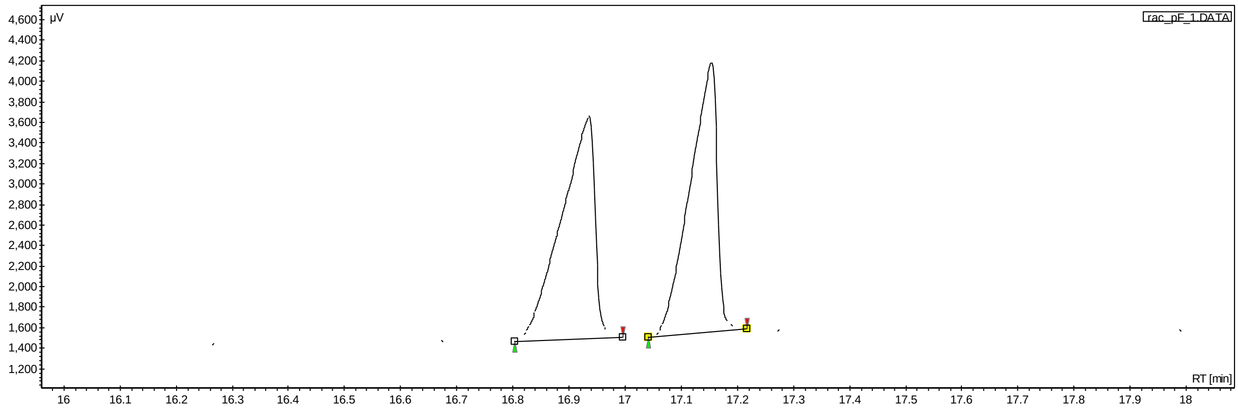

	#	Name	Time [Min]	Quantity [% Area]	Height [µV]	Area [µV.Min]	Area % [%]
	1	UNKNOWN	16.94	50.34	2170.6	149.8	50.339
▶	2	UNKNOWN	17.15	49.66	2628.3	147.8	49.661
	Total			100.00	4798.9	297.6	100.000

Sample prepared using catalyst **1** in dichloromethane (Table 1)

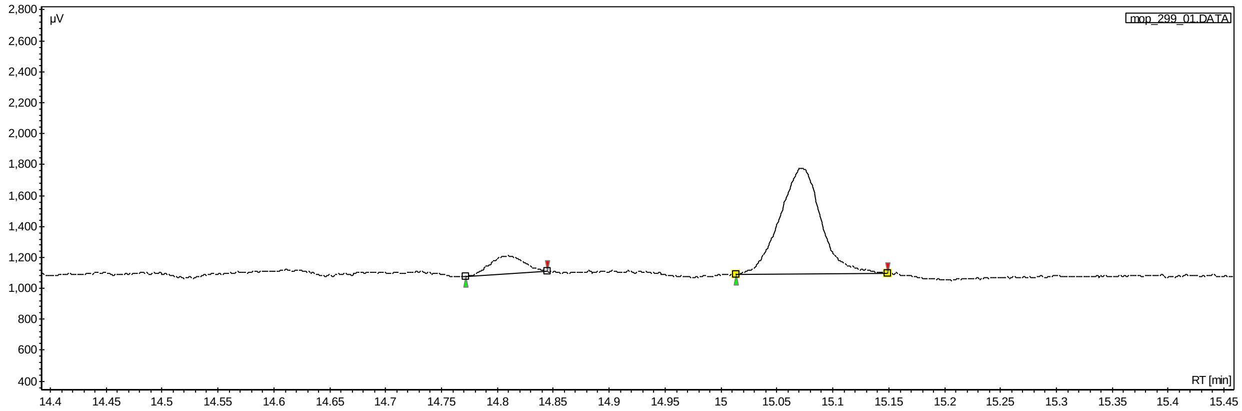

	#	Name	Time [Min]	Quantity [% Area]	Height [µV]	Area [µV.Min]	Area % [%]
	1	UNKNOWN	14.81	12.19	119.6	4.1	12.193
	2	UNKNOWN	15.07	87.81	684.5	29.4	87.807
▶							
	Total			100.00	804.2	33.5	100.000

Sample prepared using catalyst **1** in propylene carbonate (Table 1)

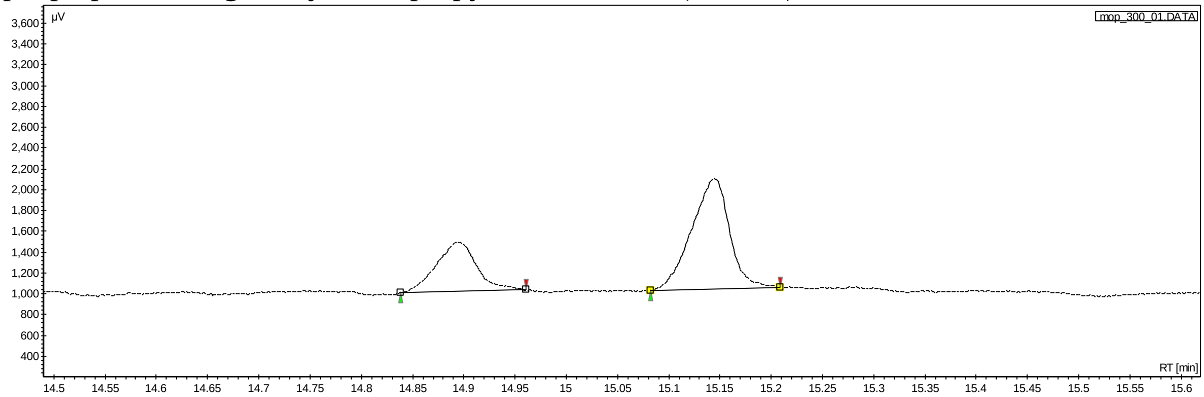

	#	Name	Time [Min]	Quantity [% Are	Height [µV]	Area [µV.Min]	Area % [%]
	1	UNKNOWN	14.90	32.50	477.1	21.8	32.496
	2	UNKNOWN	15.15	67.50	1056.5	45.3	67.504
▶							
	Total			100.00	1533.6	67.2	100.000

Sample prepared using catalyst **2** in dichloromethane (Table 1)

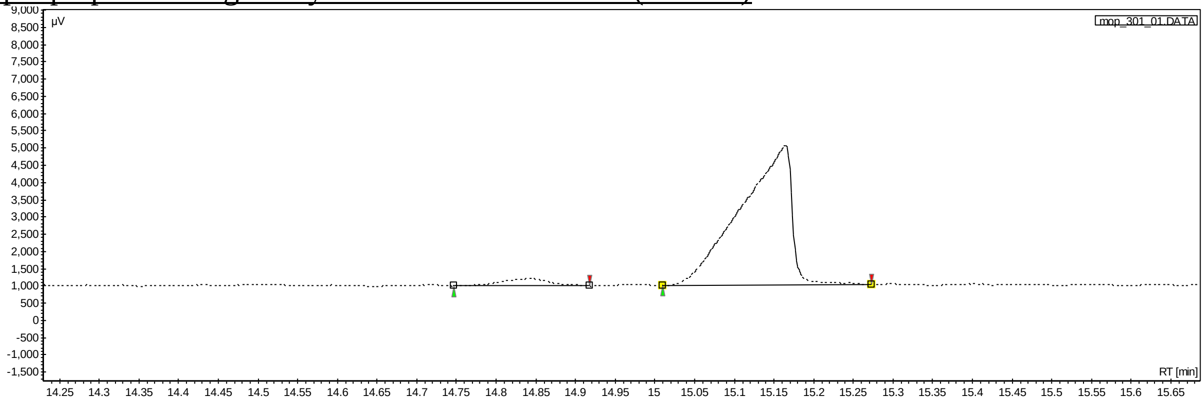

	#	Name	Time [Min]	Quantity [% Are	Height [µV]	Area [µV.Min]	Area % [%]
	1	UNKNOWN	14.85	4.65	212.0	14.8	4.653
	2	UNKNOWN	15.17	95.35	4038.3	304.0	95.347
▶							
	Total			100.00	4250.3	318.9	100.000

Sample prepared using catalyst **2** in propylene carbonate (Table 1)

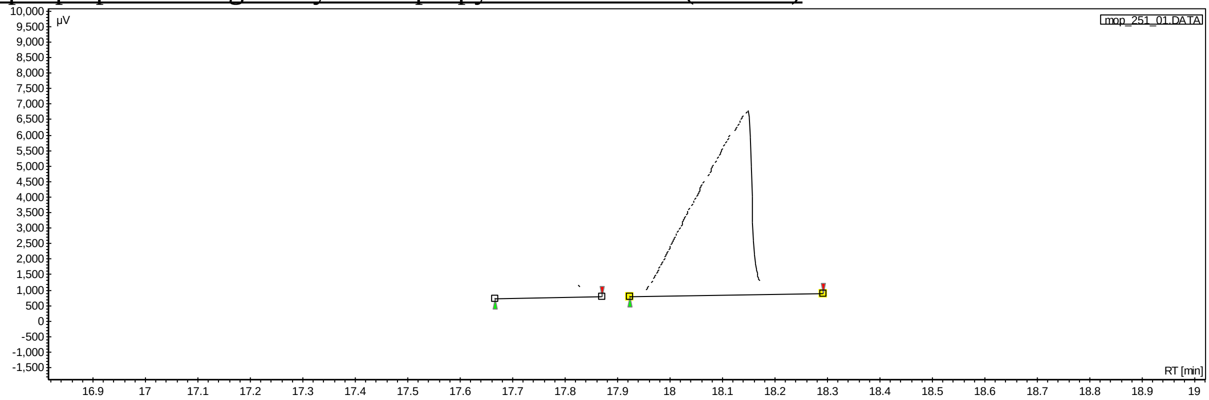

	#	Name	Time [Min]	Quantity [% Are	Height [µV]	Area [µV.Min]	Area % [%]
	1	UNKNOWN	17.77	12.01	902.0	93.9	12.011
	2	UNKNOWN	18.15	87.99	5918.9	688.1	87.989
▶							
	Total			100.00	6820.9	782.1	100.000

Sample prepared using catalyst **2** in propylene carbonate (Table 2, entry 5)

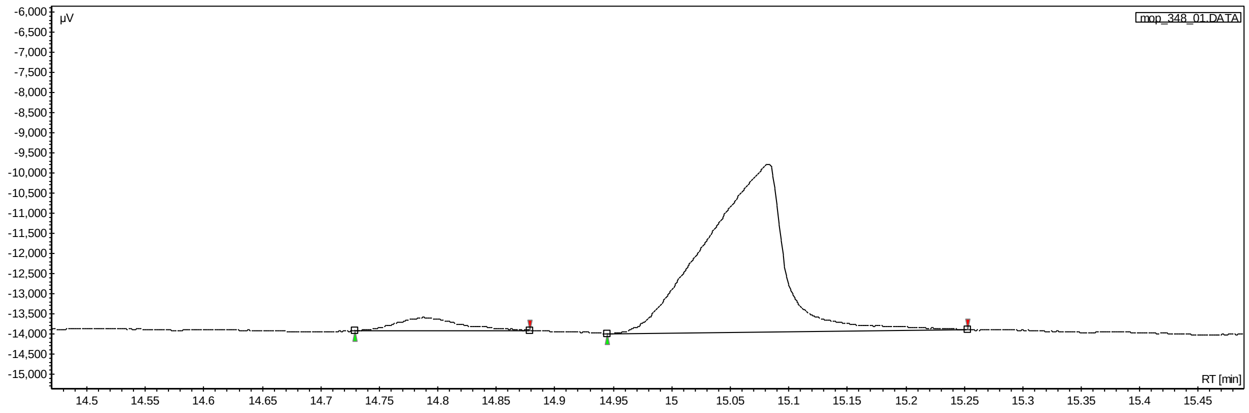

#	Name	Time [Min]	Quantity [% Are	Height [µV]	Area [µV.Min]	Area % [%]
1	UNKNOWN	14.79	5.97	325.9	20.5	5.966
2	UNKNOWN	15.08	94.03	4179.9	323.0	94.034
Total			100.00	4505.8	343.5	100.000

Sample prepared using catalyst **2** in propylene carbonate (Table 5, entry 5)

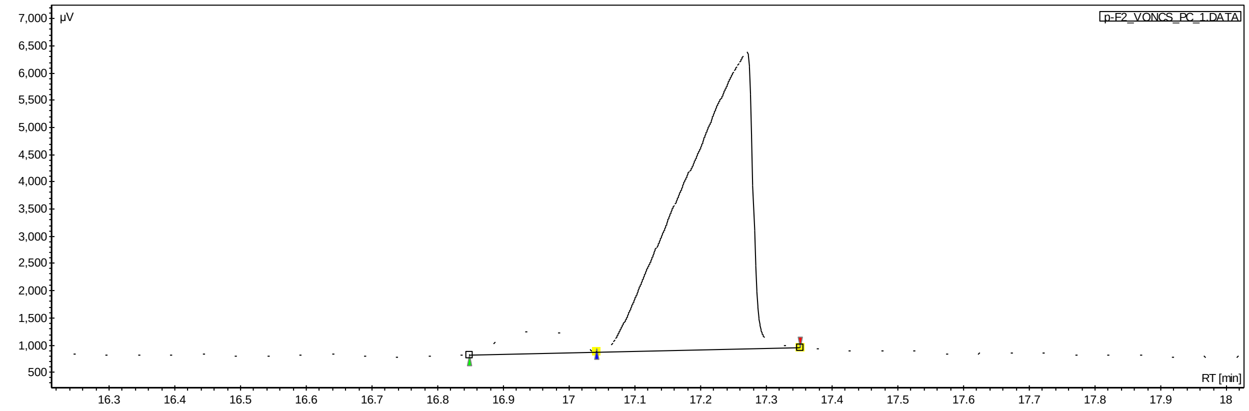

#	Name	Time [Min]	Quantity [% Are	Height [µV]	Area [µV.Min]	Area % [%]
1	UNKNOWN	16.97	7.87	471.9	68.4	7.873
2	UNKNOWN	17.31	92.13	6087.2	800.6	92.127
Total			100.00	6559.1	869.0	100.000

Cyanohydrin acetate derived from 4-chlorobenzaldehyde

Analysed using GC method 3.

Racemate

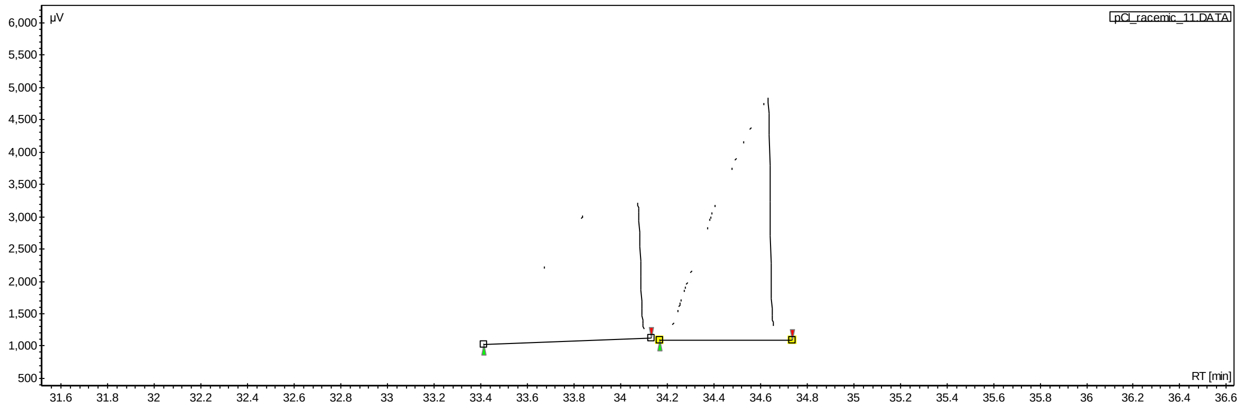

#	Name	Time [Min]	Quantity [% Area]	Height [µV]	Area [µV.Min]	Area % [%]
1	UNKNOWN	33.96	50.12	2398.3	940.1	50.122
2	UNKNOWN	34.63	49.88	3767.0	935.6	49.878
Total			100.00	6165.3	1875.7	100.000

Sample prepared using catalyst **1** in dichloromethane (Table 1)

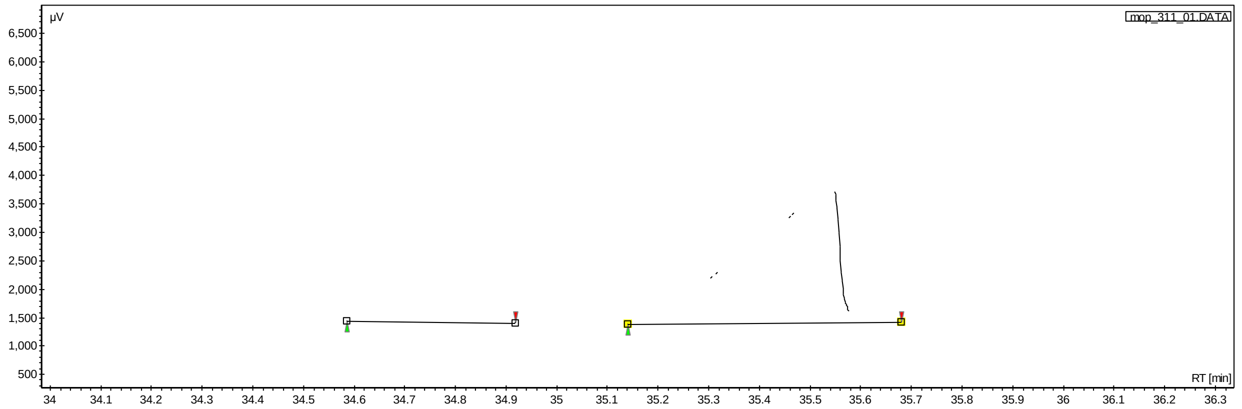

#	Name	Time [Min]	Quantity [% Area]	Height [µV]	Area [µV.Min]	Area % [%]
1	UNKNOWN	34.76	8.32	267.9	43.8	8.320
2	UNKNOWN	35.55	91.68	2310.5	483.0	91.680
Total			100.00	2578.3	526.8	100.000

Sample prepared using catalyst **1** in propylene carbonate (Table 1)

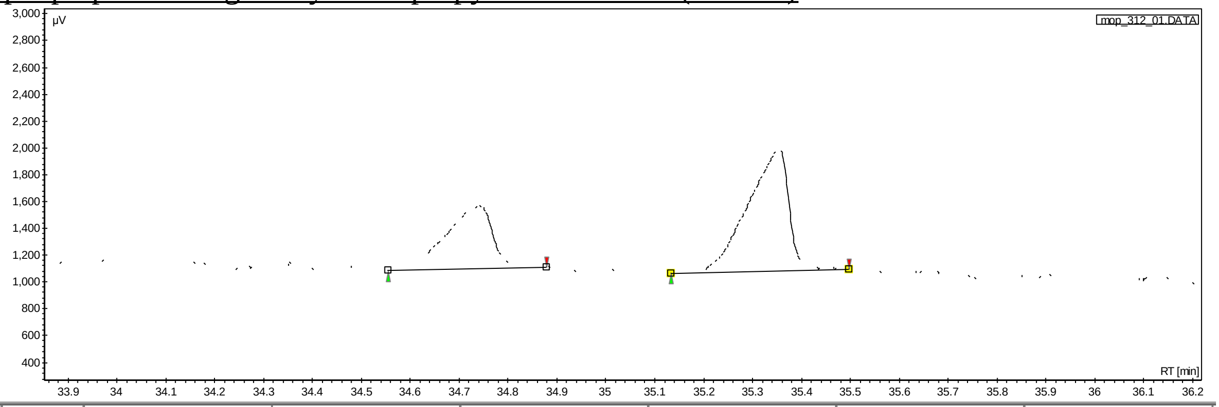

#	Name	Time [Min]	Quantity [% Area	Height [µV]	Area [µV.Min]	Area % [%]
1	UNKNOWN	34.74	37.48	469.6	52.9	37.479
2	UNKNOWN	35.35	62.52	900.4	88.3	62.521
Total			100.00	1370.0	141.2	100.000

Sample prepared using catalyst **2** in dichloromethane (Table 1)

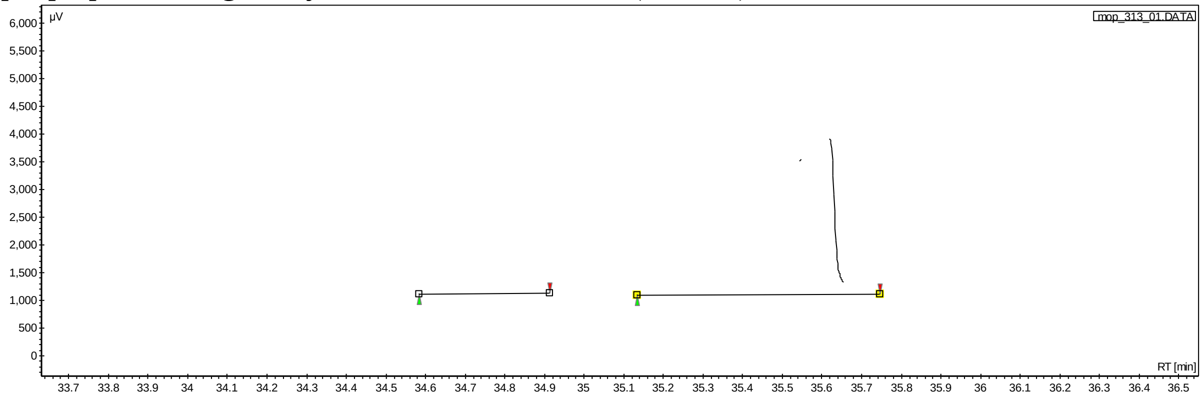

#	Name	Time [Min]	Quantity [% Area	Height [µV]	Area [µV.Min]	Area % [%]
2	UNKNOWN	34.79	3.47	114.9	24.9	3.471
1	UNKNOWN	35.62	96.53	2789.3	692.9	96.529
Total			100.00	2904.2	717.8	100.000

Sample prepared using catalyst **2** in propylene carbonate (Table 1)

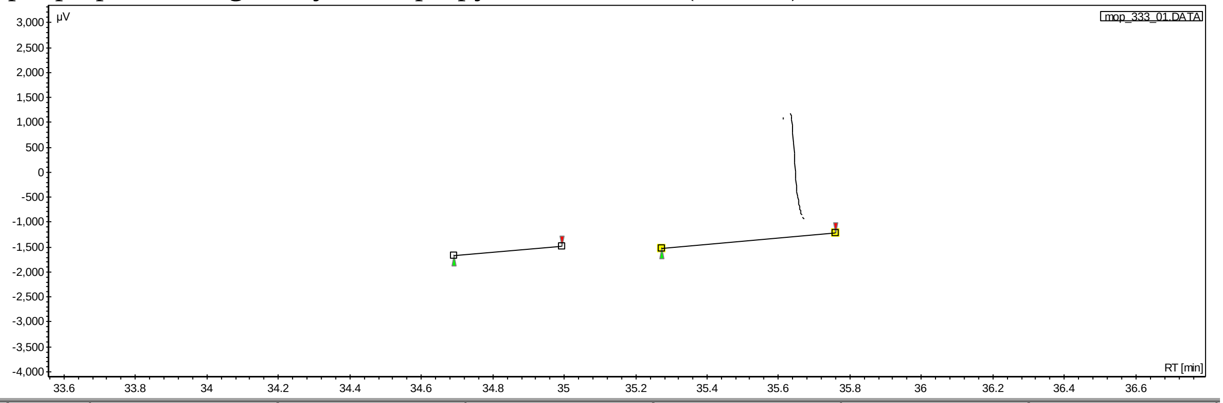

#	Name	Time [Min]	Quantity [% Area	Height [µV]	Area [µV.Min]	Area % [%]
1	UNKNOWN	34.88	12.15	446.4	69.2	12.153
2	UNKNOWN	35.63	87.85	2487.2	500.1	87.847
Total			100.00	2933.6	569.2	100.000

Sample prepared using catalyst **2** in propylene carbonate (Table 2, entry 7)

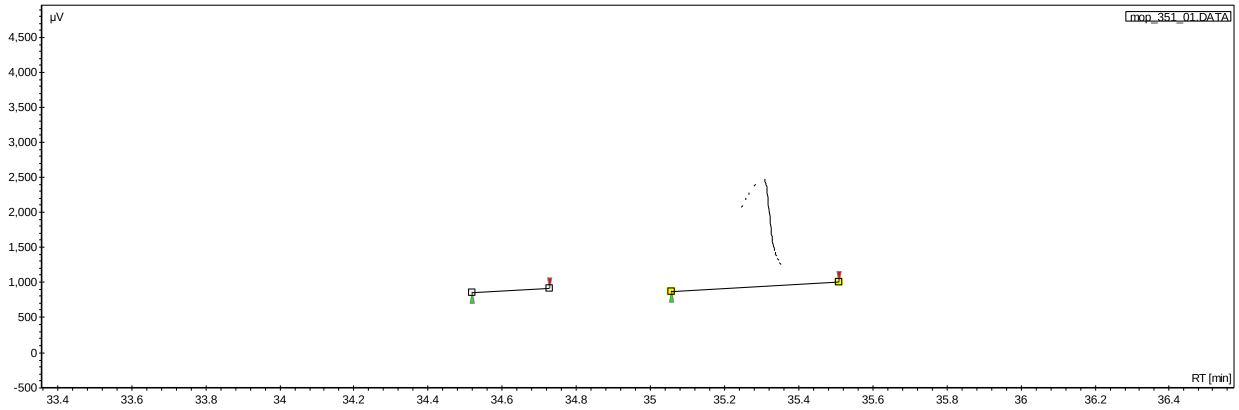

#	Name	Time [Min]	Quantity [% Area	Height [µV]	Area [µV.Min]	Area % [%]
1	UNKNOWN	34.62	10.05	258.1	24.7	10.051
2	UNKNOWN	35.30	89.95	1554.5	221.5	89.949
Total			100.00	1812.6	246.2	100.000

Sample prepared using catalyst **2** in propylene carbonate (Table 5, entry 3)

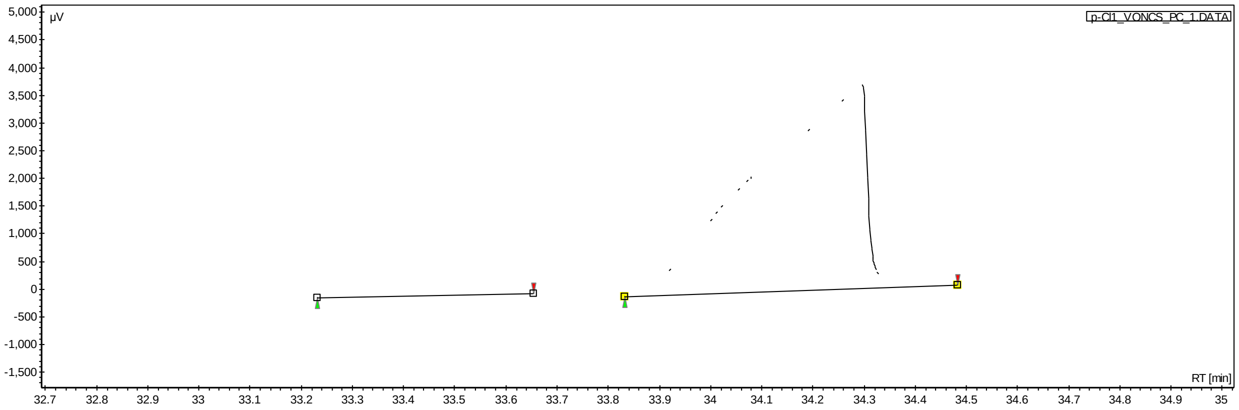

#	Name	Time [Min]	Quantity [% Area	Height [µV]	Area [µV.Min]	Area % [%]
1	UNKNOWN	33.49	13.02	577.3	135.7	13.019
2	UNKNOWN	34.30	86.98	3689.2	906.7	86.981
Total			100.00	4266.5	1042.4	100.000

Cyanohydrin acetate derived from 3-chlorobenzaldehyde (Analysed using GC methods 3 or 4).

Racemate (method 3)

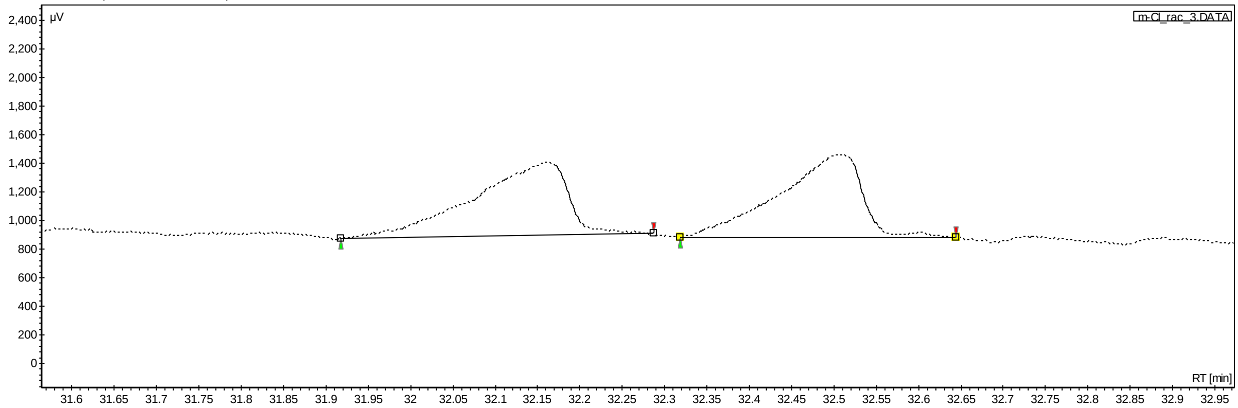

#	Name	Time [Min]	Quantity [% Area]	Height [µV]	Area [µV.Min]	Area % [%]
1	UNKNOWN	32.16	49.65	513.7	66.1	49.646
2	UNKNOWN	32.51	50.35	583.5	67.0	50.354
Total			100.00	1097.1	133.1	100.000

Racemate (method 4)

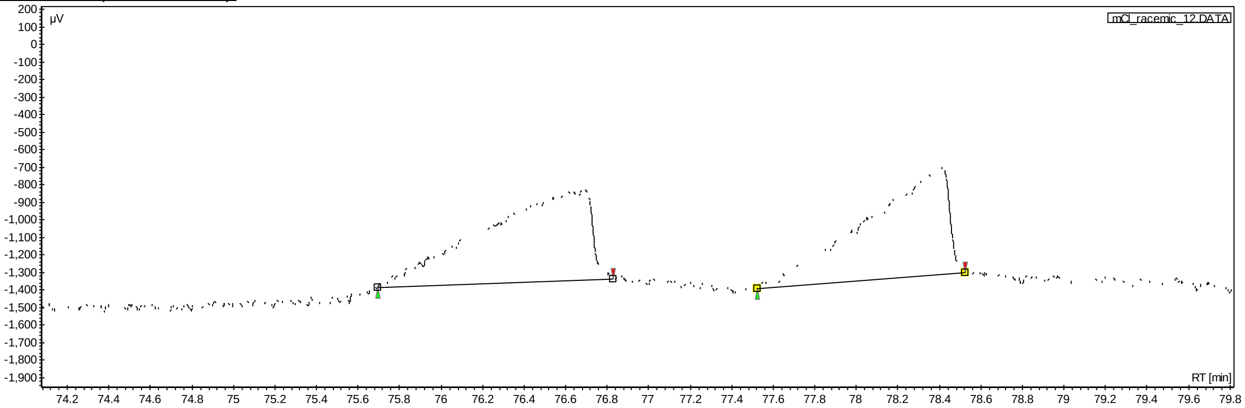

#	Name	Time [Min]	Quantity [% Area]	Height [µV]	Area [µV.Min]	Area % [%]
2	UNKNOWN	76.68	52.60	528.2	311.1	52.598
1	UNKNOWN	78.41	47.40	607.6	280.4	47.402
Total			100.00	1135.8	591.5	100.000

Sample prepared using catalyst **1** in dichloromethane (Table 1)

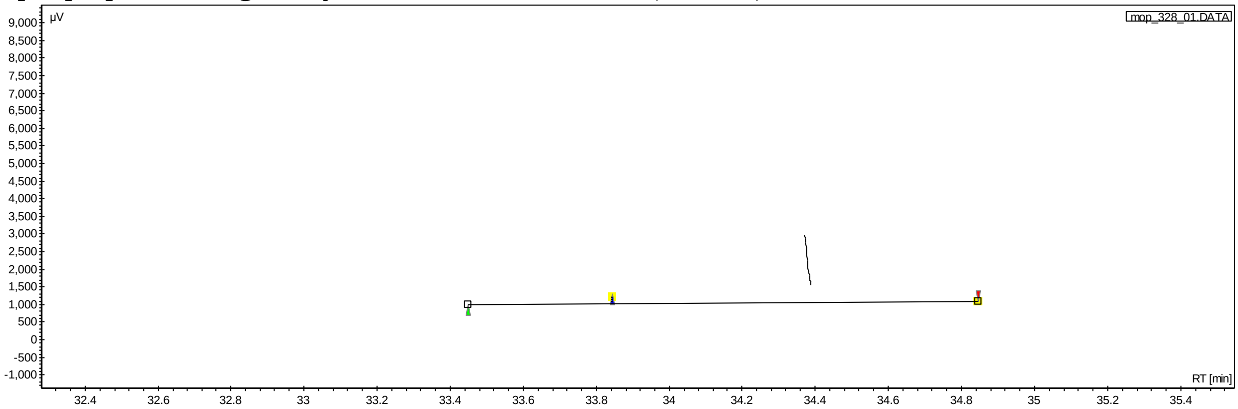

#	Name	Time [Min]	Quantity [% Area]	Height [µV]	Area [µV.Min]	Area % [%]
1	UNKNOWN	33.84	7.76	192.7	48.7	7.761
2	UNKNOWN	34.36	92.24	1973.8	579.0	92.239
Total			100.00	2166.5	627.7	100.000

Sample prepared using catalyst **1** in propylene carbonate (Table 1)

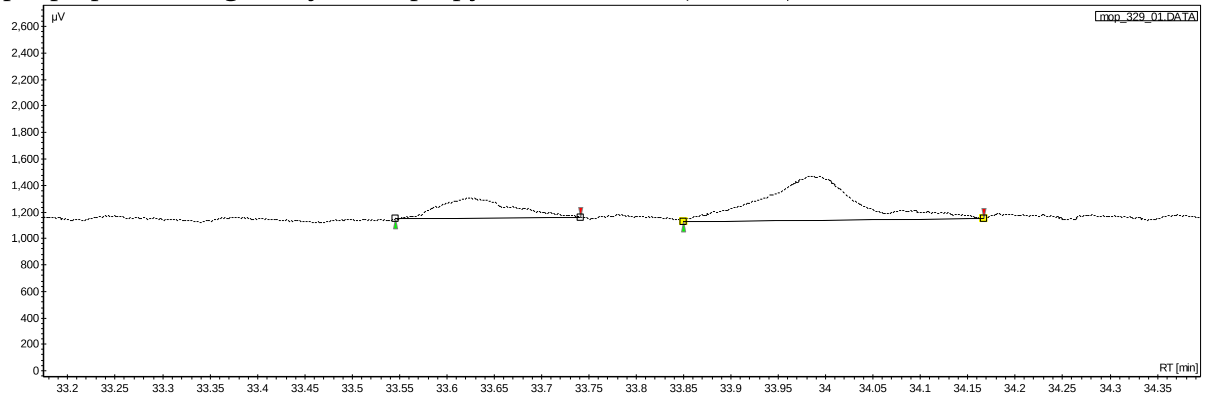

	#	Name	Time [Min]	Quantity [% Area]	Height [µV]	Area [µV.Min]	Area % [%]
	1	UNKNOWN	33.62	26.76	150.2	13.9	26.759
	2	UNKNOWN	33.99	73.24	327.5	38.0	73.241
▶							
	Total			100.00	477.7	51.8	100.000

Sample prepared using catalyst **2** in dichloromethane (Table 1)

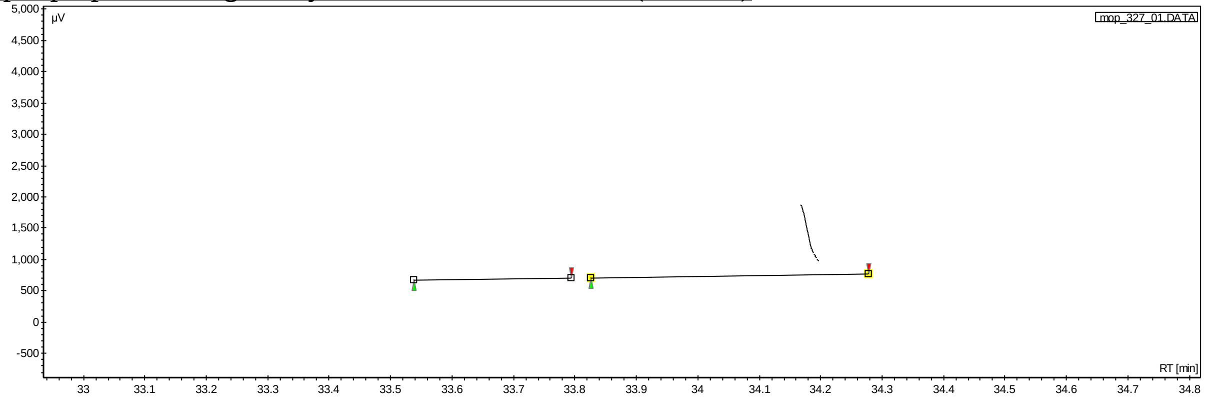

	#	Name	Time [Min]	Quantity [% Area]	Height [µV]	Area [µV.Min]	Area % [%]
	2	UNKNOWN	33.63	5.64	95.1	12.0	5.637
	1	UNKNOWN	34.16	94.36	1154.1	200.2	94.363
▶							
	Total			100.00	1249.1	212.2	100.000

Sample prepared using catalyst **2** in propylene carbonate (Table 1)

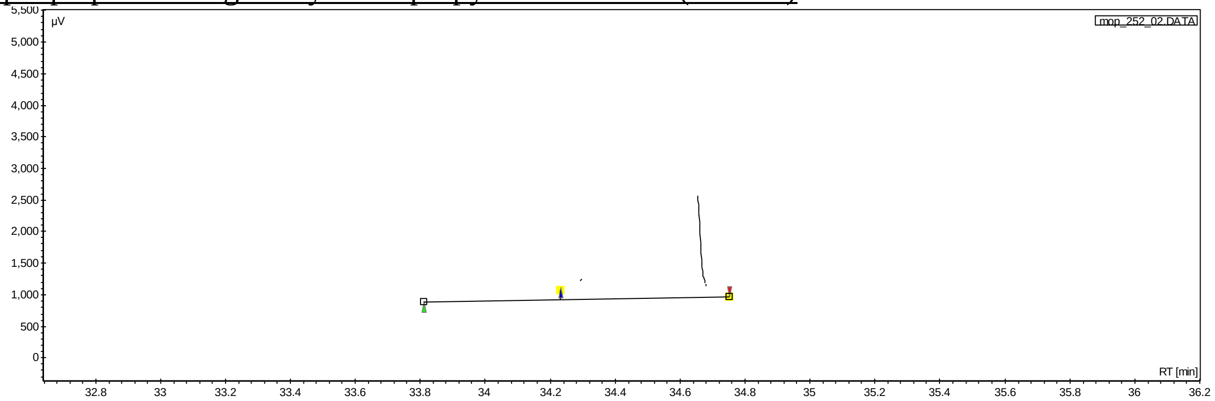

	#	Name	Time [Min]	Quantity [% Area]	Height [µV]	Area [µV.Min]	Area % [%]
	1	UNKNOWN	34.14	18.81	320.1	91.6	18.814
	2	UNKNOWN	34.65	81.19	1634.3	395.1	81.186
▶							
	Total			100.00	1954.4	486.7	100.000

Sample prepared using catalyst **2** in propylene carbonate (Table 2, entry 6)

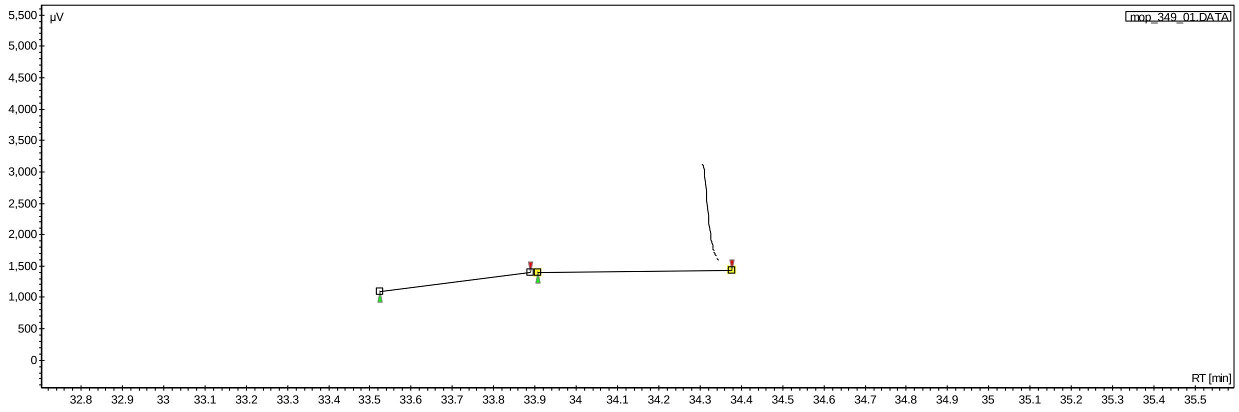

	#	Name	Time [Min]	Quantity [% Are	Height [µV]	Area [µV.Min]	Area % [%]
	1	UNKNOWN	33.74	9.14	161.6	36.5	9.142
	2	UNKNOWN	34.30	90.86	1706.8	362.6	90.858
▶							
	Total			100.00	1868.4	399.0	100.000

Sample prepared using catalyst **2** in propylene carbonate (Table 5, entry 12)

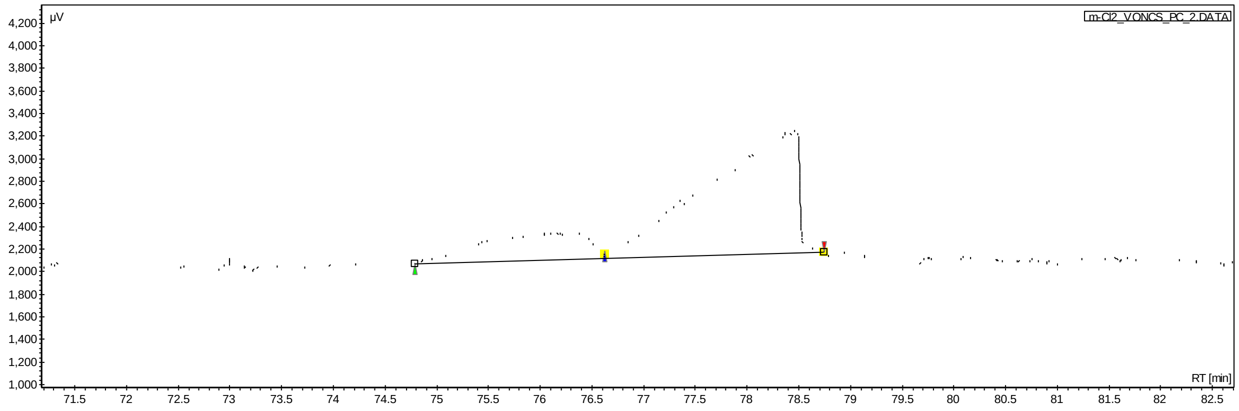

	#	Name	Time [Min]	Quantity [% Are	Height [µV]	Area [µV.Min]	Area % [%]
	1	UNKNOWN	76.25	21.28	245.5	298.3	21.282
	2	UNKNOWN	78.47	78.72	1108.7	1103.4	78.718
▶							
	Total			100.00	1354.2	1401.8	100.000

Cyanohydrin acetate derived from 2-methylbenzaldehyde

Analysed using GC method 3.

Racemate

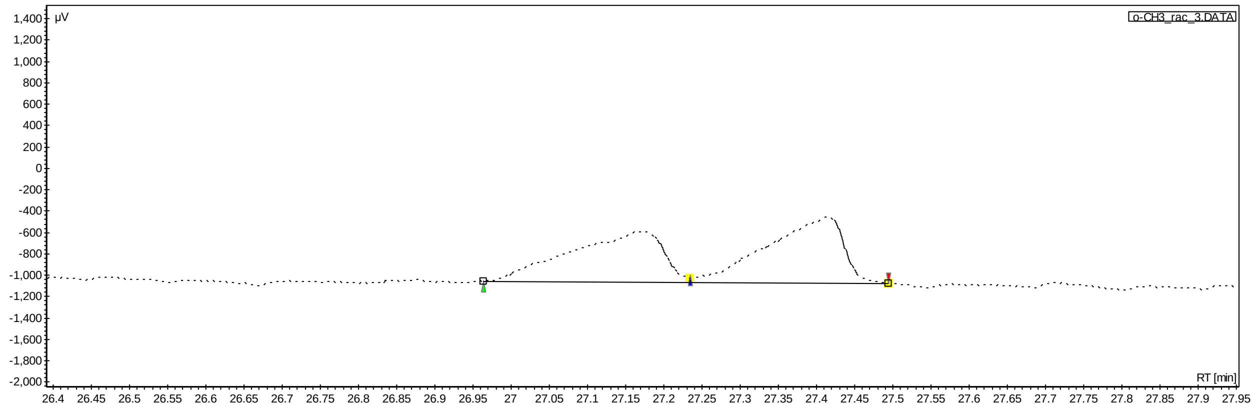

	#	Name	Time [Min]	Quantity [% Area]	Height [µV]	Area [µV.Min]	Area % [%]
	1	UNKNOWN	27.17	48.38	473.3	67.3	48.382
	2	UNKNOWN	27.42	51.62	619.3	71.8	51.618
▶							
	Total			100.00	1092.6	139.1	100.000

Sample prepared using catalyst **1** in dichloromethane (Table 1)

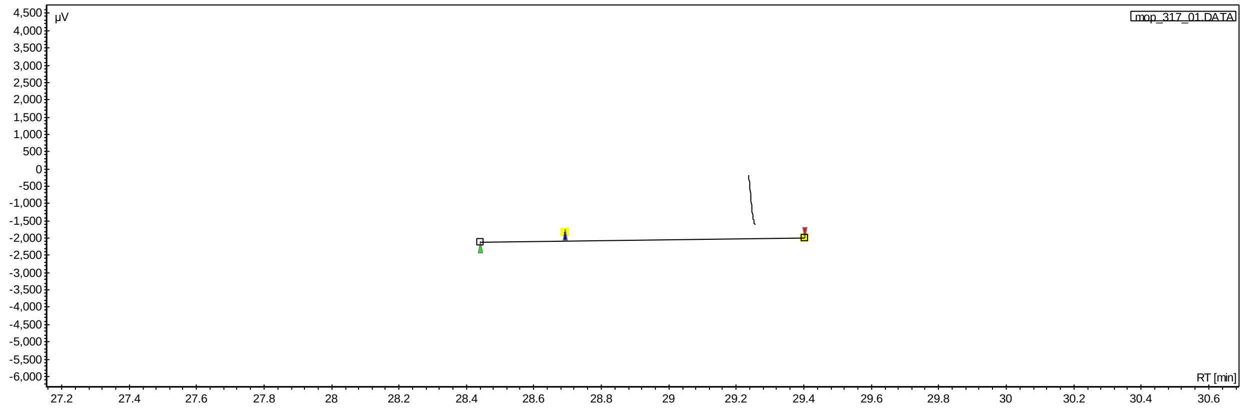

	#	Name	Time [Min]	Quantity [% Area]	Height [µV]	Area [µV.Min]	Area % [%]
	1	UNKNOWN	28.69	5.72	256.1	36.5	5.724
	2	UNKNOWN	29.23	94.28	1891.2	601.3	94.276
▶							
	Total			100.00	2147.3	637.8	100.000

Sample prepared using catalyst **1** in propylene carbonate (Table 1)

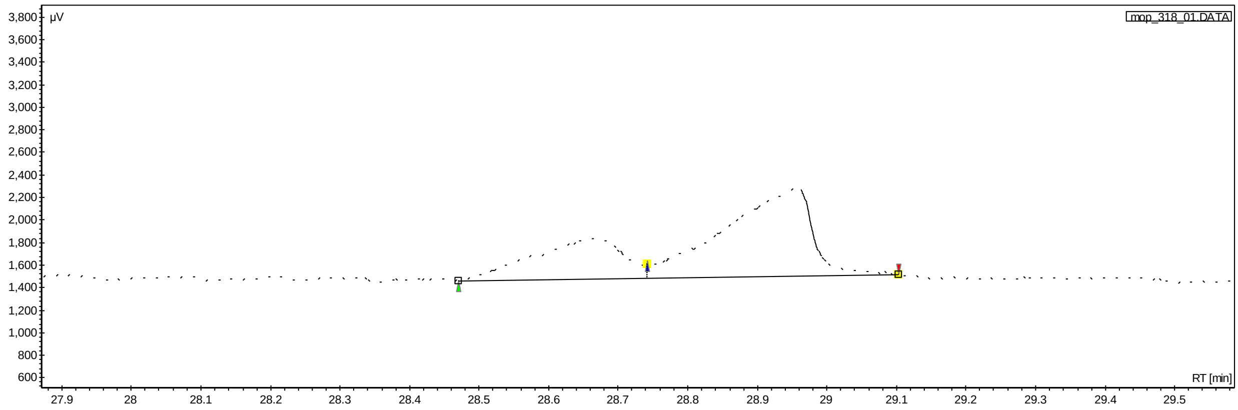

	#	Name	Time [Min]	Quantity [% Area]	Height [μV]	Area [μV.Min]	Area % [%]
	1	UNKNOWN	28.67	31.94	361.8	54.0	31.943
	2	UNKNOWN	28.96	68.06	782.9	115.0	68.057
▶							
	Total			100.00	1144.7	168.9	100.000

Sample prepared using catalyst **2** in dichloromethane (Table 1)

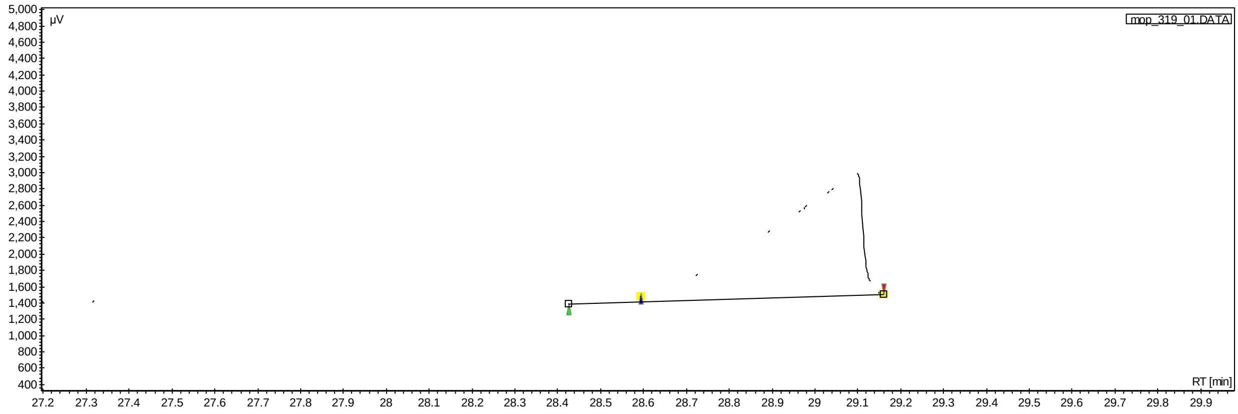

	#	Name	Time [Min]	Quantity [% Area]	Height [μV]	Area [μV.Min]	Area % [%]
	1	UNKNOWN	28.56	2.20	77.4	8.6	2.195
	2	UNKNOWN	29.10	97.80	1490.6	384.3	97.805
▶							
	Total			100.00	1568.1	392.9	100.000

Sample prepared using catalyst **2** in propylene carbonate (Table 1)

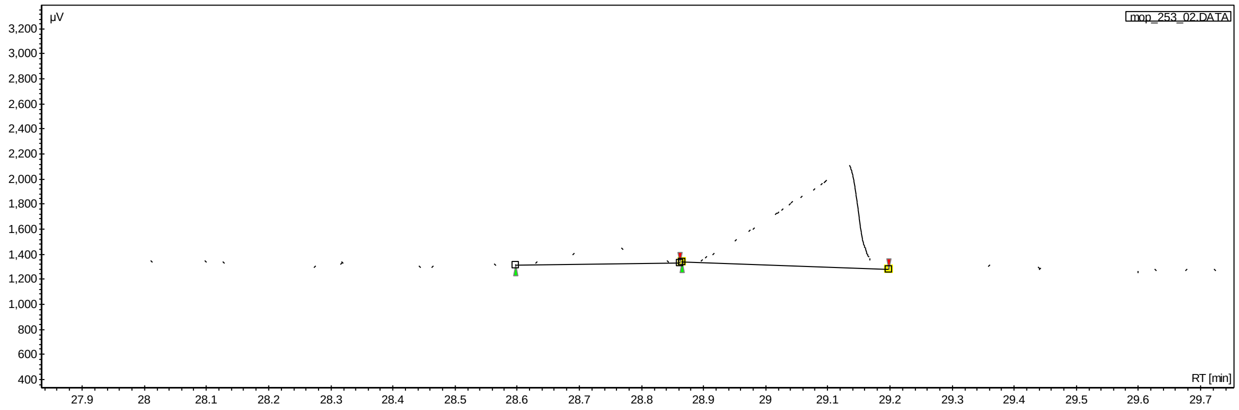

#	Name	Time [Min]	Quantity [% Area	Height [µV]	Area [µV.Min]	Area % [%]
1	UNKNOWN	28.74	13.68	127.2	18.0	13.675
2	UNKNOWN	29.13	86.32	825.4	113.5	86.325
Total			100.00	952.6	131.5	100.000

Sample prepared using catalyst **2** in propylene carbonate (Table 2, entry 9)

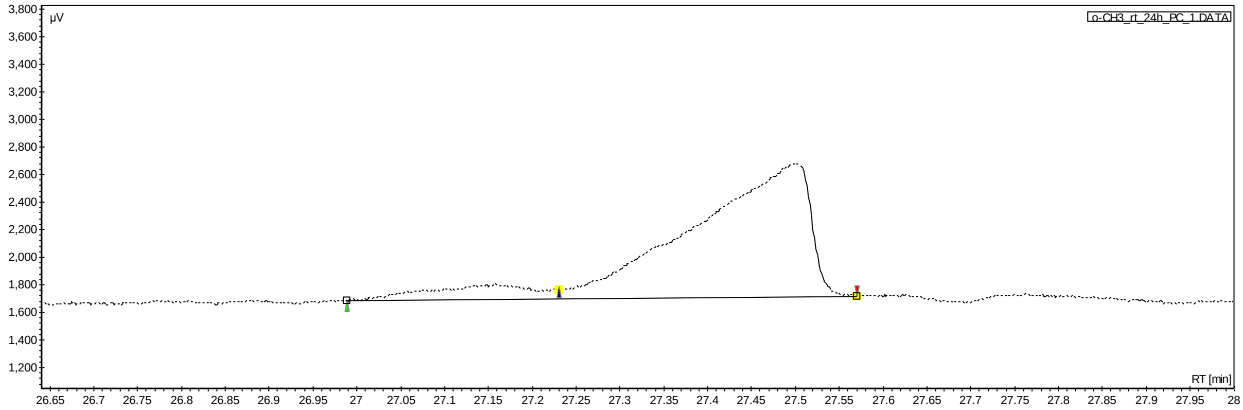

#	Name	Time [Min]	Quantity [% Area	Height [µV]	Area [µV.Min]	Area % [%]
1	UNKNOWN	27.16	9.56	100.3	15.4	9.558
2	UNKNOWN	27.50	90.44	961.7	145.3	90.442
Total			100.00	1062.0	160.6	100.000

Cyanohydrin acetate derived from 3-methylbenzaldehyde

Analysed using GC method 5.

Racemate

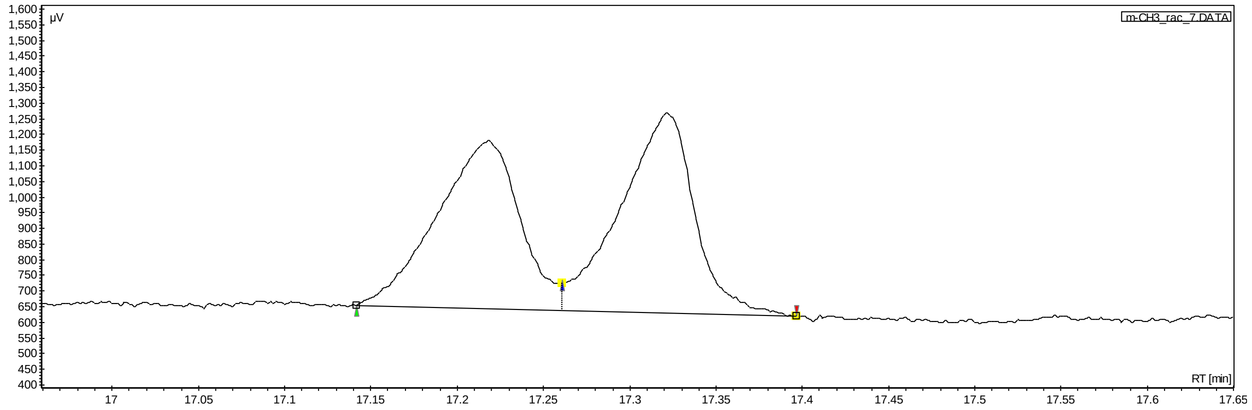

	#	Name	Time [Min]	Quantity [% Area]	Height [µV]	Area [µV.Min]	Area % [%]
	1	UNKNOWN	17.22	48.45	535.5	29.8	48.450
	2	UNKNOWN	17.32	51.55	638.7	31.7	51.550
▶							
	Total			100.00	1174.2	61.6	100.000

Sample prepared using catalyst **1** in dichloromethane (Table 1)

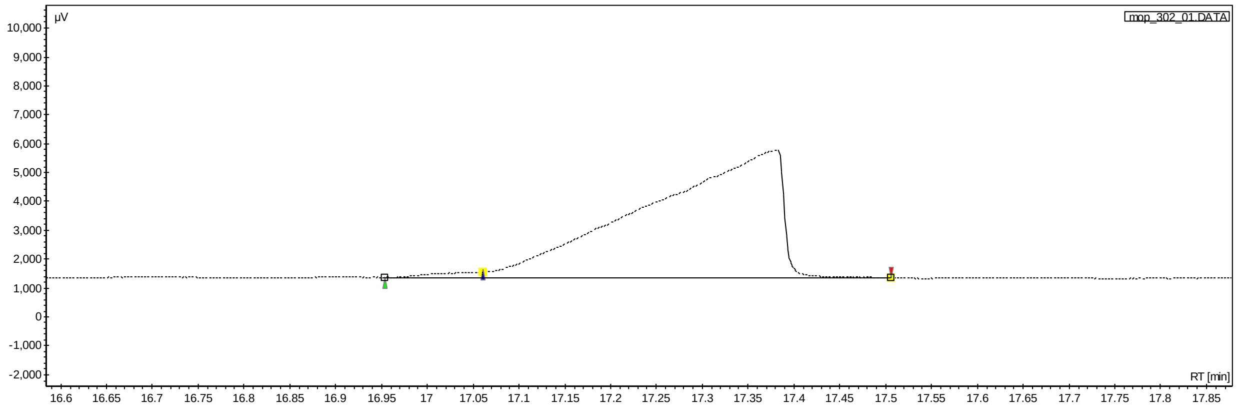

	#	Name	Time [Min]	Quantity [% Area]	Height [µV]	Area [µV.Min]	Area % [%]
	1	UNKNOWN	17.06	1.45	180.4	11.1	1.450
	2	UNKNOWN	17.38	98.55	4434.4	751.2	98.550
▶							
	Total			100.00	4614.9	762.3	100.000

Sample prepared using catalyst **1** in propylene carbonate (Table 1)

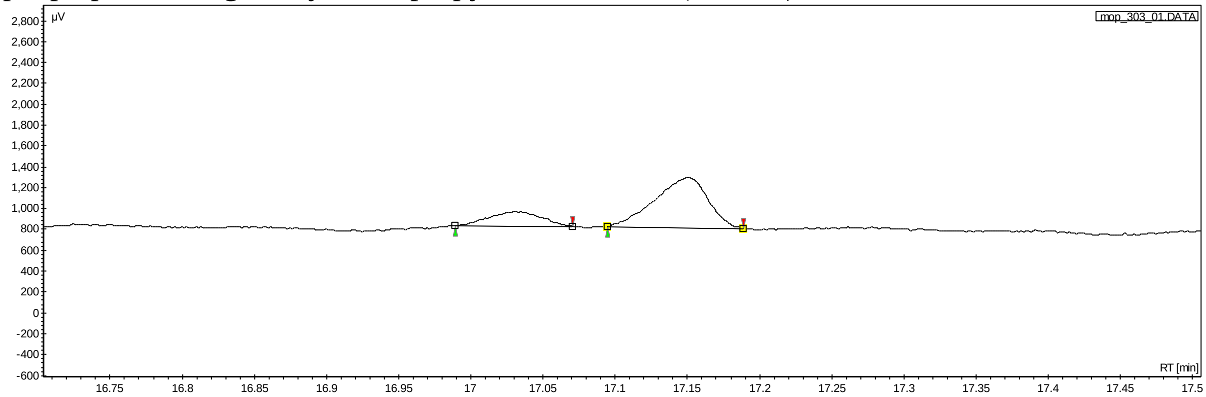

#	Name	Time [Min]	Quantity [% Are	Height [μV]	Area [μV.Min]	Area % [%]
1	UNKNOWN	17.03	21.68	136.6	5.7	21.679
2	UNKNOWN	17.15	78.32	486.1	20.5	78.321
Total			100.00	622.6	26.2	100.000

Sample prepared using catalyst **2** in dichloromethane (Table 1)

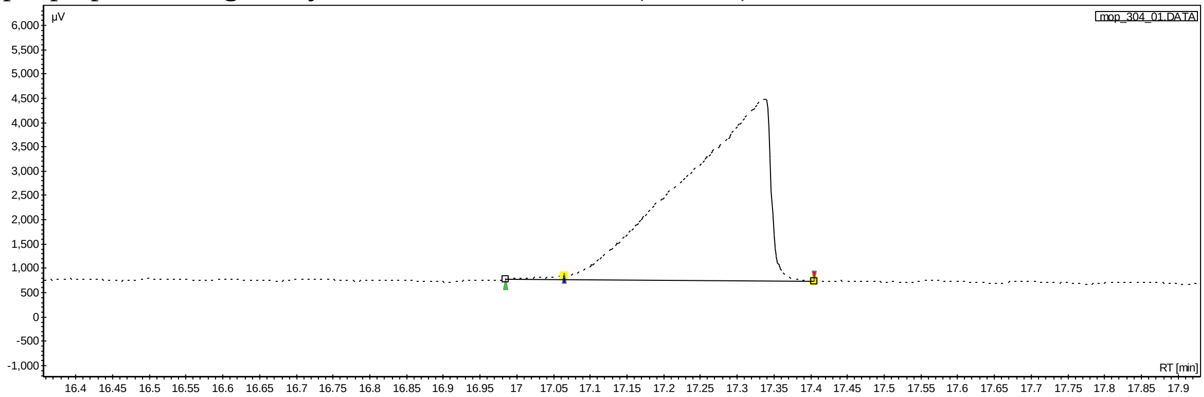

#	Name	Time [Min]	Quantity [% Are	Height [μV]	Area [μV.Min]	Area % [%]
1	UNKNOWN	17.06	0.59	77.2	3.1	0.590
2	UNKNOWN	17.34	99.41	3749.6	515.8	99.410
Total			100.00	3826.8	518.9	100.000

Sample prepared using catalyst **2** in propylene carbonate (Table 1)

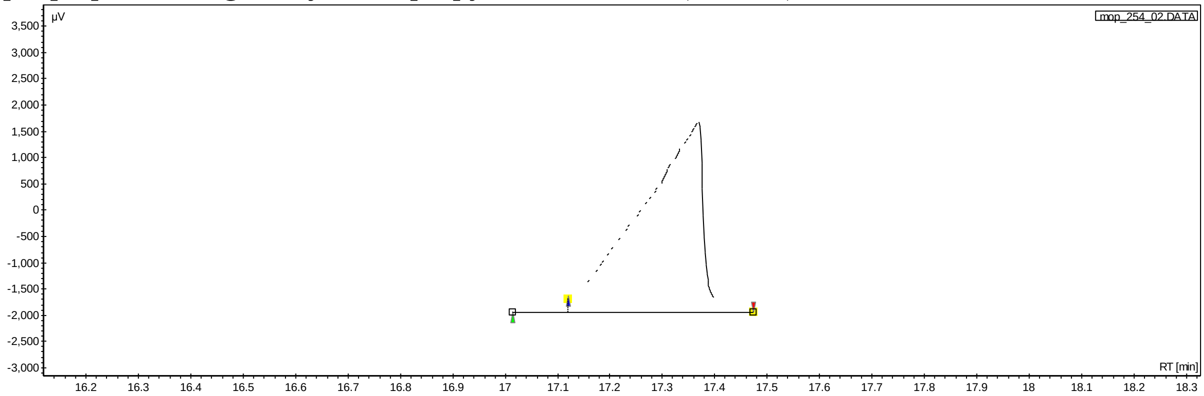

#	Name	Time [Min]	Quantity [% Are	Height [μV]	Area [μV.Min]	Area % [%]
1	UNKNOWN	17.12	3.49	248.9	17.9	3.488
2	UNKNOWN	17.37	96.51	3623.0	494.3	96.512
Total			100.00	3871.9	512.2	100.000

Sample prepared using catalyst **2** in propylene carbonate (Table 2, entry 10)

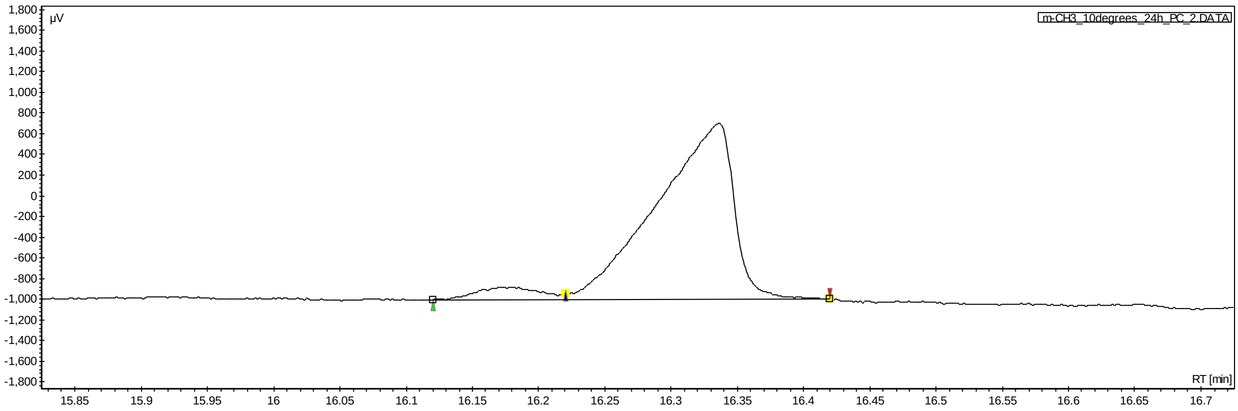

#	Name	Time [Min]	Quantity [% Area	Height [µV]	Area [µV.Min]	Area % [%]
1	UNKNOWN	16.18	5.57	110.9	6.8	5.574
2	UNKNOWN	16.34	94.43	1701.4	114.6	94.426
Total			100.00	1812.3	121.4	100.000

Sample prepared using catalyst **2** in propylene carbonate (Table 5, entry 7)

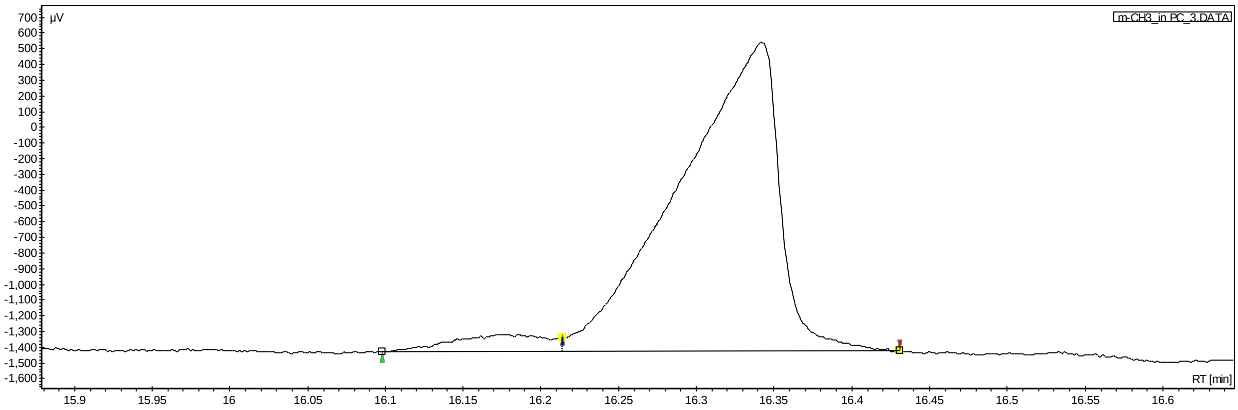

#	Name	Time [Min]	Quantity [% Area	Height [µV]	Area [µV.Min]	Area % [%]
1	UNKNOWN	16.18	4.85	102.6	7.5	4.850
2	UNKNOWN	16.34	95.15	1964.7	146.7	95.150
Total			100.00	2067.3	154.2	100.000

Cyanohydrin acetate derived from 4-methylbenzaldehyde

Analysed using GC method 3.

Racemate

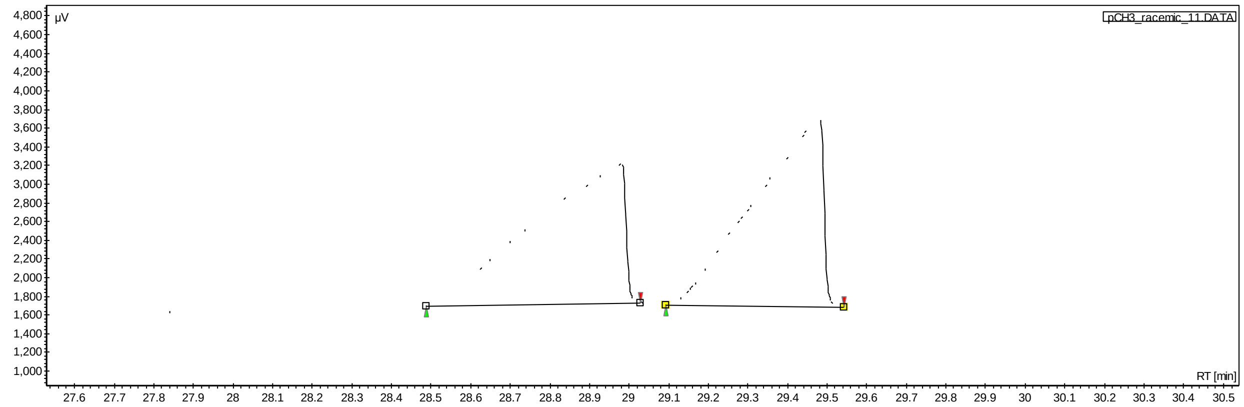

#	Name	Time [Min]	Quantity [% Are	Height [µV]	Area [µV.Min]	Area % [%]
1	UNKNOWN	28.98	49.68	1492.5	396.4	49.683
2	UNKNOWN	29.48	50.32	2011.9	401.5	50.317
Total			100.00	3504.4	797.9	100.000

Sample prepared using catalyst **1** in dichloromethane (Table 1)

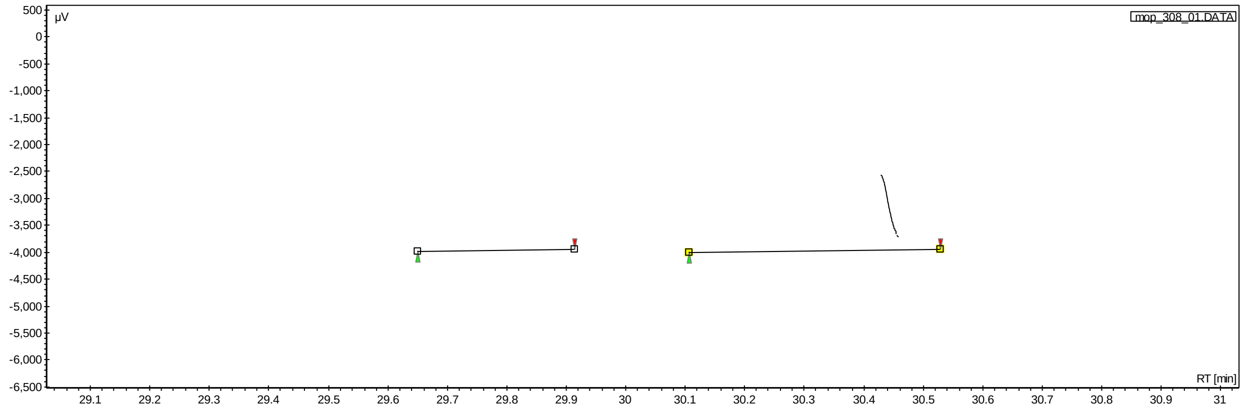

#	Name	Time [Min]	Quantity [% Are	Height [µV]	Area [µV.Min]	Area % [%]
1	UNKNOWN	29.82	15.79	329.8	47.5	15.790
2	UNKNOWN	30.43	84.21	1406.5	253.5	84.210
Total			100.00	1736.2	301.1	100.000

Sample prepared using catalyst **1** in propylene carbonate (Table 1)

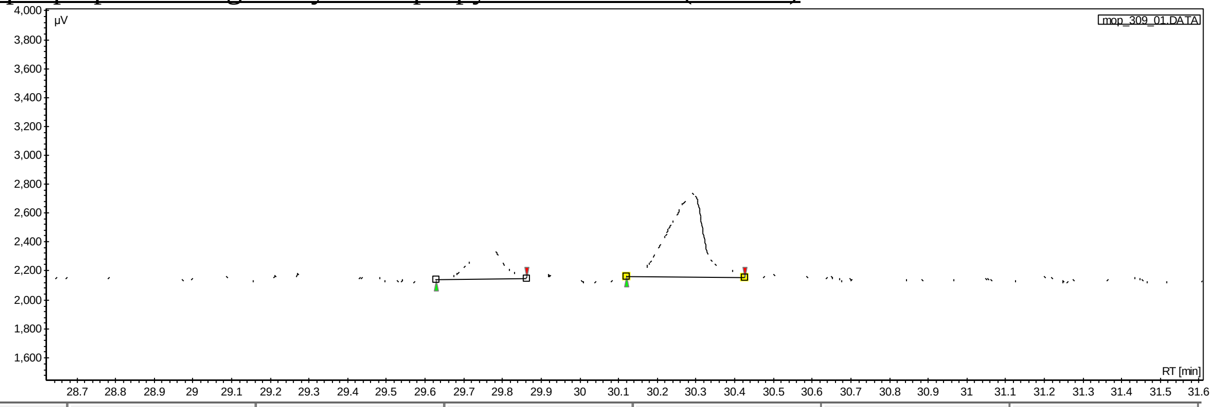

	#	Name	Time [Min]	Quantity [% Area]	Height [µV]	Area [µV.Min]	Area % [%]
	1	UNKNOWN	29.76	25.28	224.1	21.4	25.283
	2	UNKNOWN	30.29	74.72	579.2	63.3	74.717
▶							
	Total			100.00	803.3	84.8	100.000

Sample prepared using catalyst **2** in dichloromethane (Table 1)

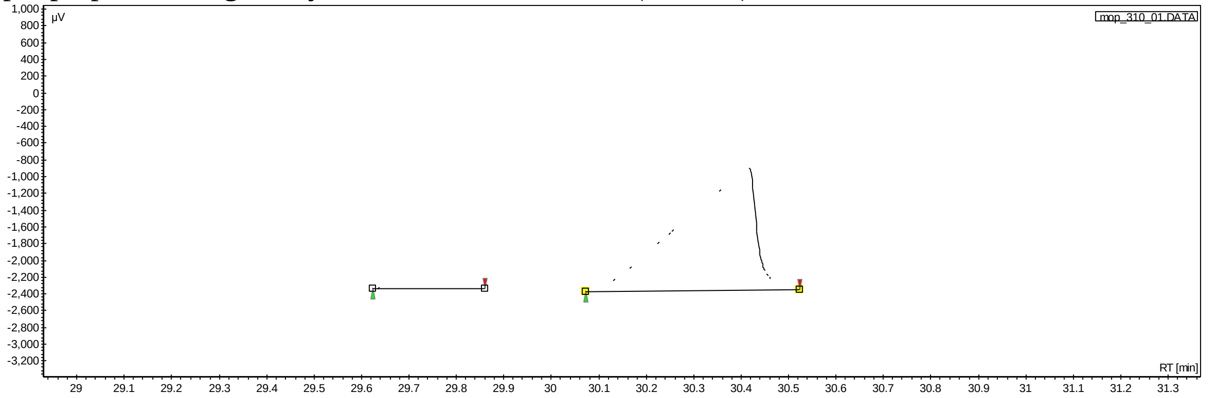

	#	Name	Time [Min]	Quantity [% Area]	Height [µV]	Area [µV.Min]	Area % [%]
	1	UNKNOWN	29.77	6.39	140.4	18.1	6.389
	2	UNKNOWN	30.42	93.61	1465.6	265.1	93.611
▶							
	Total			100.00	1606.0	283.2	100.000

Sample prepared using catalyst **2** in propylene carbonate (Table 1)

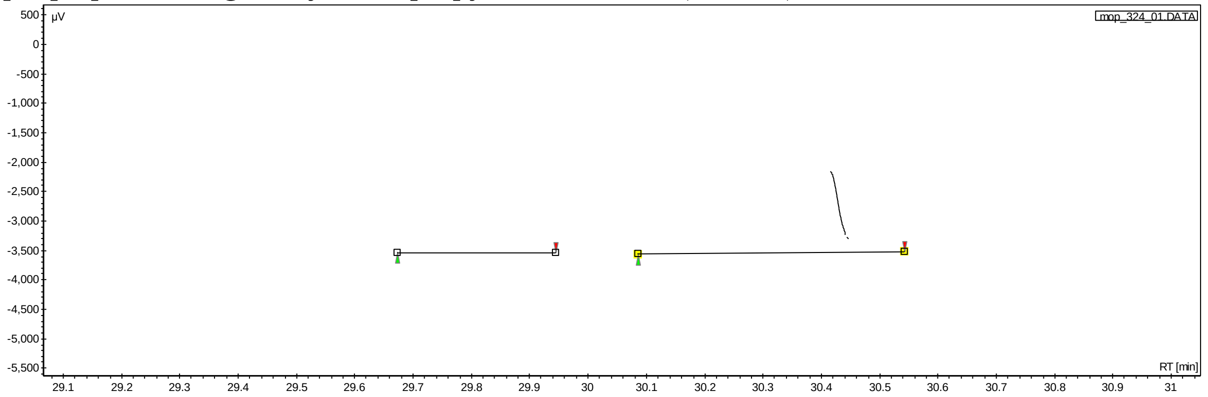

	#	Name	Time [Min]	Quantity [% Area]	Height [µV]	Area [µV.Min]	Area % [%]
	2	UNKNOWN	29.78	6.87	140.1	18.2	6.874
	1	UNKNOWN	30.41	93.13	1396.2	246.3	93.126
▶							
	Total			100.00	1536.4	264.5	100.000

Sample prepared using catalyst **2** in propylene carbonate (Table 2, entry 8)

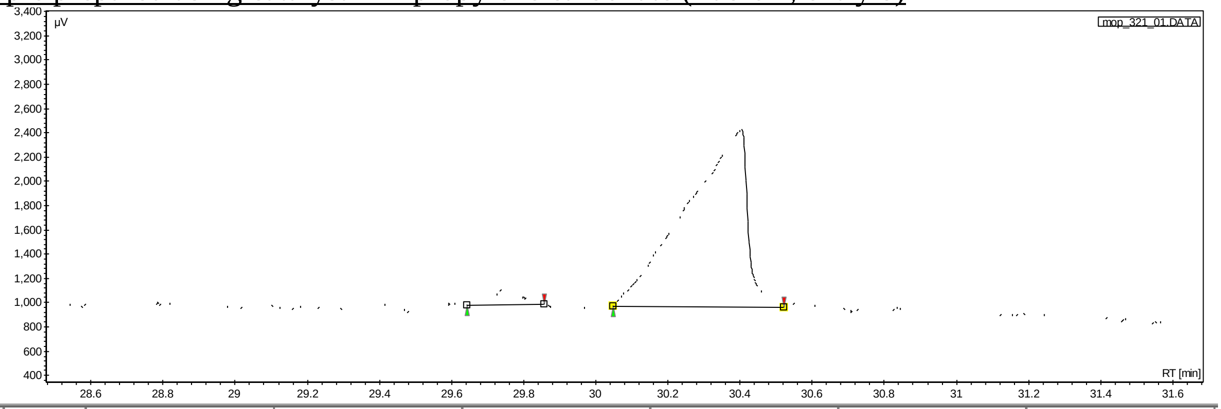

	#	Name	Time [Min]	Quantity [% Area	Height [μV]	Area [μV.Min]	Area % [%]
	2	UNKNOWN	29.74	4.89	133.2	14.8	4.891
	1	UNKNOWN	30.40	95.11	1462.1	288.4	95.109
▶							
	Total			100.00	1595.3	303.2	100.000

Sample prepared using catalyst **2** in propylene carbonate (Table 2, entry 11)

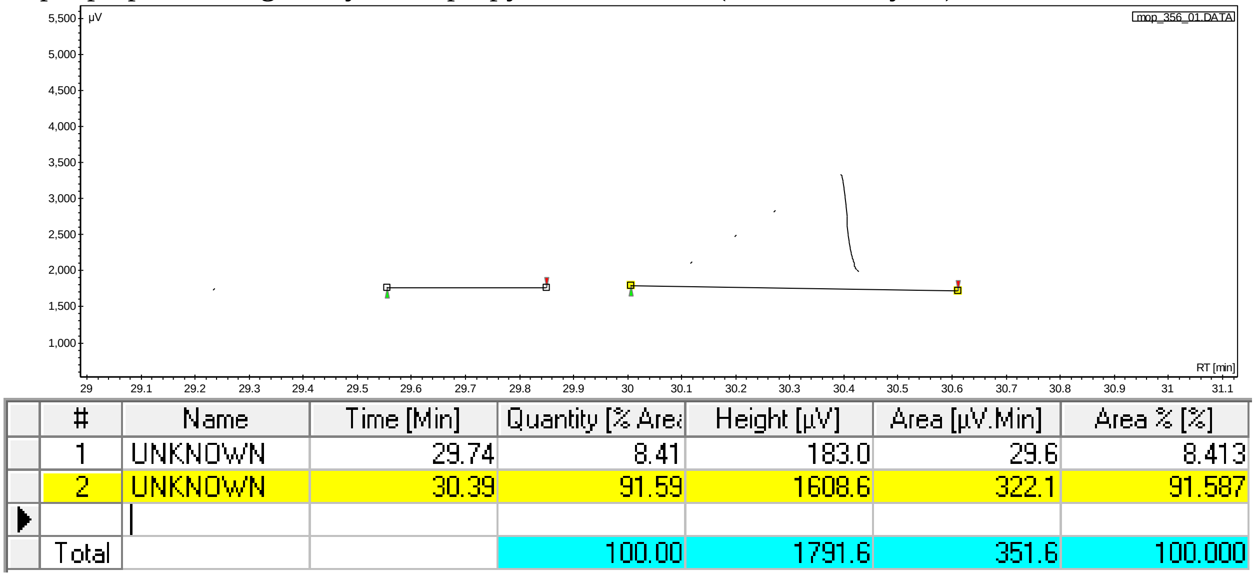

	#	Name	Time [Min]	Quantity [% Area	Height [μV]	Area [μV.Min]	Area % [%]
	1	UNKNOWN	29.74	8.41	183.0	29.6	8.413
	2	UNKNOWN	30.39	91.59	1608.6	322.1	91.587
▶							
	Total			100.00	1791.6	351.6	100.000

Sample prepared using catalyst **2** in propylene carbonate (Table 5, entry 4)

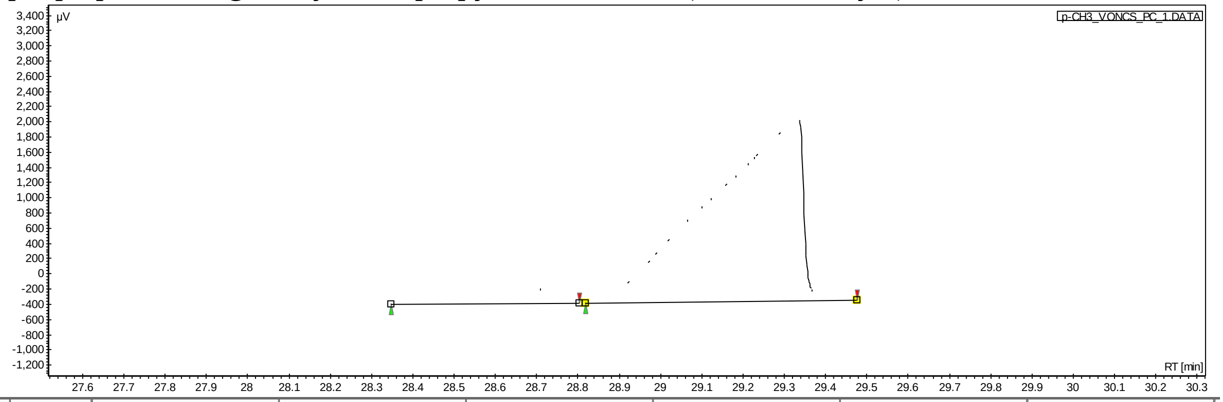

	#	Name	Time [Min]	Quantity [% Area	Height [μV]	Area [μV.Min]	Area % [%]
	1	UNKNOWN	28.59	11.45	291.5	79.7	11.453
	2	UNKNOWN	29.34	88.55	2378.0	616.0	88.547
▶							
	Total			100.00	2669.5	695.7	100.000

Cyanohydrin acetate derived from nonanal

Analysed using GC method 5.

Racemate

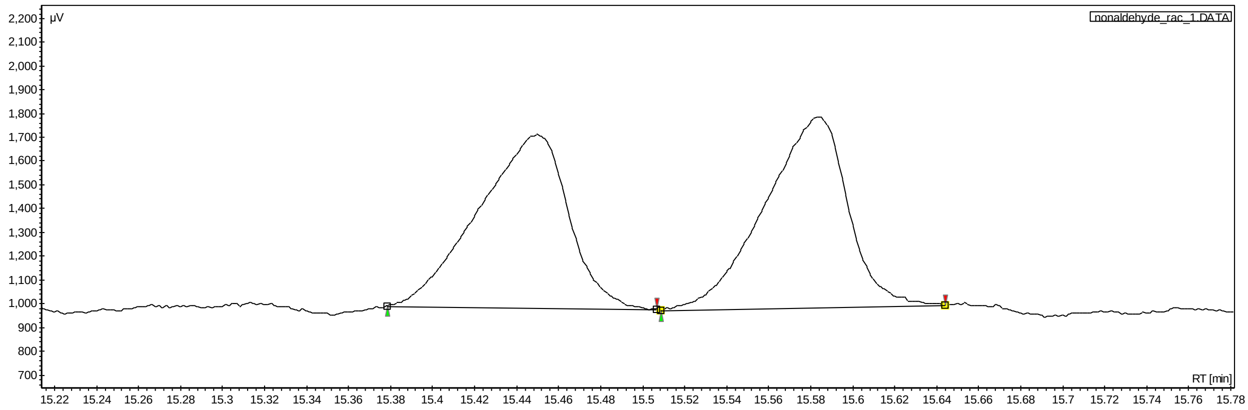

#	Name	Time [Min]	Quantity [% Are	Height [µV]	Area [µV.Min]	Area % [%]
1	UNKNOWN	15.45	49.55	731.8	36.4	49.551
2	UNKNOWN	15.58	50.45	803.7	37.1	50.449
Total			100.00	1535.5	73.5	100.000

Sample prepared using catalyst **1** in dichloromethane (Table 1)

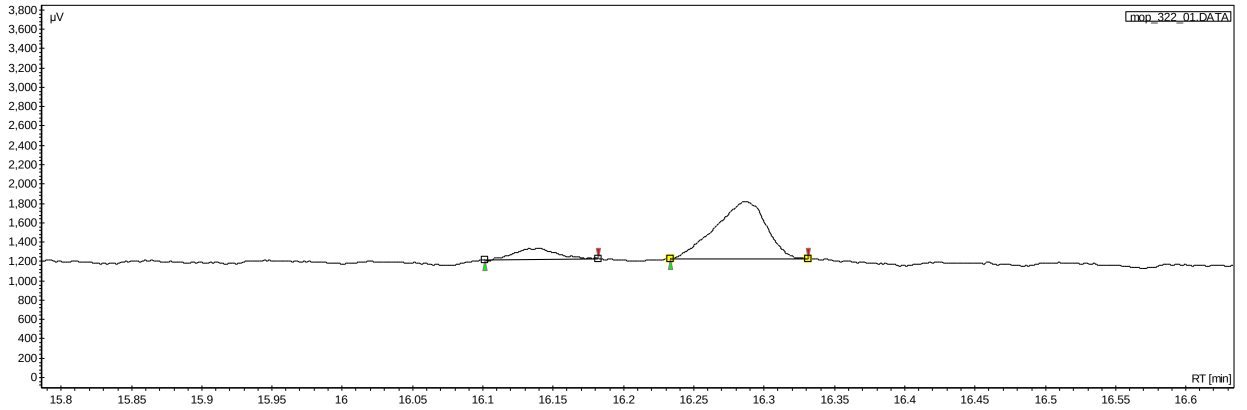

#	Name	Time [Min]	Quantity [% Are	Height [µV]	Area [µV.Min]	Area % [%]
1	UNKNOWN	16.14	13.50	106.5	3.9	13.505
2	UNKNOWN	16.29	86.50	586.6	25.1	86.495
Total			100.00	693.1	29.0	100.000

Sample prepared using catalyst **1** in propylene carbonate (Table 1)

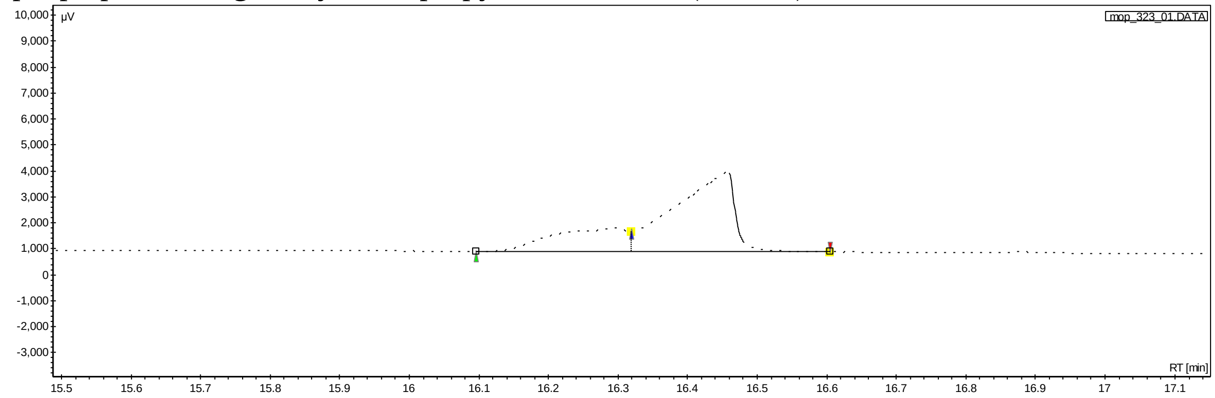

	#	Name	Time [Min]	Quantity [% Area	Height [µV]	Area [µV.Min]	Area % [%]
	1	UNKNOWN	16.30	27.52	920.2	115.2	27.516
	2	UNKNOWN	16.46	72.48	3072.9	303.4	72.484
▶							
	Total			100.00	3993.1	418.6	100.000

Sample prepared using catalyst **2** in dichloromethane (Table 1)

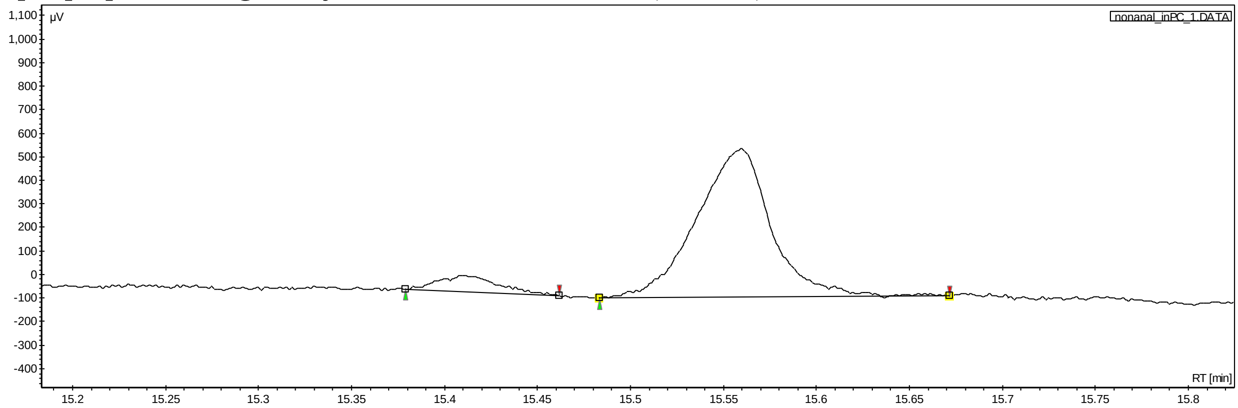

	#	Name	Time [Min]	Quantity [% Area	Height [µV]	Area [µV.Min]	Area % [%]
	2	UNKNOWN	15.41	8.43	67.9	2.7	8.429
	1	UNKNOWN	15.56	91.57	618.6	29.0	91.571
▶							
	Total			100.00	686.5	31.6	100.000

Sample prepared using catalyst **2** in propylene carbonate (Table 1)

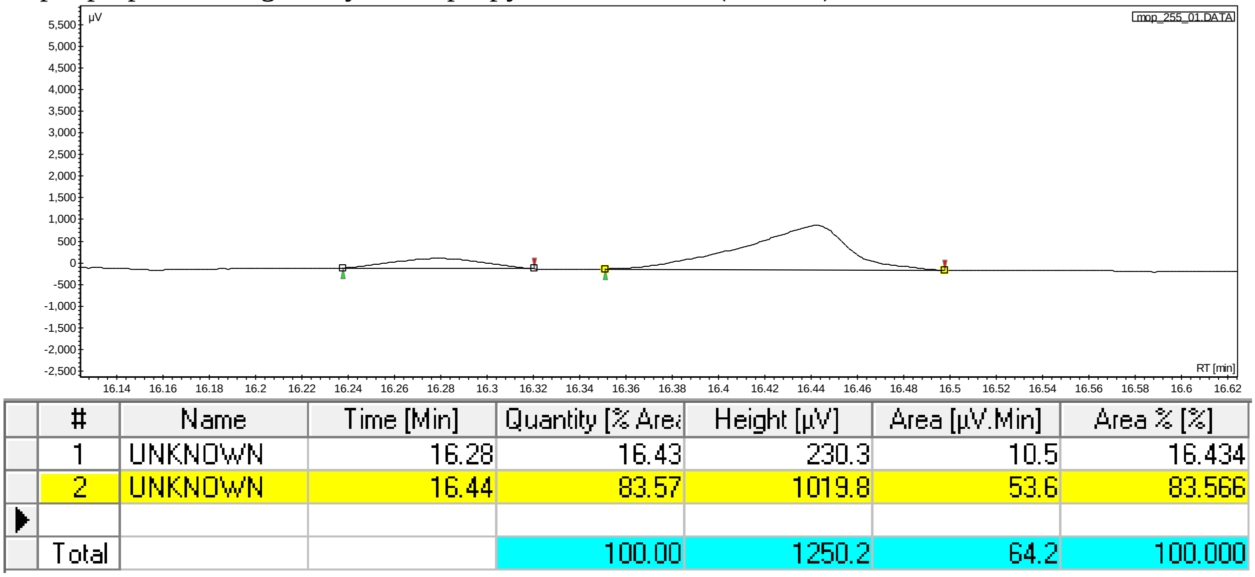

	#	Name	Time [Min]	Quantity [% Area	Height [µV]	Area [µV.Min]	Area % [%]
	1	UNKNOWN	16.28	16.43	230.3	10.5	16.434
	2	UNKNOWN	16.44	83.57	1019.8	53.6	83.566
▶							
	Total			100.00	1250.2	64.2	100.000

Sample prepared using catalyst **2** in propylene carbonate (Table 2, entry 12)

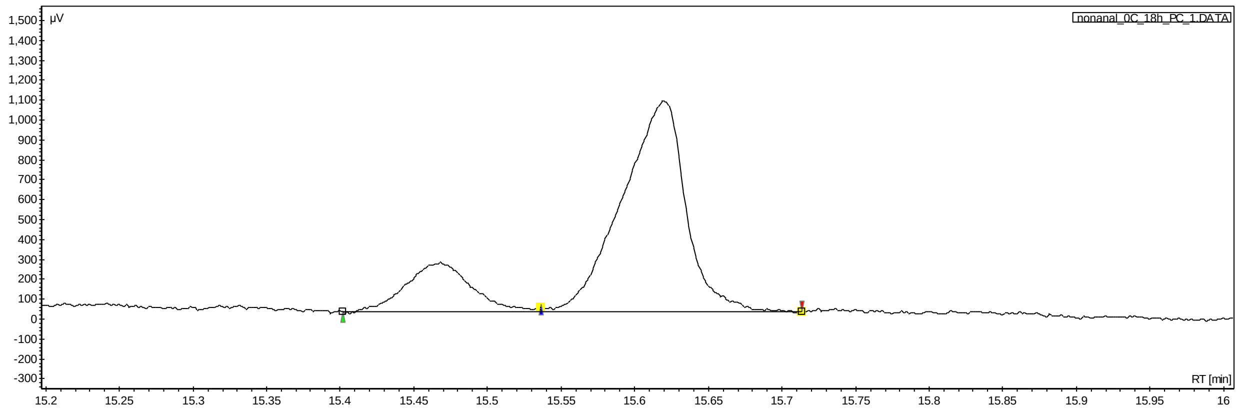

	#	Name	Time [Min]	Quantity [% Are	Height [µV]	Area [µV.Min]	Area % [%]
	1	UNKNOWN	15.47	19.46	244.2	13.0	19.460
	2	UNKNOWN	15.62	80.54	1059.8	53.6	80.540
▶							
	Total			100.00	1304.0	66.6	100.000

Sample prepared using catalyst **2** in propylene carbonate (Table 2, entry 15)

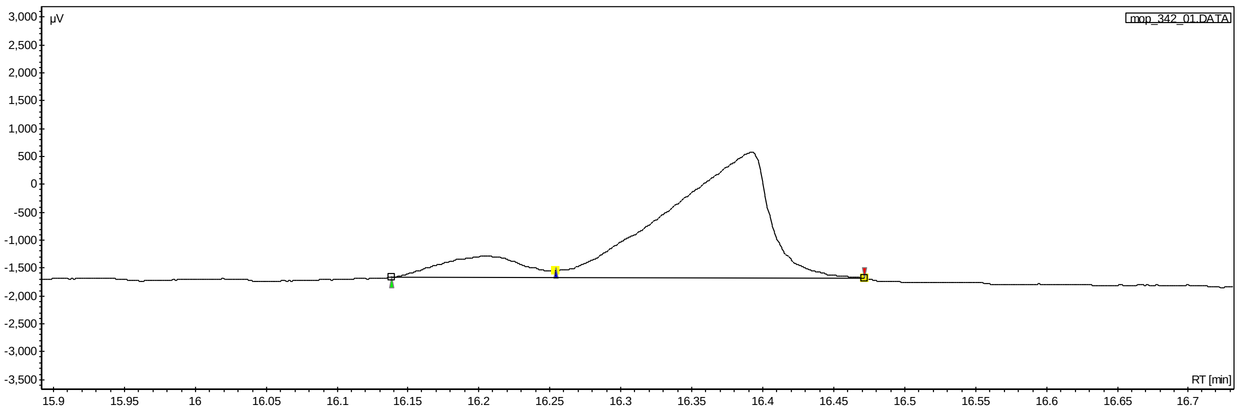

	#	Name	Time [Min]	Quantity [% Are	Height [µV]	Area [µV.Min]	Area % [%]
	1	UNKNOWN	16.21	12.39	378.5	26.2	12.391
	2	UNKNOWN	16.39	87.61	2259.4	185.4	87.609
▶							
	Total			100.00	2637.9	211.7	100.000

Cyanohydrin acetate derived from pivaldehyde

Analysed using GC method 5.

<u>Racemate</u>

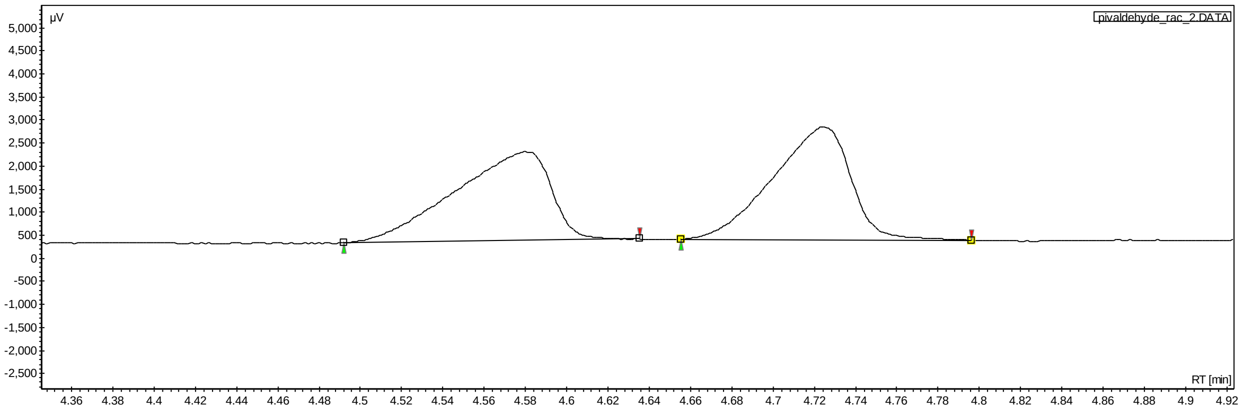

	#	Name	Time [Min]	Quantity [% Are	Height [µV]	Area [µV.Min]	Area % [%]
	2	UNKNOWN	4.58	49.16	1917.7	103.2	49.157
	1	UNKNOWN	4.73	50.84	2449.9	106.7	50.843
▶							
	Total			100.00	4367.6	209.9	100.000

<u>Sample prepared using catalyst **1** in dichloromethane (Table 1)</u>

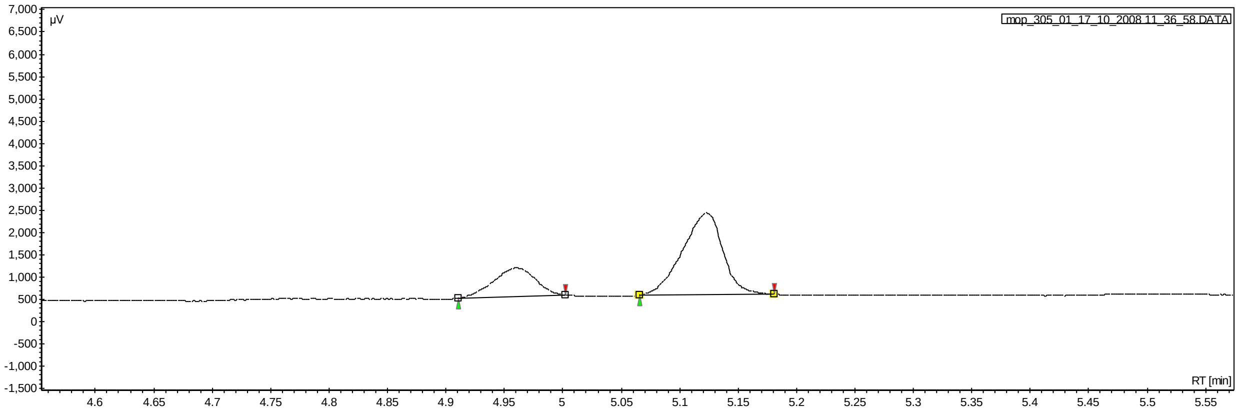

	#	Name	Time [Min]	Quantity [% Are	Height [µV]	Area [µV.Min]	Area % [%]
	1	UNKNOWN	4.96	26.57	647.0	26.8	26.570
	2	UNKNOWN	5.12	73.43	1843.8	74.0	73.430
▶							
	Total			100.00	2490.8	100.7	100.000

Sample prepared using catalyst **1** in propylene carbonate (Table 1)

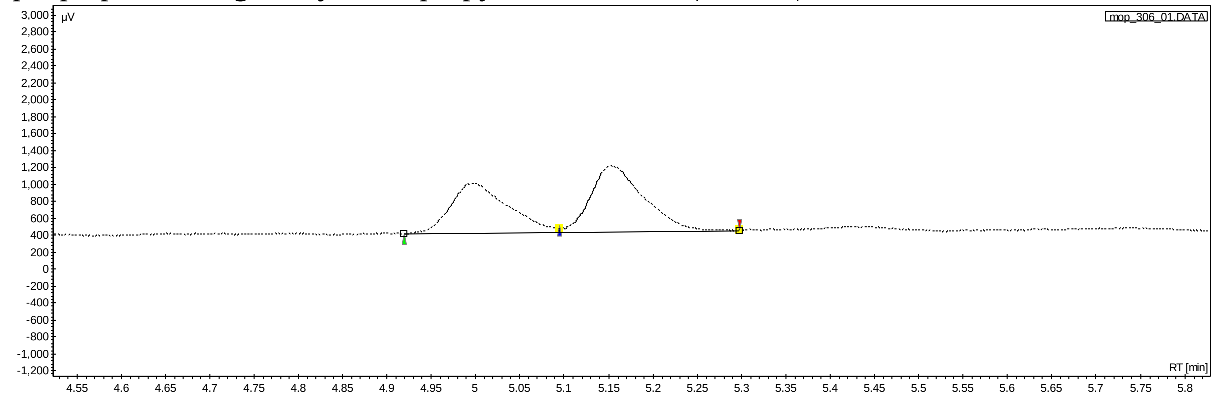

#	Name	Time [Min]	Quantity [% Area	Height [μV]	Area [μV.Min]	Area % [%]
1	UNKNOWN	5.00	44.94	584.9	44.0	44.941
2	UNKNOWN	5.15	55.06	784.5	53.9	55.059
Total			100.00	1369.3	98.0	100.000

Sample prepared using catalyst **2** in dichloromethane (Table 1)

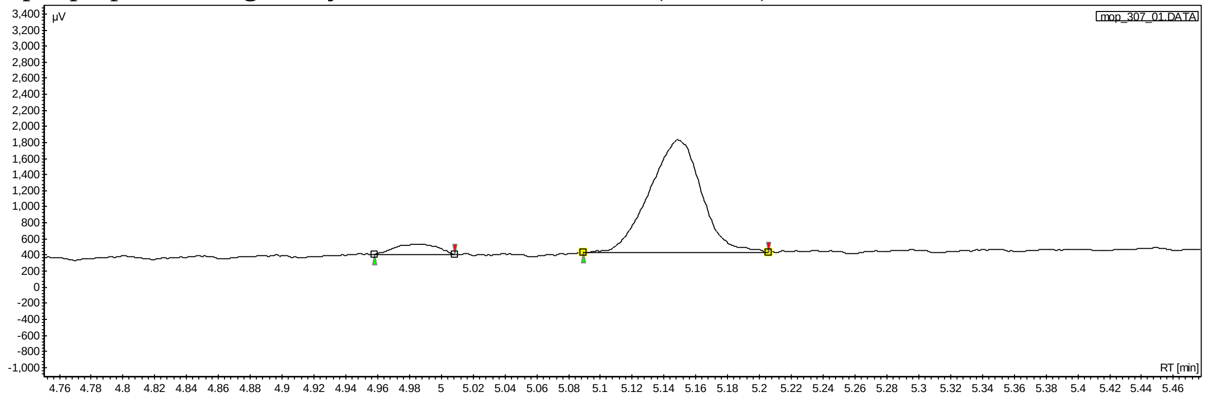

#	Name	Time [Min]	Quantity [% Area	Height [μV]	Area [μV.Min]	Area % [%]
1	UNKNOWN	4.99	6.99	123.9	4.0	6.992
2	UNKNOWN	5.15	93.01	1401.9	53.0	93.008
Total			100.00	1525.8	57.0	100.000

Sample prepared using catalyst **2** in propylene carbonate (Table 1)

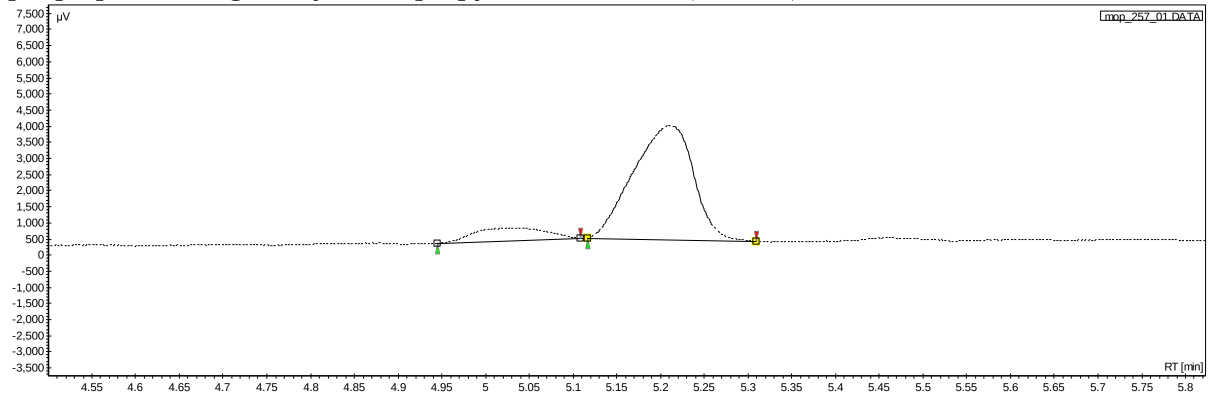

#	Name	Time [Min]	Quantity [% Area	Height [μV]	Area [μV.Min]	Area % [%]
1	UNKNOWN	5.03	12.12	408.2	38.9	12.115
2	UNKNOWN	5.21	87.88	3544.5	281.9	87.885
Total			100.00	3952.7	320.8	100.000

Sample prepared using catalyst **2** in propylene carbonate (Table 2, entry 14)

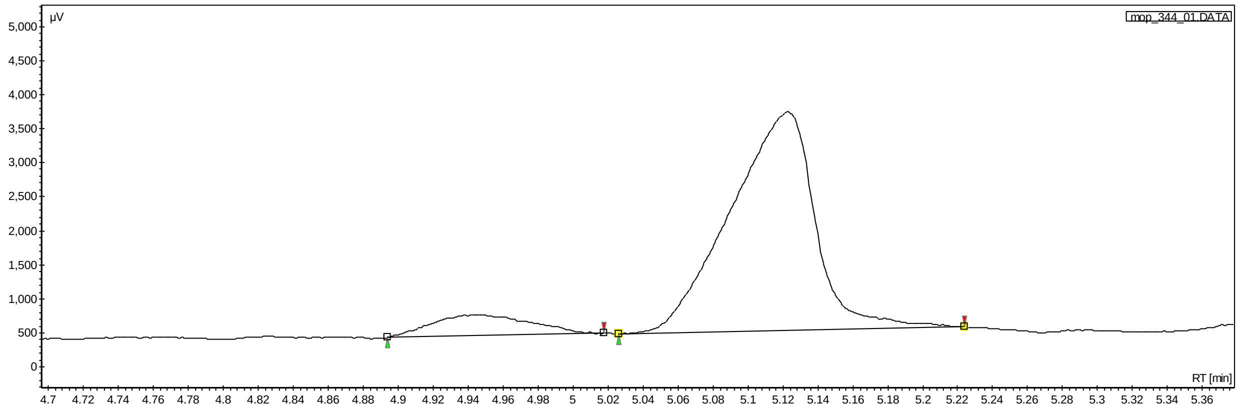

	#	Name	Time [Min]	Quantity [% Area]	Height [µV]	Area [µV.Min]	Area % [%]
	1	UNKNOWN	4.95	10.07	300.6	20.3	10.065
	2	UNKNOWN	5.12	89.93	3217.5	181.3	89.935
▶							
	Total			100.00	3518.2	201.6	100.000

Sample prepared using catalyst **2** in propylene carbonate (Table 2, entry 17)

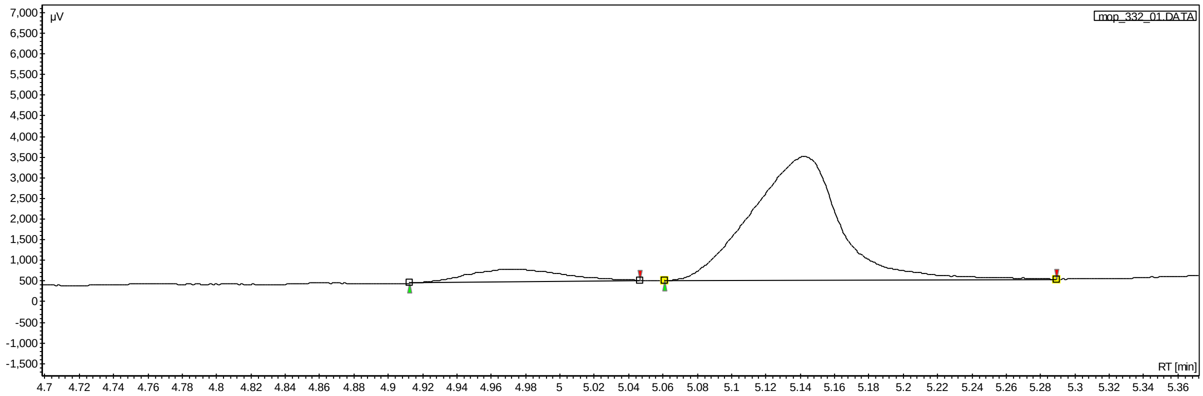

	#	Name	Time [Min]	Quantity [% Area]	Height [µV]	Area [µV.Min]	Area % [%]
	1	UNKNOWN	4.97	9.95	305.6	19.9	9.952
	2	UNKNOWN	5.14	90.05	2994.2	180.4	90.048
▶							
	Total			100.00	3299.8	200.4	100.000

Cyanohydrin acetate derived from cyclohexane carboxaldehyde

Analysed using GC method 5.

Racemate

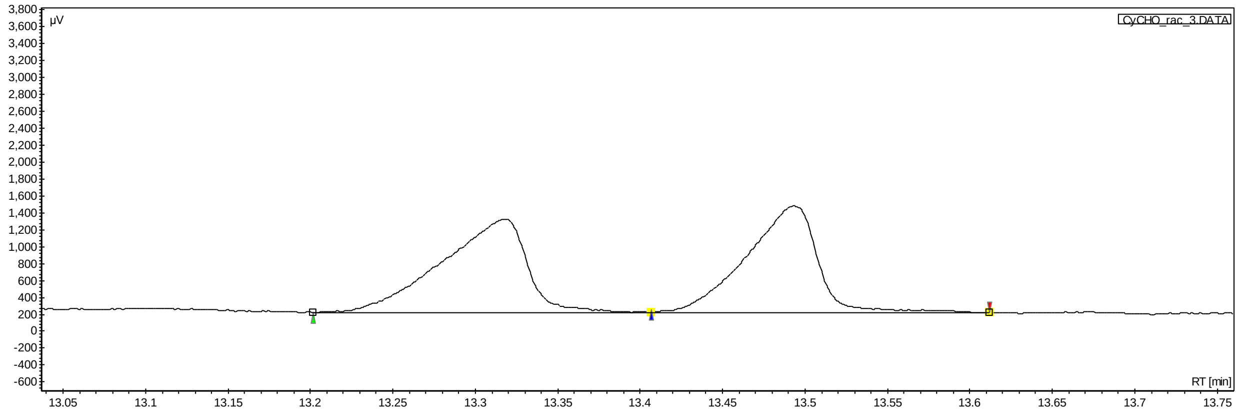

	#	Name	Time [Min]	Quantity [% Are	Height [μV]	Area [μV.Min]	Area % [%]
	1	UNKNOWN	13.32	50.93	1104.2	65.8	50.929
	2	UNKNOWN	13.49	49.07	1263.5	63.4	49.071
▶							
	Total			100.00	2367.7	129.2	100.000

Sample prepared using catalyst **1** in dichloromethane (Table 1)

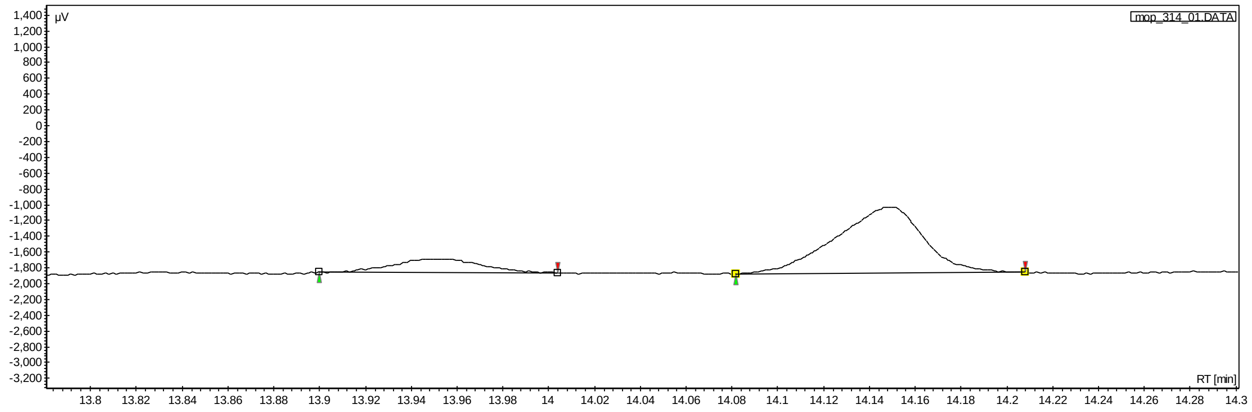

	#	Name	Time [Min]	Quantity [% Are	Height [μV]	Area [μV.Min]	Area % [%]
	1	UNKNOWN	13.95	17.04	174.2	7.6	17.035
	2	UNKNOWN	14.15	82.96	831.7	36.9	82.965
▶							
	Total			100.00	1005.9	44.5	100.000

Sample prepared using catalyst **1** in propylene carbonate (Table 1)

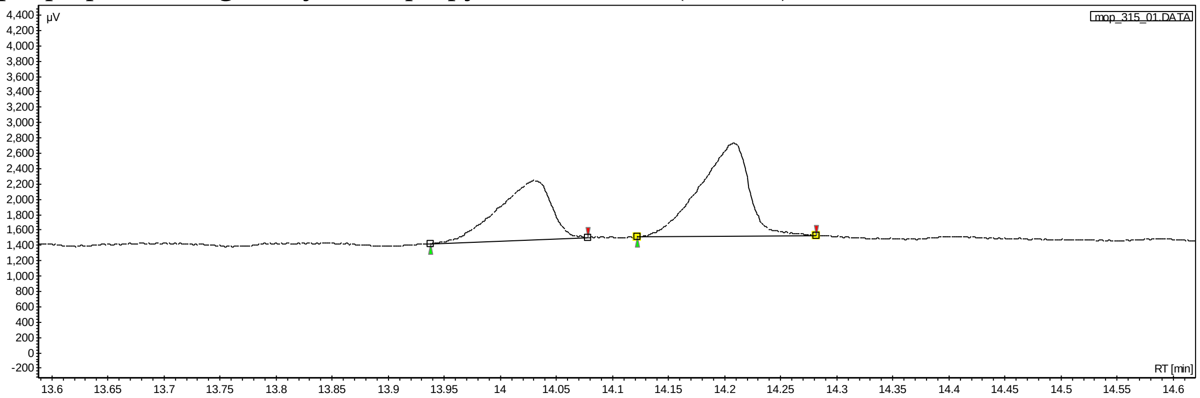

#	Name	Time [Min]	Quantity [% Area	Height [μV]	Area [μV.Min]	Area % [%]
1	UNKNOWN	14.03	40.57	772.1	42.4	40.569
2	UNKNOWN	14.21	59.43	1210.0	62.2	59.431
Total			100.00	1982.1	104.6	100.000

Sample prepared using catalyst **2** in dichloromethane (Table 1)

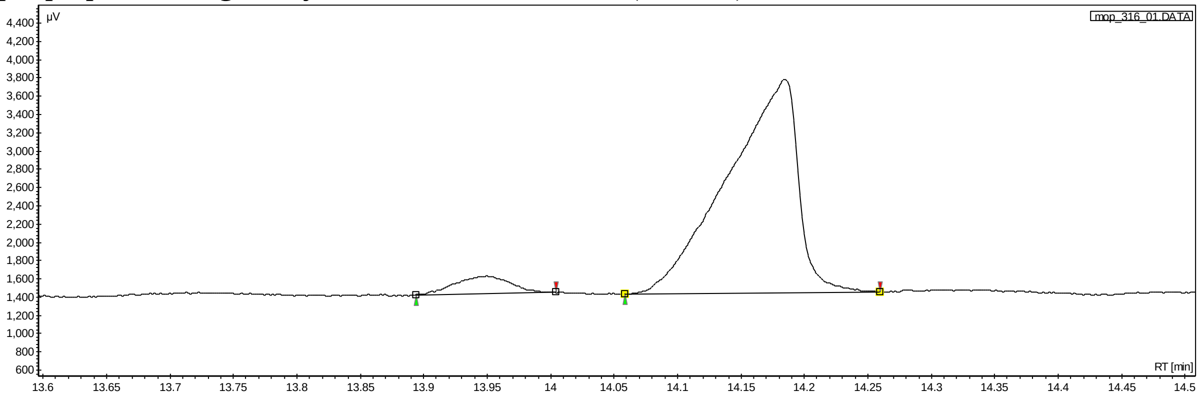

#	Name	Time [Min]	Quantity [% Area	Height [μV]	Area [μV.Min]	Area % [%]
2	UNKNOWN	13.95	6.11	184.4	9.9	6.114
1	UNKNOWN	14.19	93.89	2337.8	151.8	93.886
Total			100.00	2522.2	161.7	100.000

Sample prepared using catalyst **2** in propylene carbonate (Table 1)

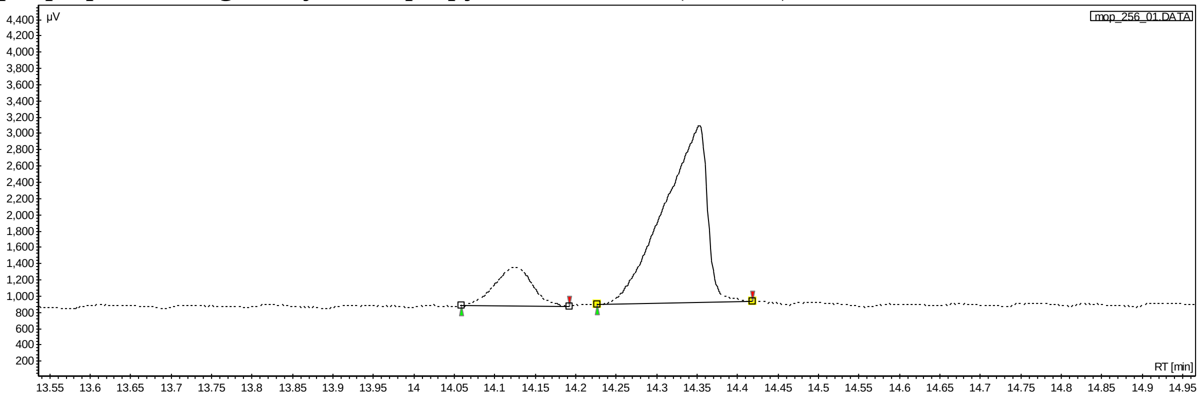

#	Name	Time [Min]	Quantity [% Area	Height [μV]	Area [μV.Min]	Area % [%]
1	UNKNOWN	14.13	16.43	481.9	26.4	16.427
2	UNKNOWN	14.35	83.57	2169.3	134.4	83.573
Total			100.00	2651.1	160.8	100.000

Sample prepared using catalyst **2** in propylene carbonate (Table 2, entry 13)

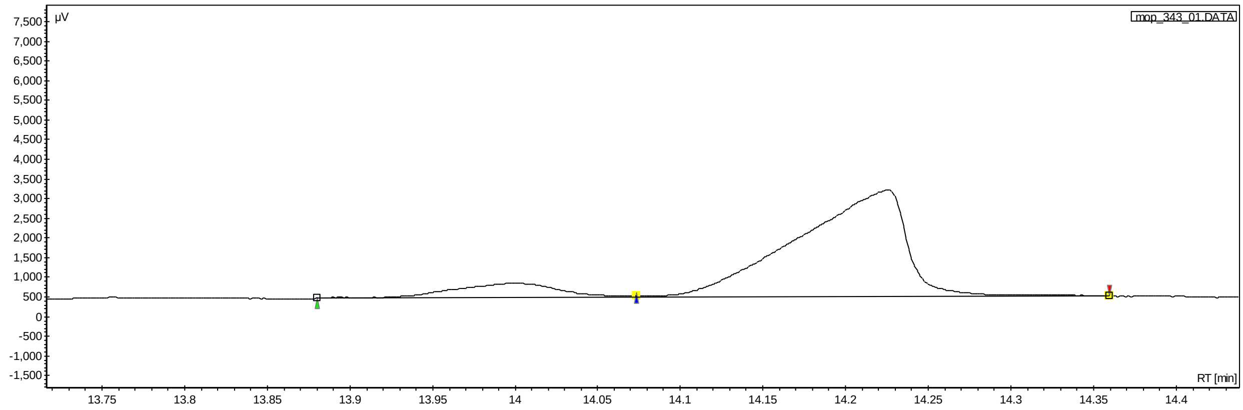

#	Name	Time [Min]	Quantity [% Area]	Height [µV]	Area [µV.Min]	Area % [%]
1	UNKNOWN	14.00	11.98	367.8	28.6	11.983
2	UNKNOWN	14.23	88.02	2718.8	210.0	88.017
Total			100.00	3086.6	238.6	100.000

Sample prepared using catalyst **2** in propylene carbonate (Table 2, entry 16)

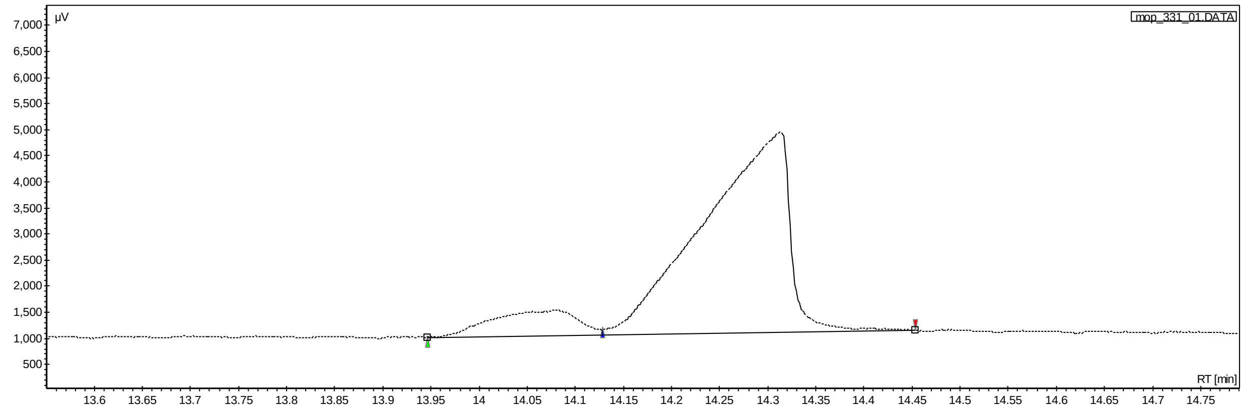

#	Name	Time [Min]	Quantity [% Area]	Height [µV]	Area [µV.Min]	Area % [%]
1	UNKNOWN	14.08	11.27	480.2	50.6	11.274
2	UNKNOWN	14.31	88.73	3842.0	398.1	88.726
Total			100.00	4322.2	448.7	100.000

Cyanohydrin acetate derived from 3-fluorobenzaldehyde

Analysed using GC method 3.

<u>Racemate</u>

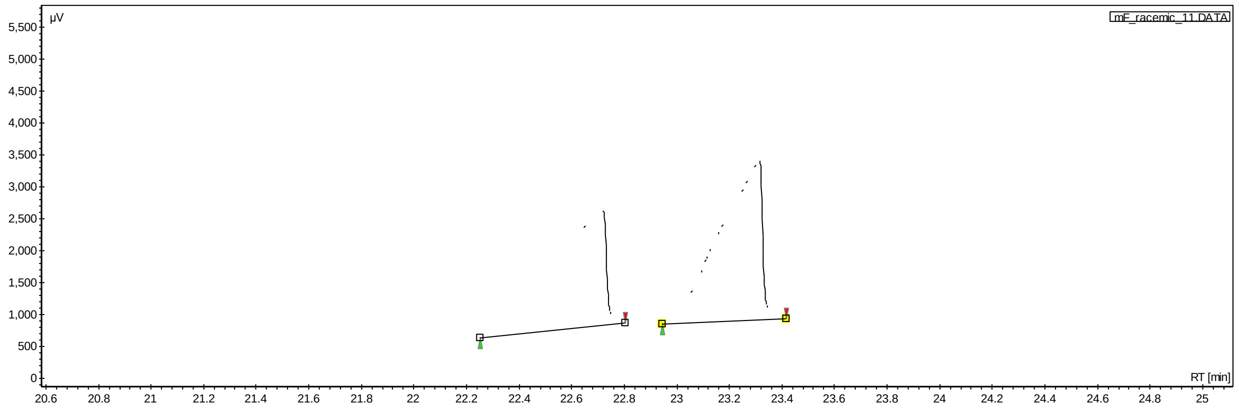

	#	Name	Time [Min]	Quantity [% Are	Height [µV]	Area [µV.Min]	Area % [%]
	1	UNKNOWN	22.71	50.14	1828.7	462.9	50.142
	2	UNKNOWN	23.31	49.86	2496.6	460.3	49.858
	Total			100.00	4325.3	923.1	100.000

<u>Sample prepared using catalyst **2** in propylene carbonate (Table 5, entry 6)</u>

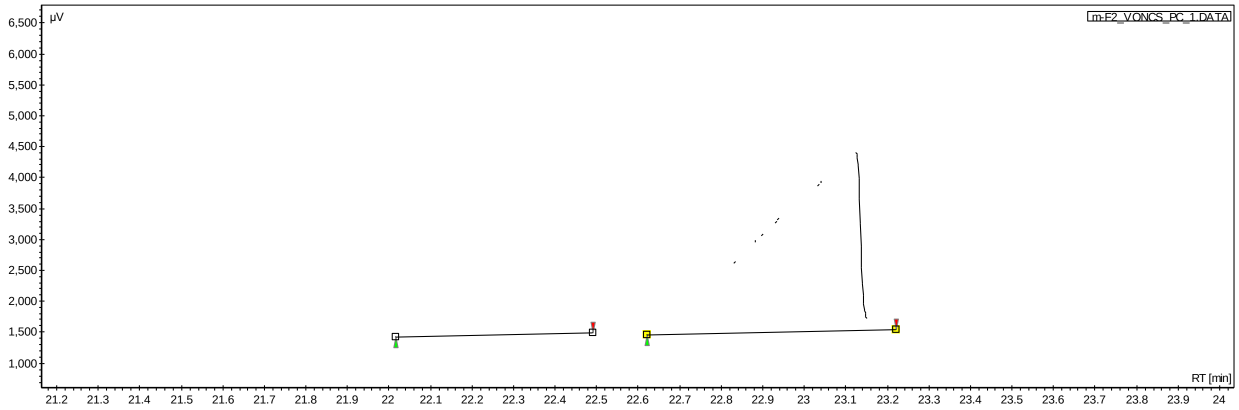

	#	Name	Time [Min]	Quantity [% Are	Height [µV]	Area [µV.Min]	Area % [%]
	1	UNKNOWN	22.30	14.06	413.6	121.0	14.060
	2	UNKNOWN	23.12	85.94	2860.7	739.5	85.940
	Total			100.00	3274.2	860.5	100.000

Cyanohydrin acetate derived from 4-trifluoromethylbenzaldehyde

Analysed using GC method 2.

<u>Racemate</u>

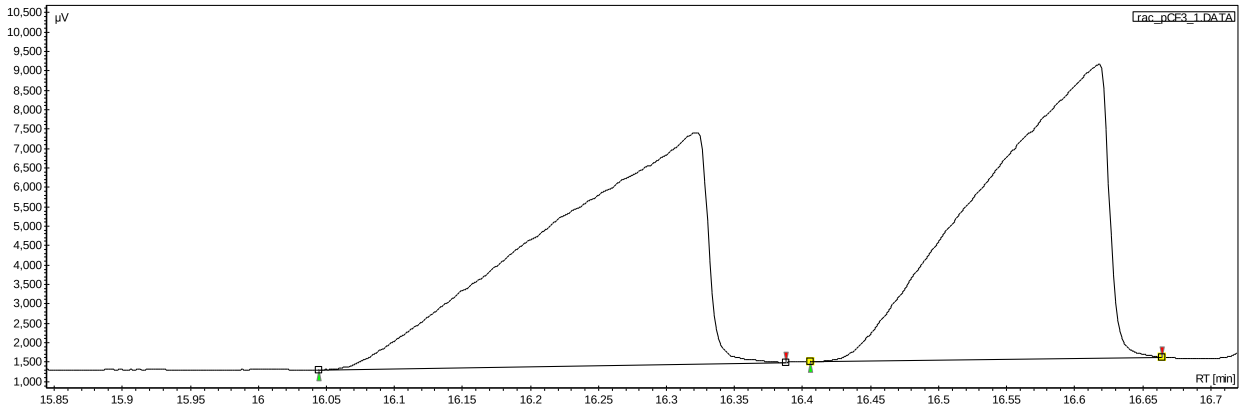

#	Name	Time [Min]	Quantity [% Are	Height [µV]	Area [µV.Min]	Area % [%]
1	UNKNOWN	16.32	50.65	5947.7	834.4	50.650
2	UNKNOWN	16.62	49.35	7563.4	813.0	49.350
Total			100.00	13511.1	1647.4	100.000

<u>Sample prepared using catalyst **2** in propylene carbonate (Table 5, entry 8)</u>

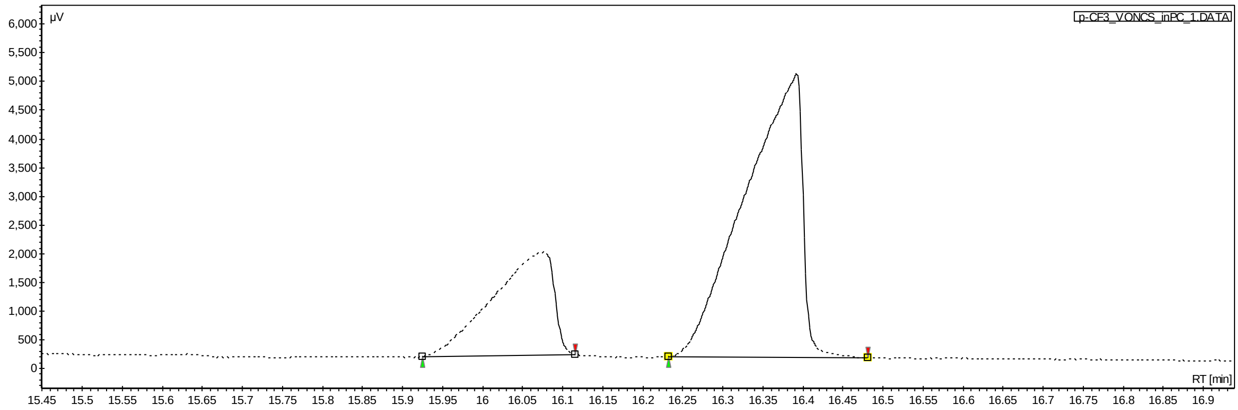

#	Name	Time [Min]	Quantity [% Are	Height [µV]	Area [µV.Min]	Area % [%]
1	UNKNOWN	16.08	27.83	1795.2	158.9	27.830
2	UNKNOWN	16.39	72.17	4931.2	412.1	72.170
Total			100.00	6726.4	571.0	100.000

Cyanohydrin acetate derived from 4-bromobenzaldehyde

Analysed using GC method 3.

Racemate

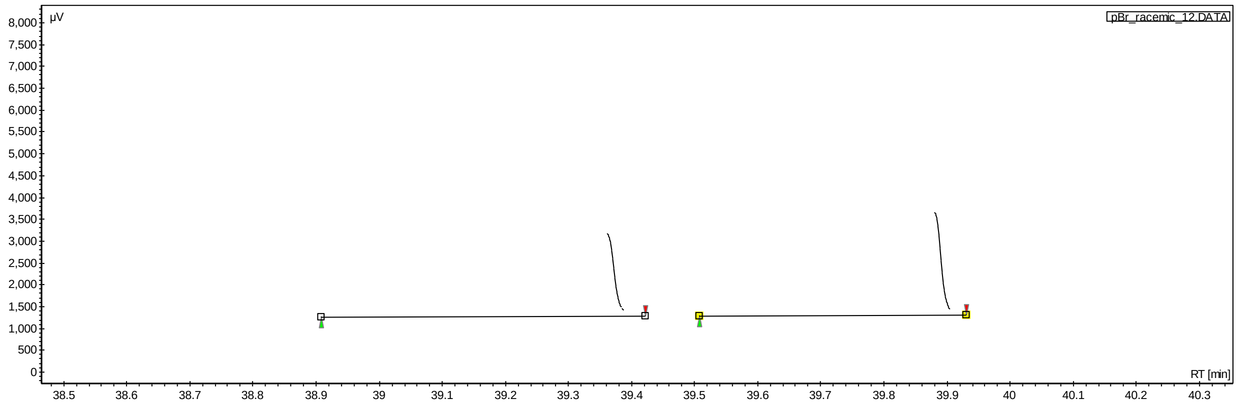

	#	Name	Time [Min]	Quantity [% Area]	Height [µV]	Area [µV.Min]	Area % [%]
	1	UNKNOWN	39.36	50.34	1901.8	444.3	50.336
	2	UNKNOWN	39.88	49.66	2365.6	438.4	49.664
▶							
	Total			100.00	4267.4	882.7	100.000

Sample prepared using catalyst **2** in propylene carbonate (Table 5, entry 9)

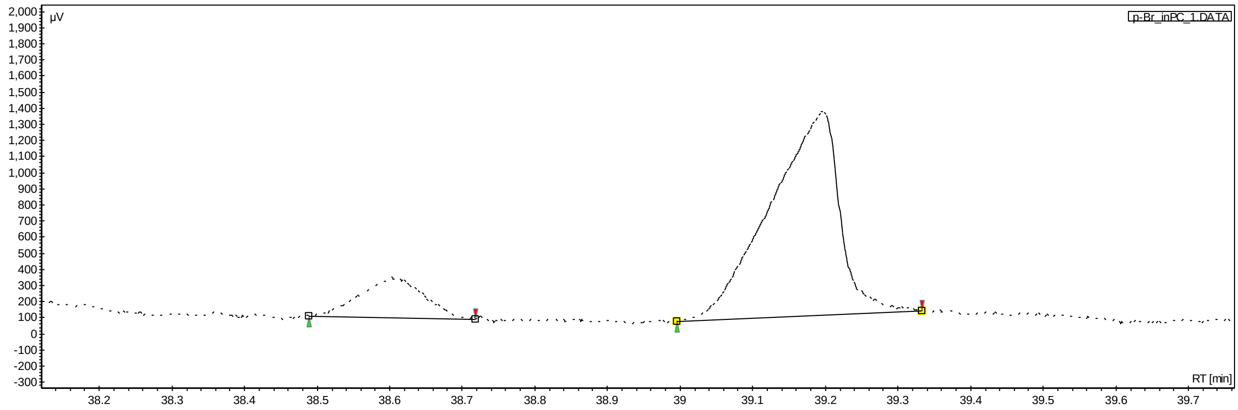

	#	Name	Time [Min]	Quantity [% Area]	Height [µV]	Area [µV.Min]	Area % [%]
	1	UNKNOWN	38.61	14.93	239.3	24.8	14.934
	2	UNKNOWN	39.20	85.07	1265.6	141.5	85.066
▶							
	Total			100.00	1504.9	166.3	100.000

Cyanohydrin acetate derived from 3,5-difluorobenzaldehyde

Analysed using GC method 2.

<u>Racemate</u>

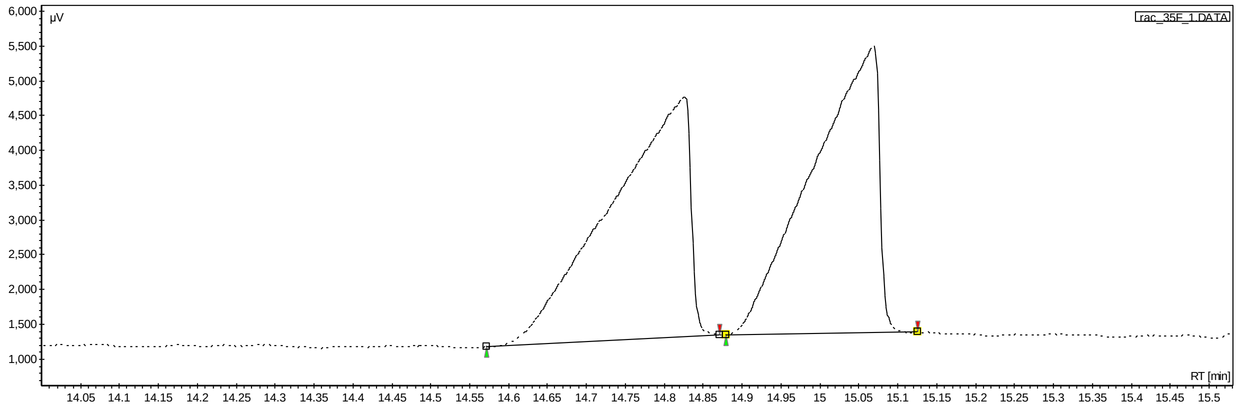

#	Name	Time [Min]	Quantity [% Area]	Height [µV]	Area [µV.Min]	Area % [%]
1	UNKNOWN	14.83	50.11	3452.5	412.8	50.112
2	UNKNOWN	15.07	49.89	4128.0	410.9	49.888
Total			100.00	7580.5	823.7	100.000

<u>Sample prepared using catalyst **2** in propylene carbonate (Table 5, entry 10)</u>

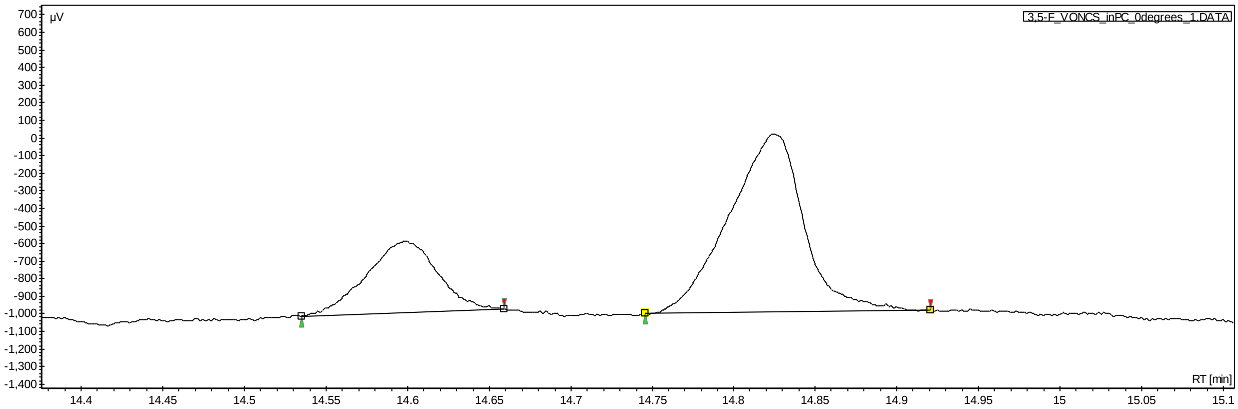

#	Name	Time [Min]	Quantity [% Area]	Height [µV]	Area [µV.Min]	Area % [%]
1	UNKNOWN	14.60	27.55	403.6	20.4	27.549
2	UNKNOWN	14.83	72.45	1014.1	53.7	72.451
Total			100.00	1417.7	74.2	100.000

HPLC of methyl mandelate

The analysis was performed on a Chirasil OD column using 80% hexane, 20% propan-2-ol as eluent with a flow rate of 1 mL per minute.

Racemate

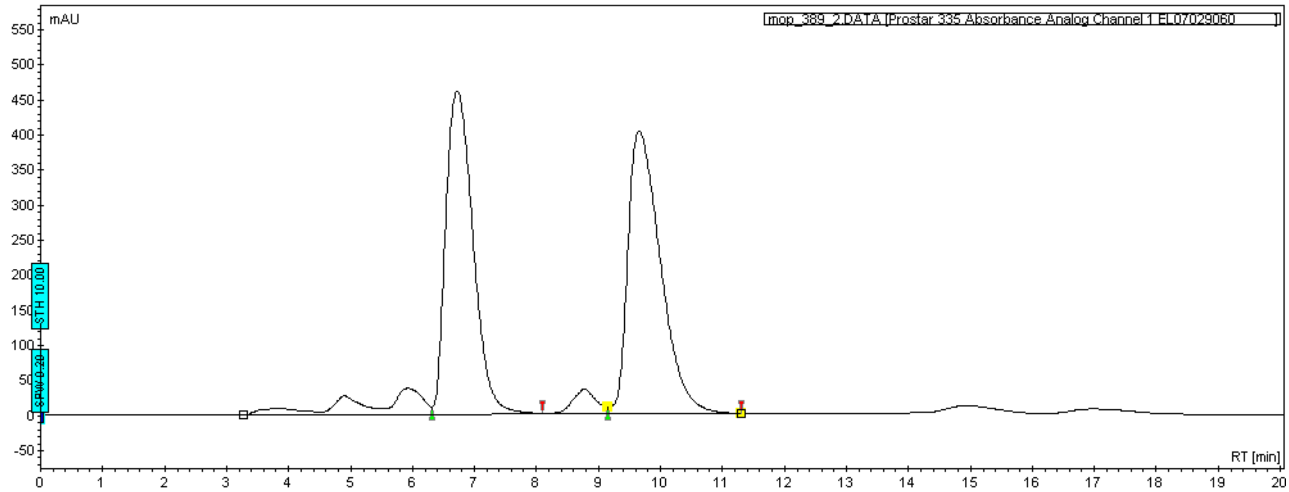

	#	Name	Time [Min]	Quantity [% Area	Height [mAU]	Area [mAU.Min]	Area % [%]
	1	UNKNOWN	6.72	48.90	460.4	235.4	48.899
	2	UNKNOWN	9.65	51.10	402.7	246.0	51.101
▶							
	Total			100.00	863.1	481.4	100.000

Sample with 81% ee

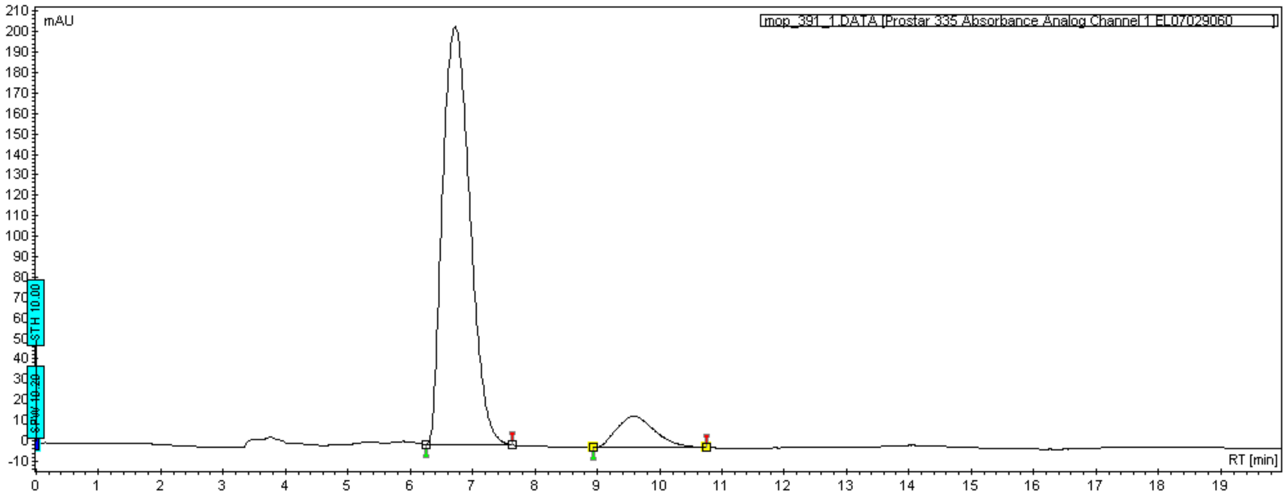

	#	Name	Time [Min]	Quantity [% Area	Height [mAU]	Area [mAU.Min]	Area % [%]
	2	UNKNOWN	6.72	90.52	204.2	103.8	90.521
	1	UNKNOWN	9.59	9.48	15.4	10.9	9.479
▶							
	Total			100.00	219.6	114.7	100.000

NMR spectra of (*R*)-mandelic acid complexes used to determine enantiomeric excesses

(3,4-Dichlorophenyl)hydroxyacetonitrile

Racemate

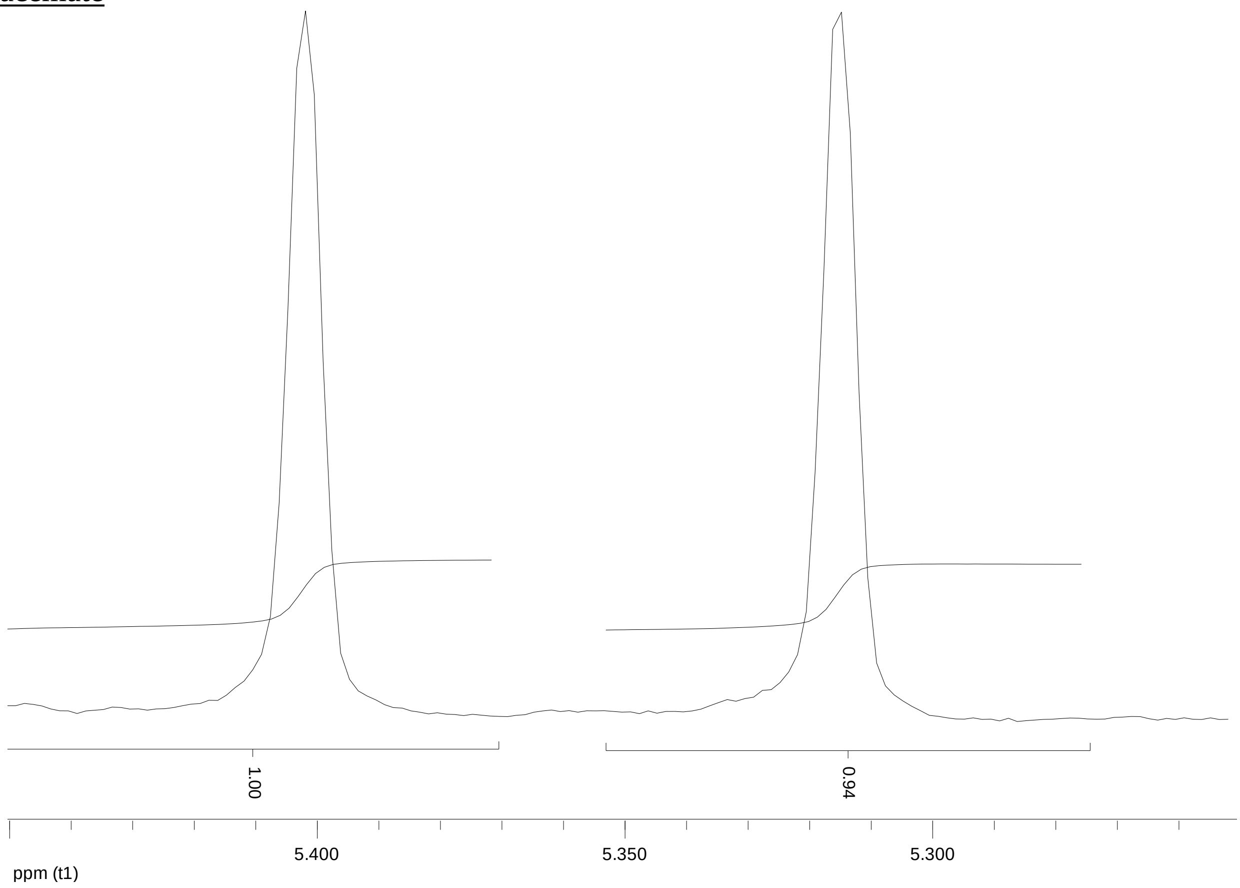

Sample prepared using catalyst **2** in propylene carbonate (Table 5, entry 2)

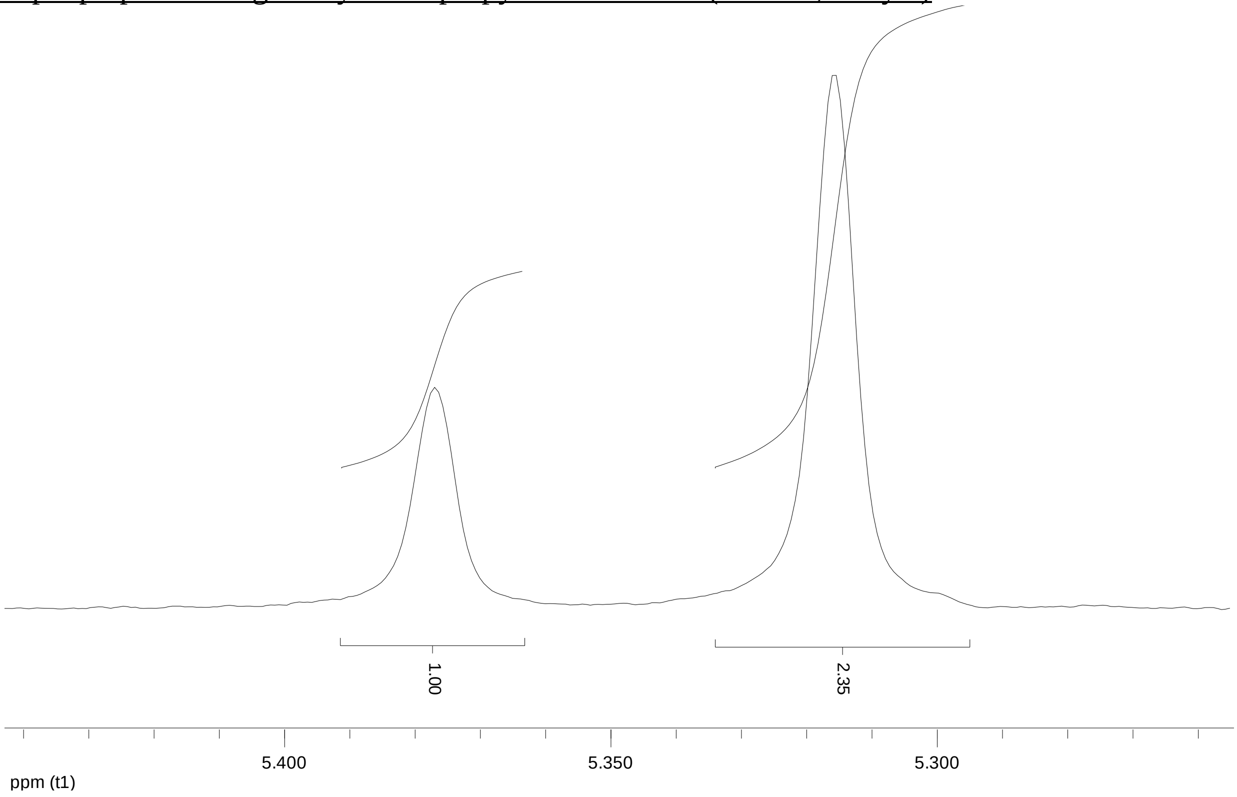

(3,4-Dimethylphenyl)hydroxyacetonitrile

Racemate

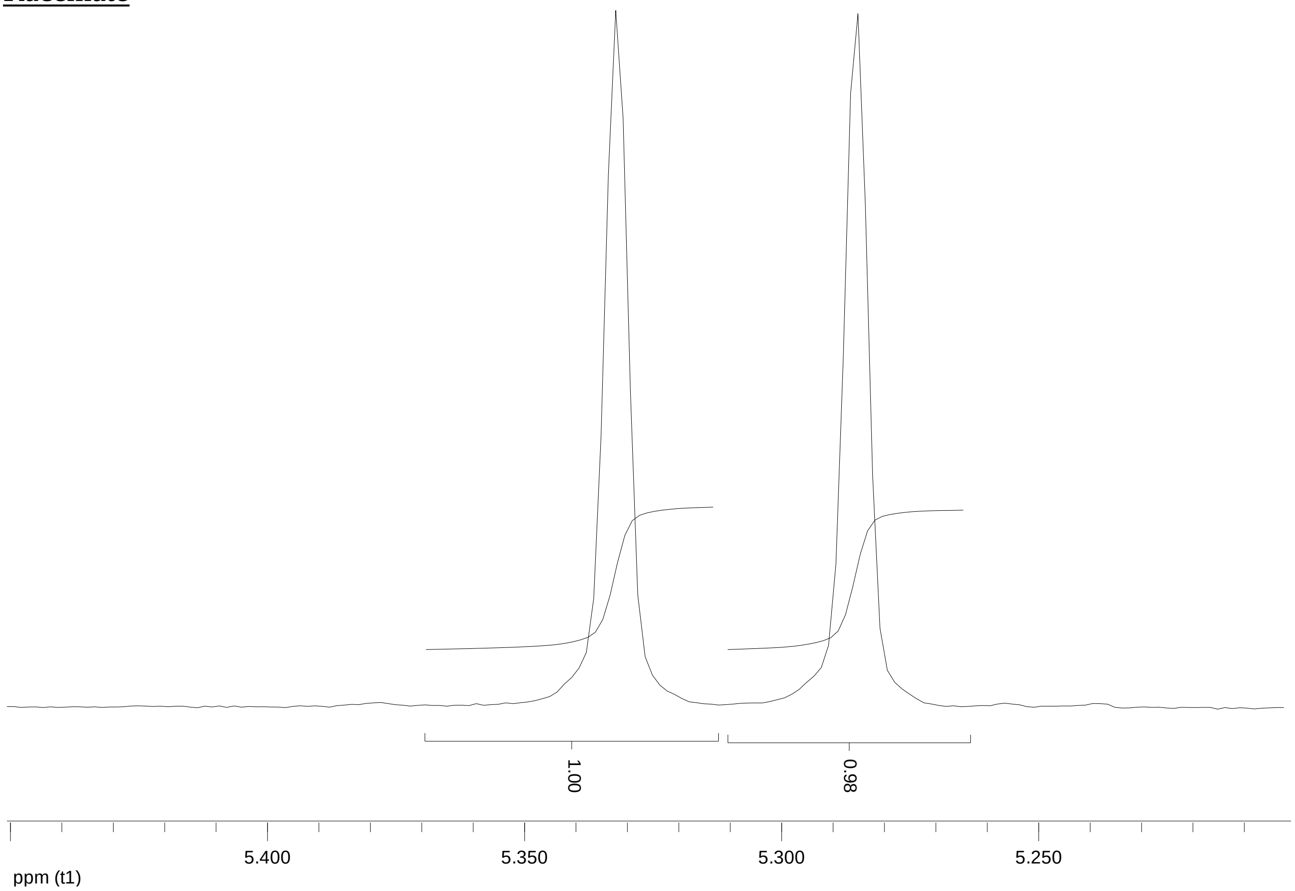

Sample prepared using catalyst **2** in propylene carbonate (Table 5, entry 11)

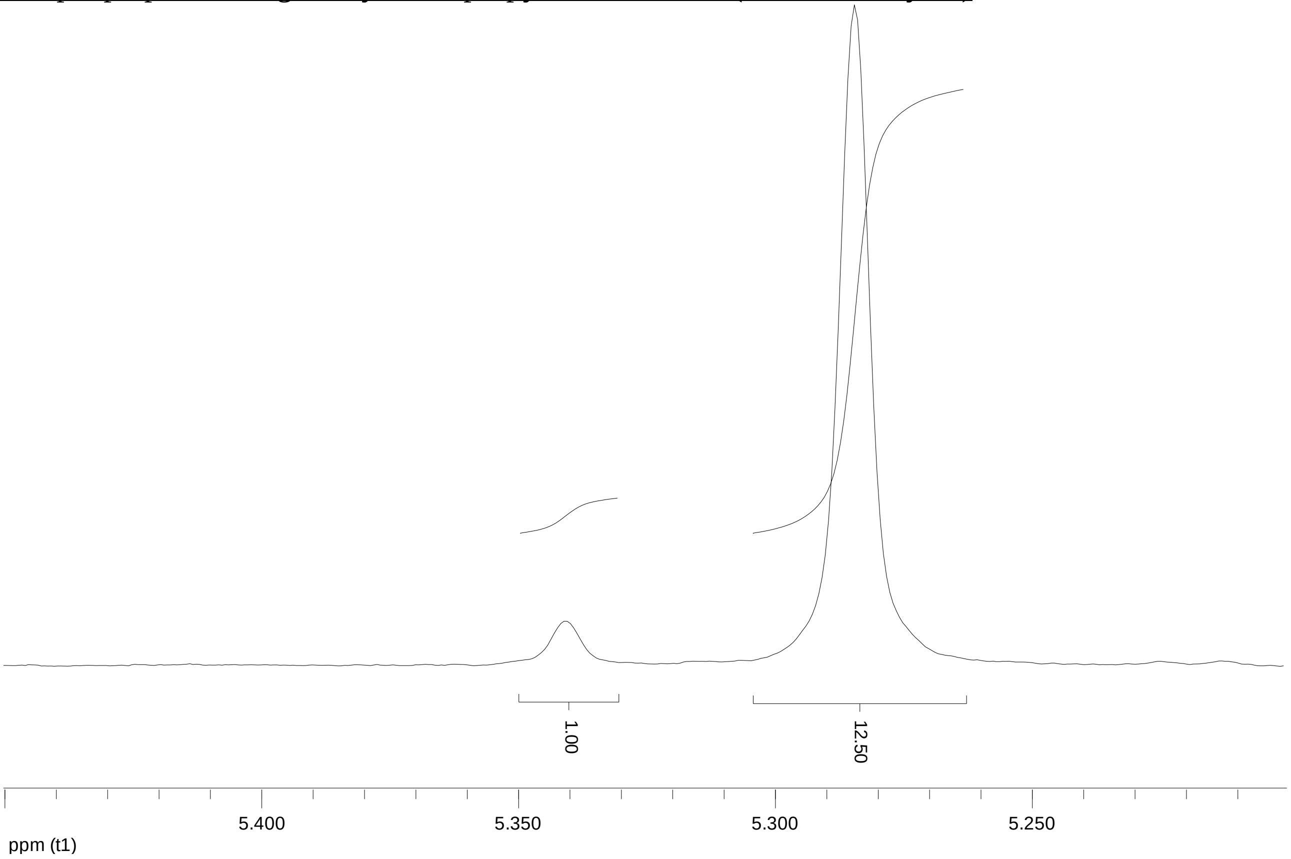

Kinetic plots used to determine the order with respect to catalyst concentration

The units for the vertical scales are $\ln[(B_0A_t)/(B_tA_0)]/(A_0–B_0)$, where A = [PhCHO], B = [Me_3SiCN], and the subscripts 0 and t refer to initial concentrations and concentrations at time t, respectively.

0.2 mol % catalyst run 1

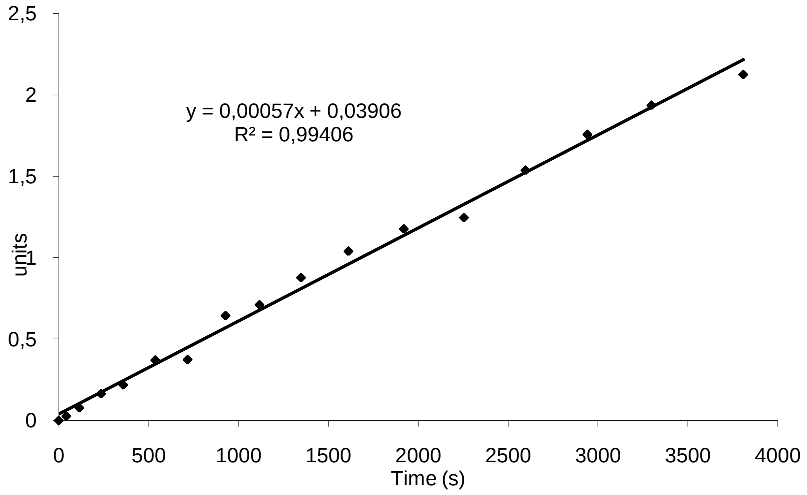

0.2 mol % catalyst run 2

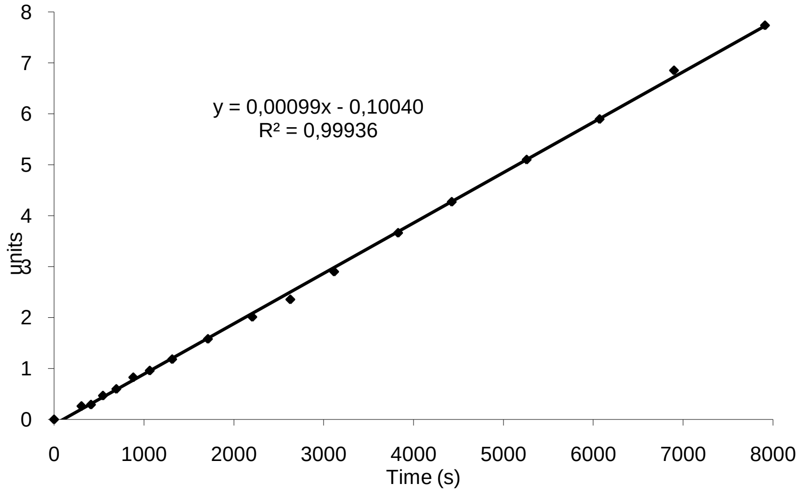

0.2 mol % catalyst run 3

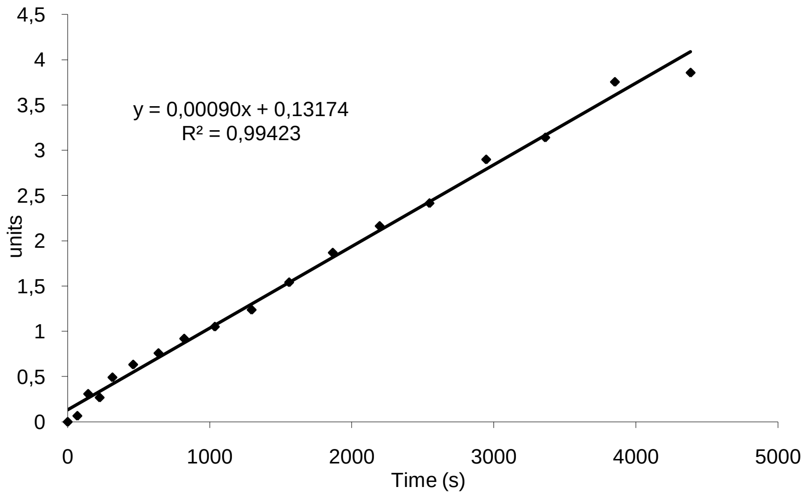

0.3 mol % catalyst run 1

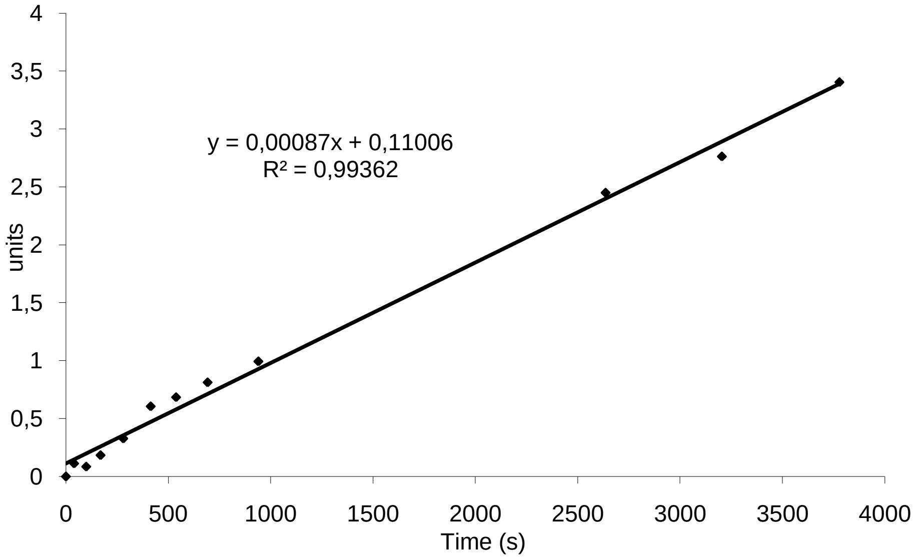

0.3 mol % catalyst run 2

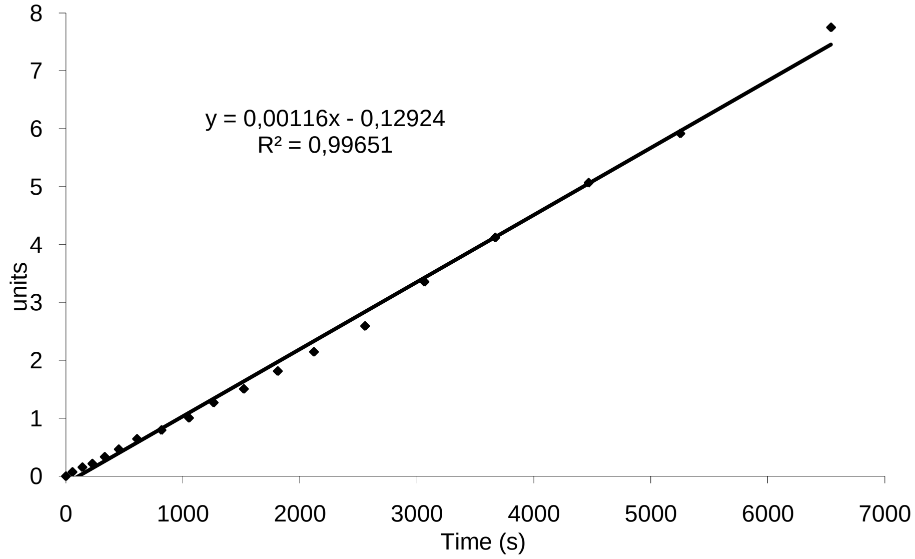

0.3 mol % catalyst run 3

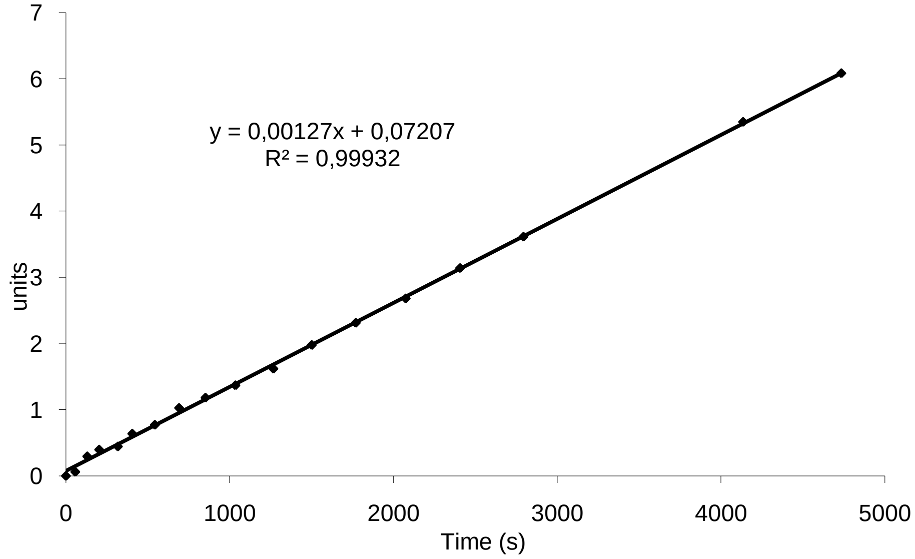

0.4 mol % catalyst run 1

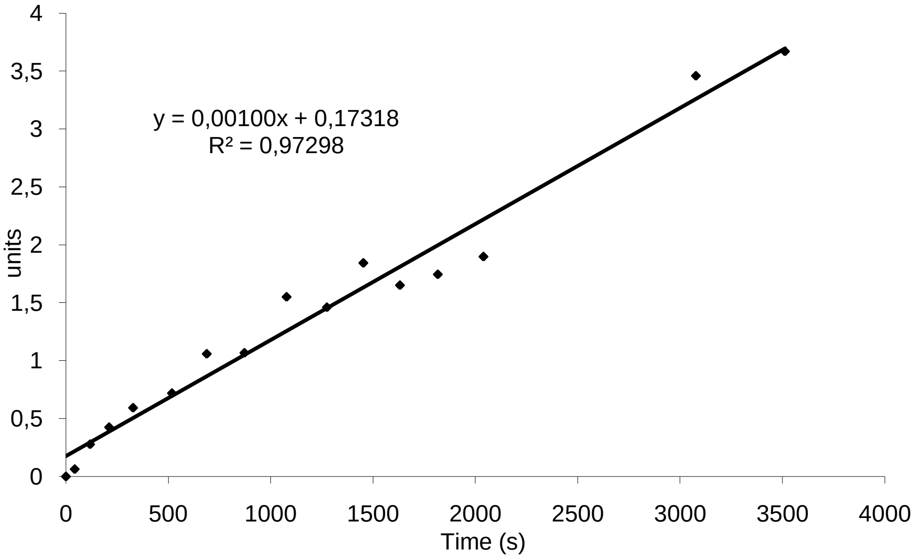

0.4 mol % catalyst run 2

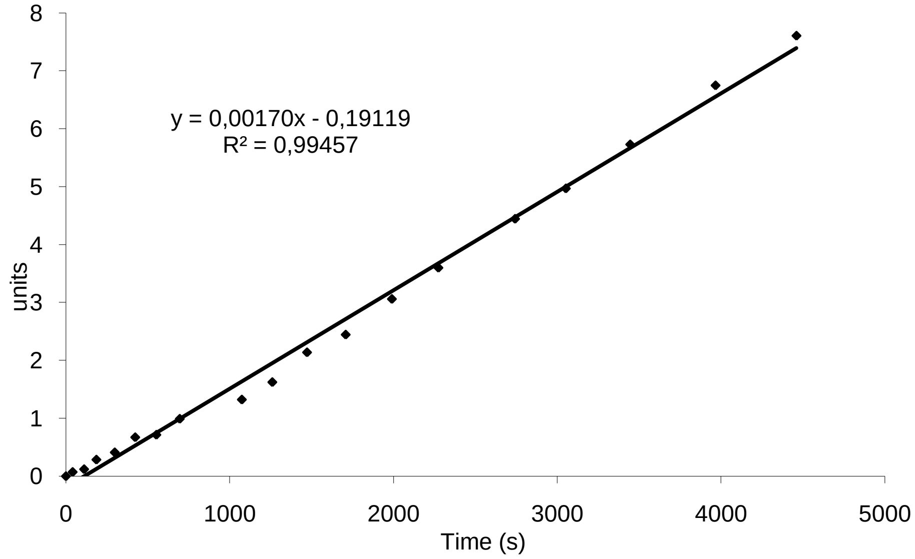

0.4 mol % catalyst run 3

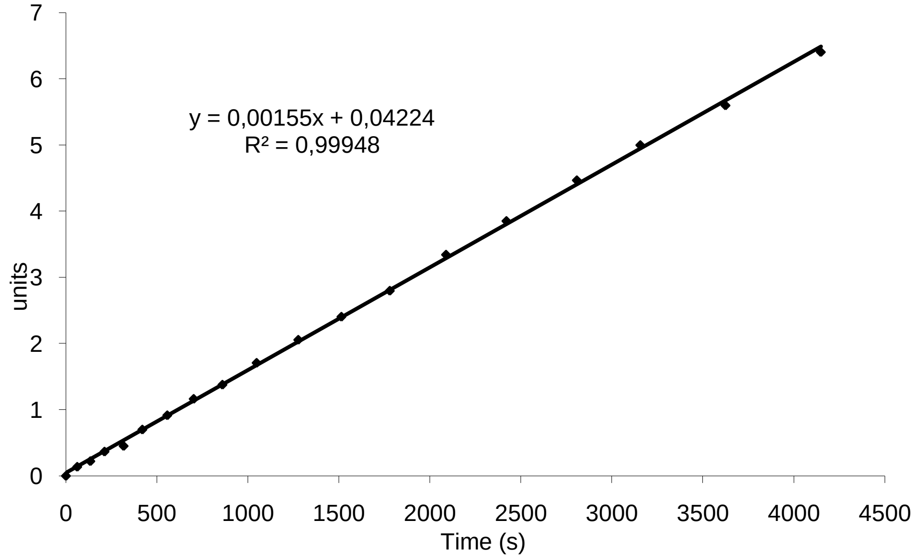

0.6 mol % catalyst run 1

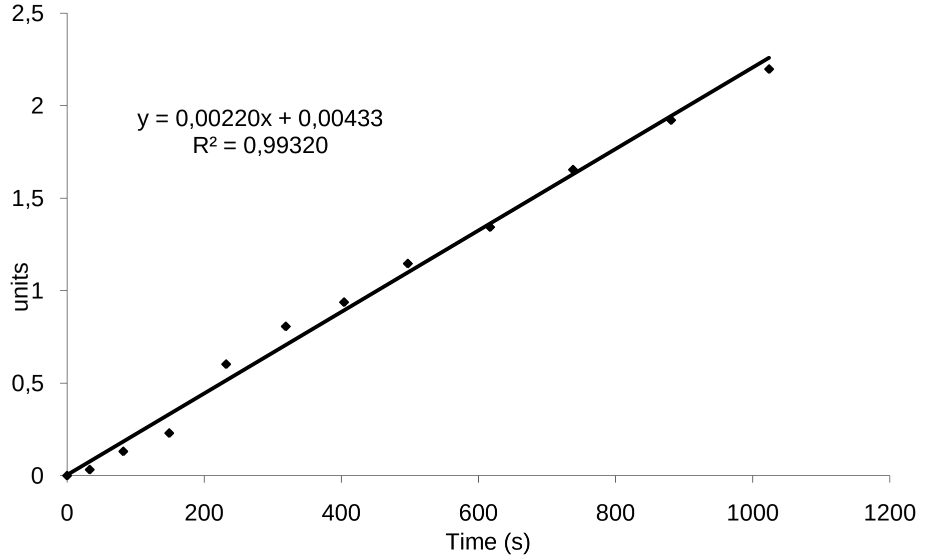

0.6 mol % catalyst run 2

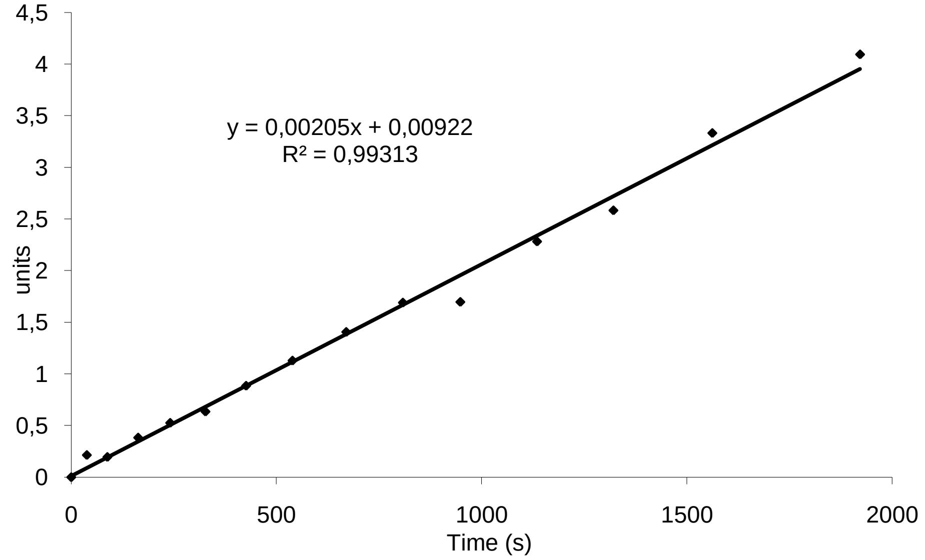

0.6 mol % catalyst run 3

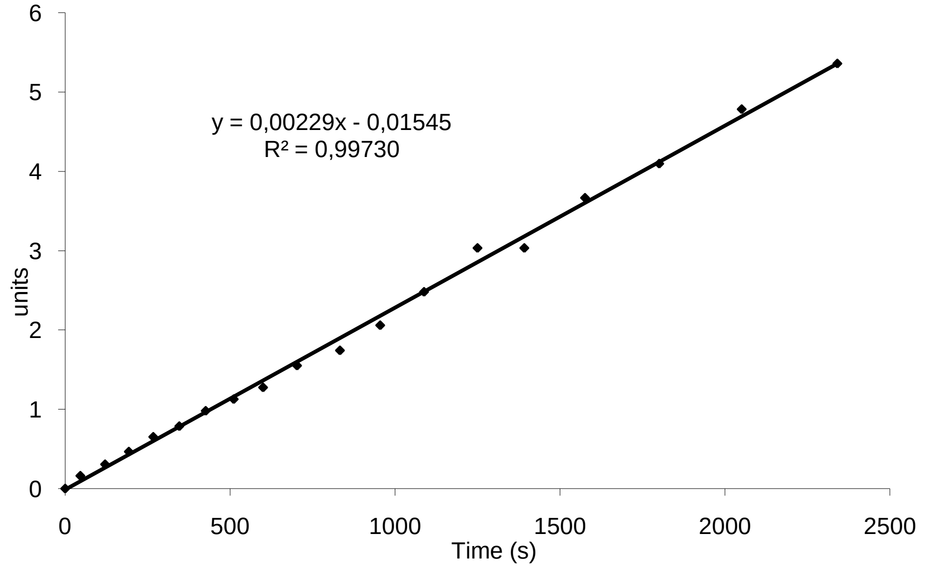

0.8 mol % catalyst run 1

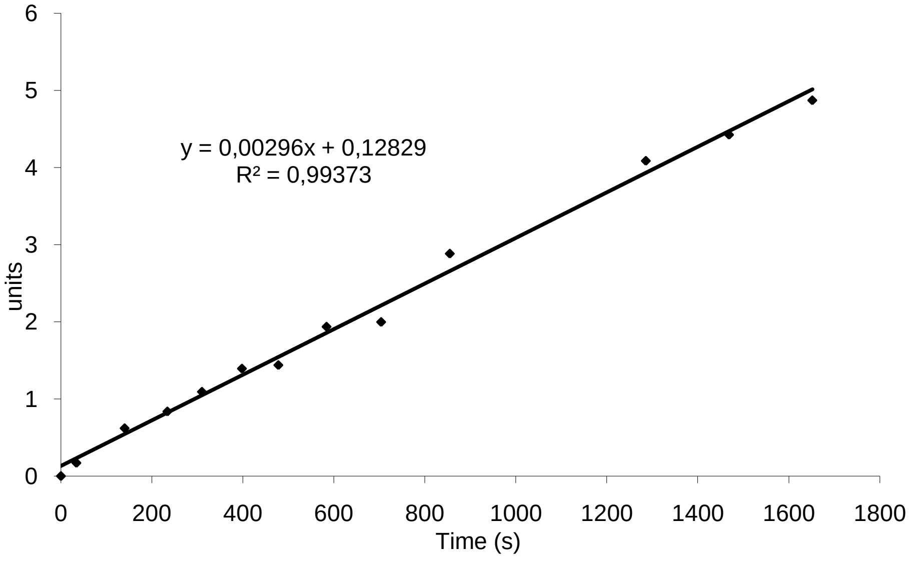

0.8 mol % catalyst run 2

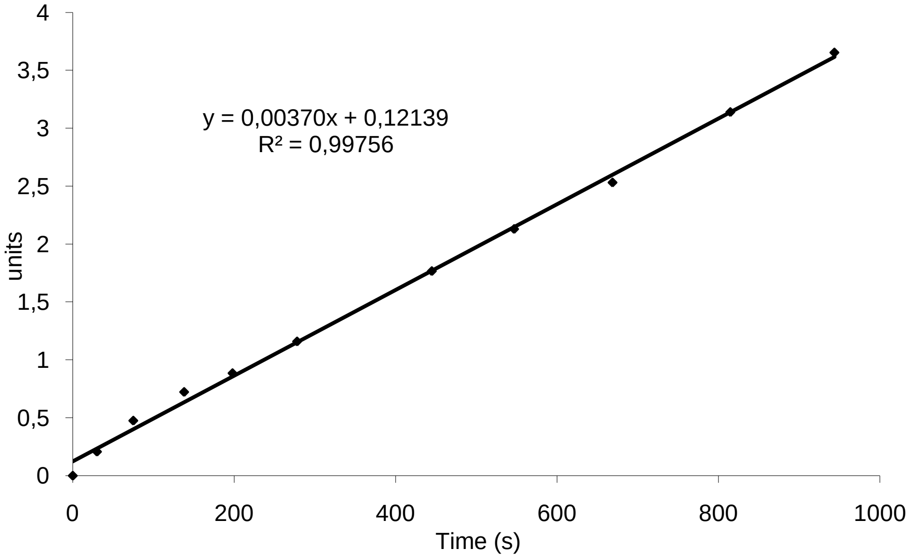

0.8 mol % catalyst run 3

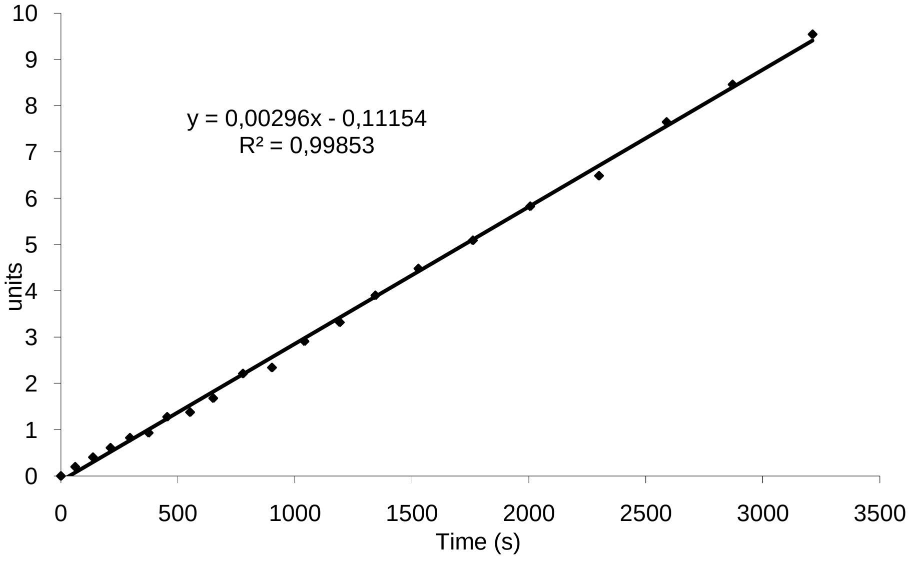

Plot of log([2]) against log($k_{2obs\ avg}$)

Since $k_{2obs} = k_2[\mathbf{2}]^x$, $\log(k_{2obs}) = \log(k_2) + x\log([\mathbf{2}])$, therefore a plot of log([2]) against log(k_{2obs}) provides an alternative way of determining the order with respect to catalyst **2**. The plot is shown below and its slope is consistent with an order of 1.0 with respect to catalyst.

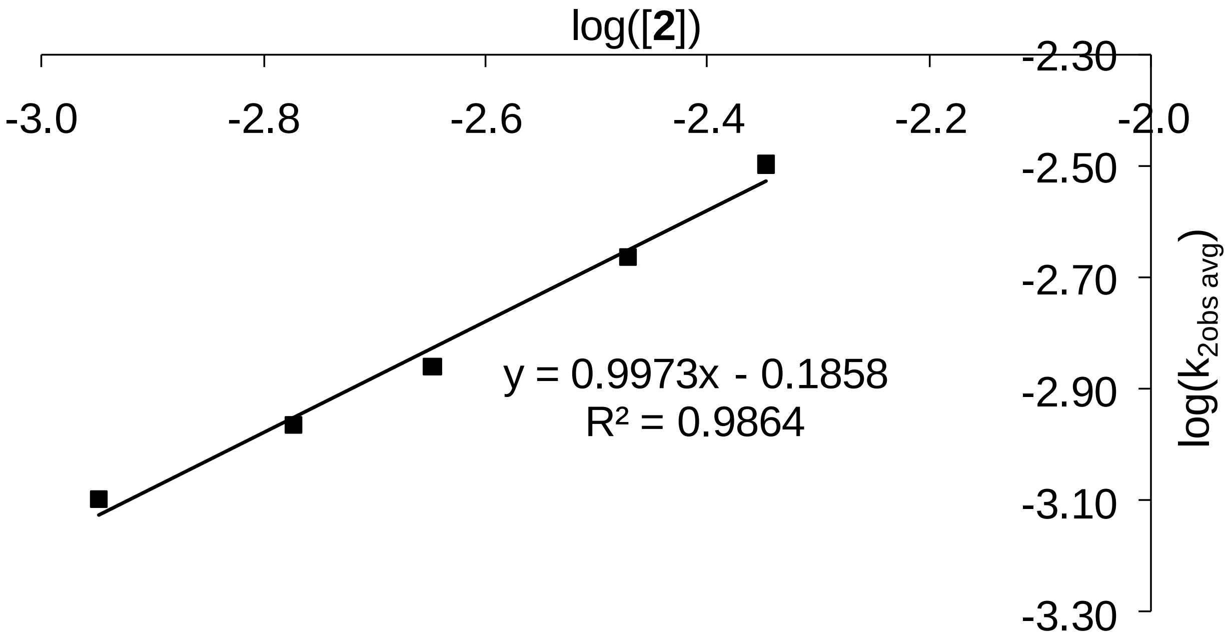

Kinetic plots used to determine the activation parameters in propylene carbonate

The units for the vertical scales are $\ln[(B_0A_t)/(B_tA_0)]/(A_0-B_0)$, where A = [PhCHO], B = [Me_3SiCN], and the subscripts 0 and t refer to initial concentrations and concentrations at time t, respectively.

Kinetics at 253 K (run 1)

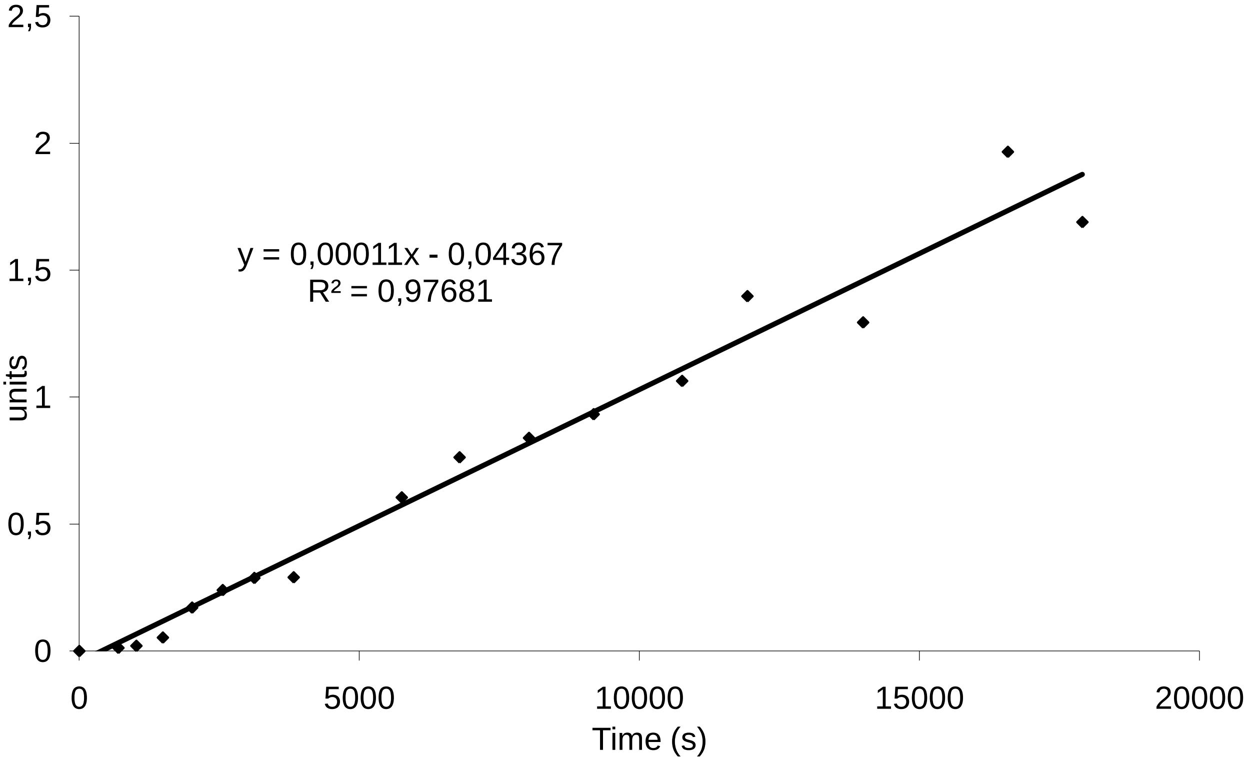

Kinetics at 253 K (run 2)

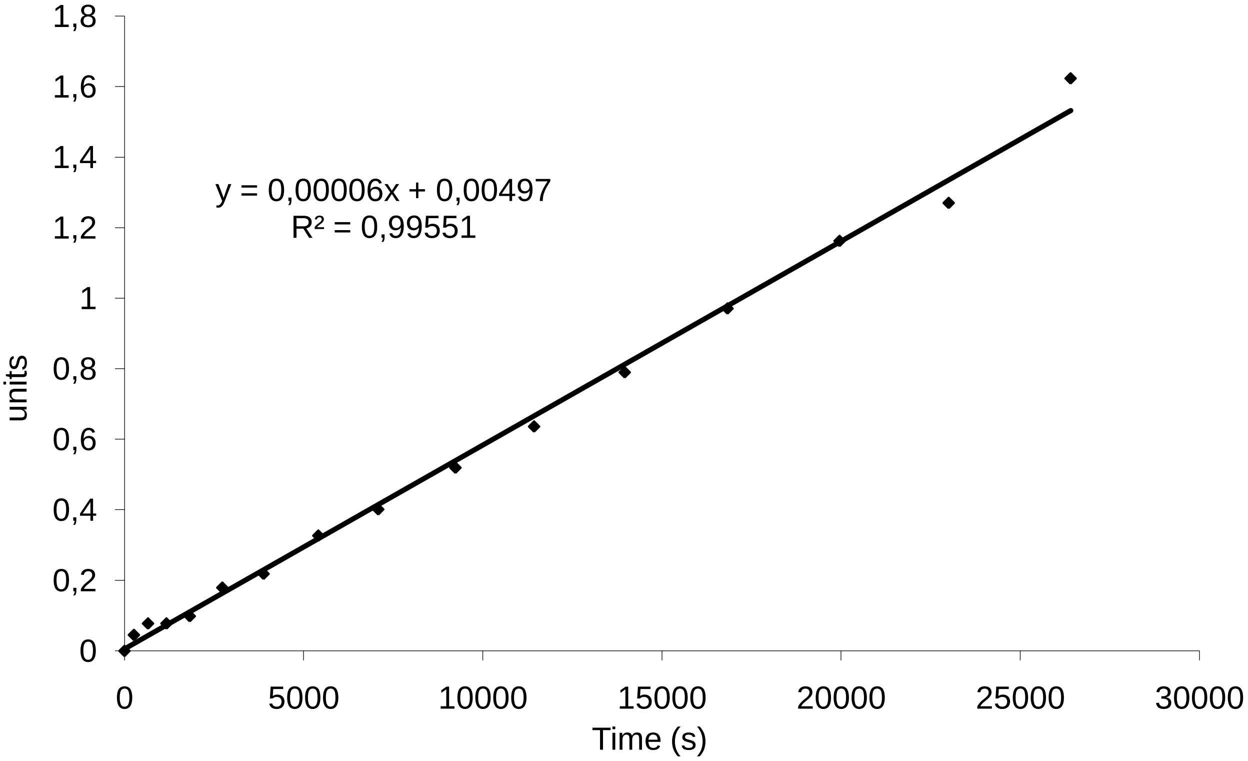

Kinetics at 263 K (run 1)

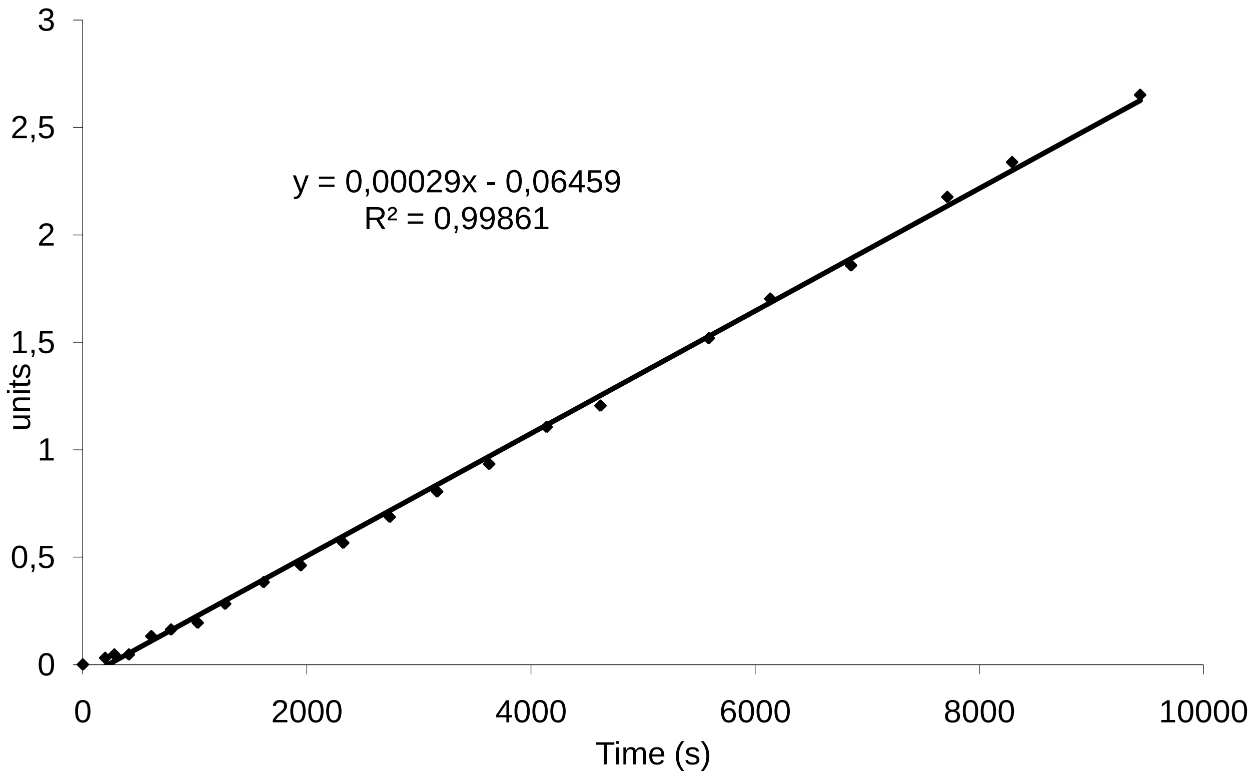

Kinetics at 263 K (run 2)

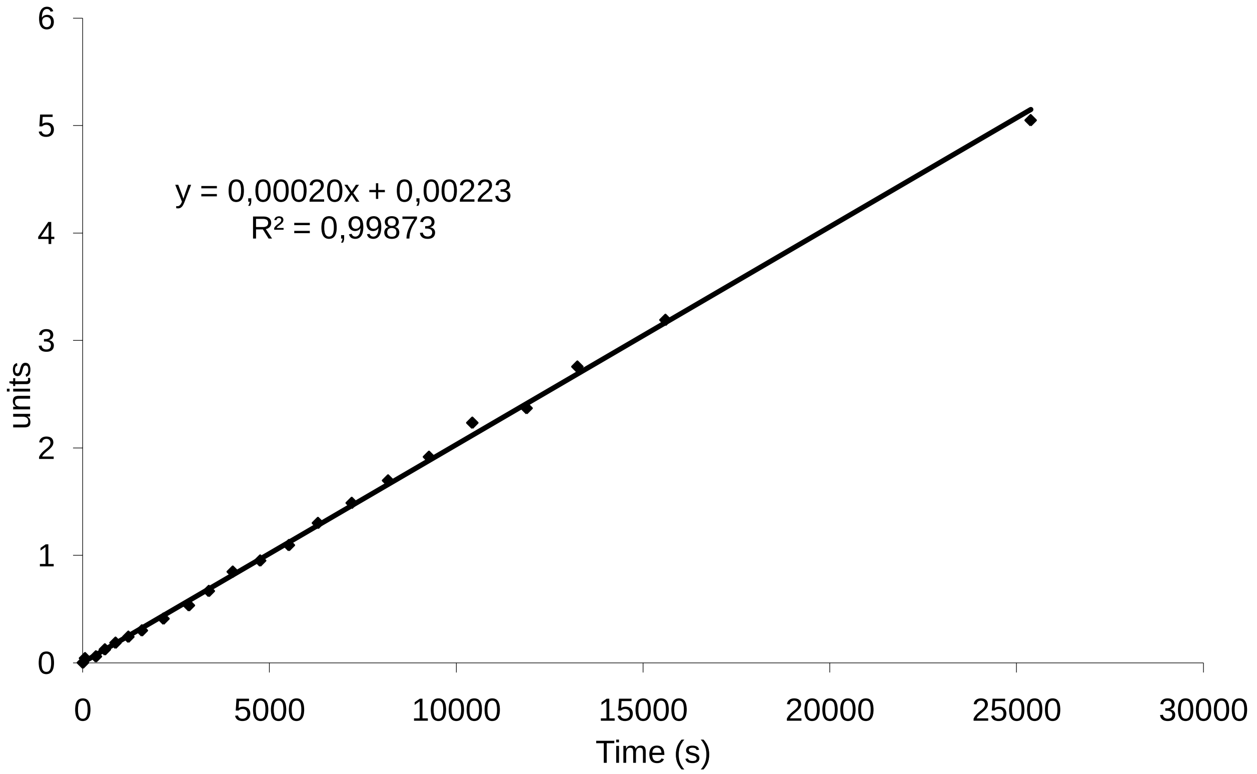

Kinetics at 273 K (run 1)

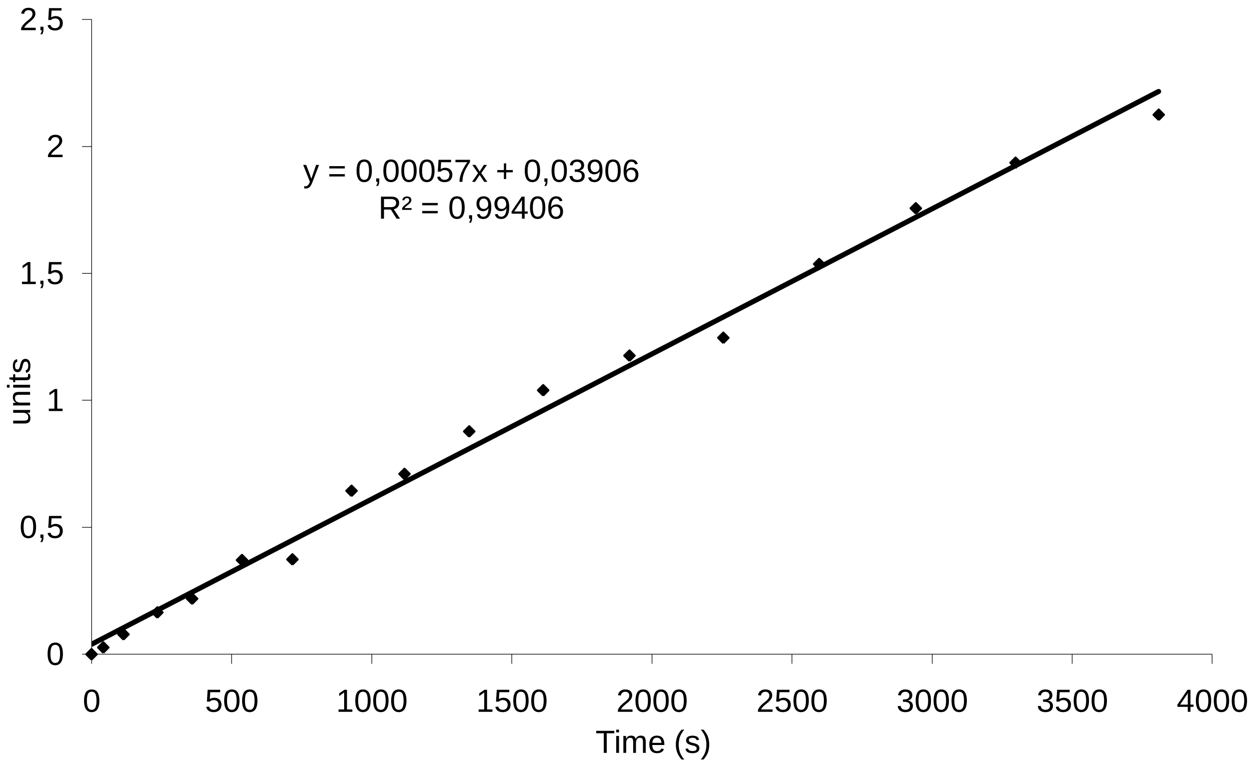

Kinetics at 273 K (run 2)

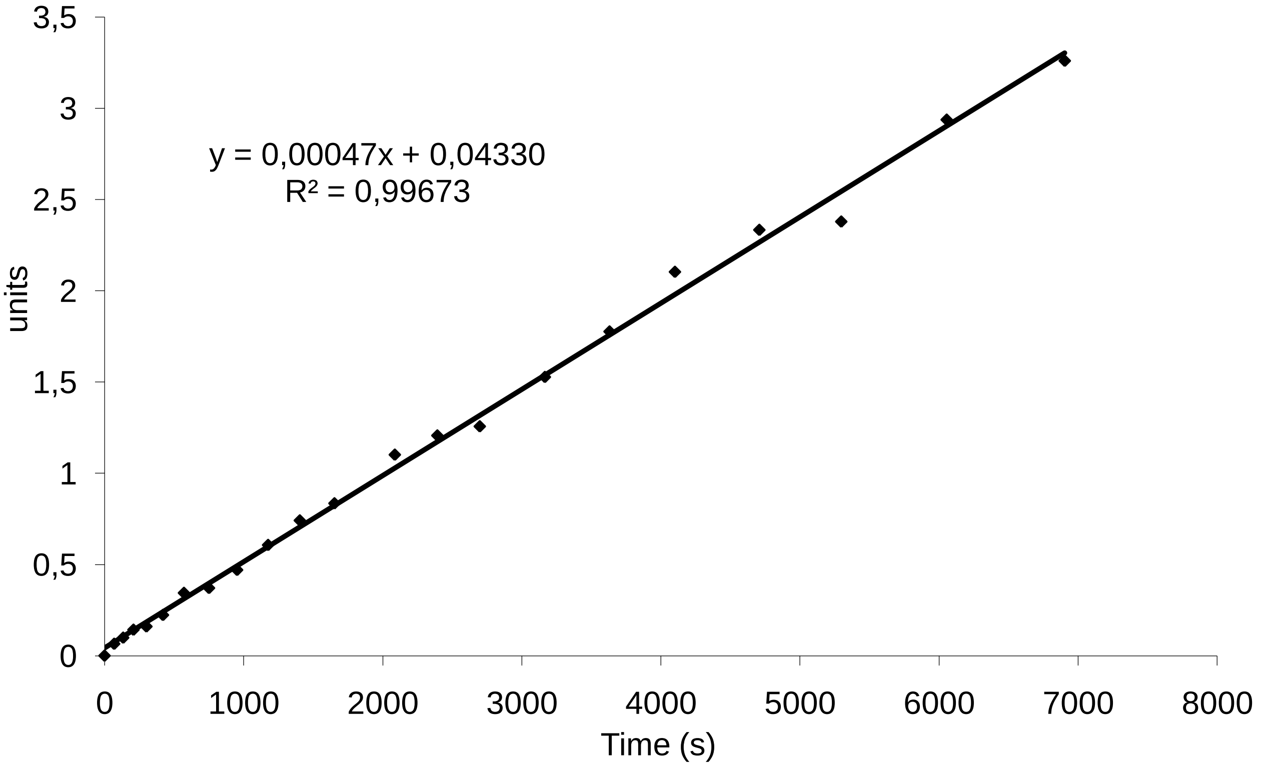

Kinetics at 283 K (run 1)

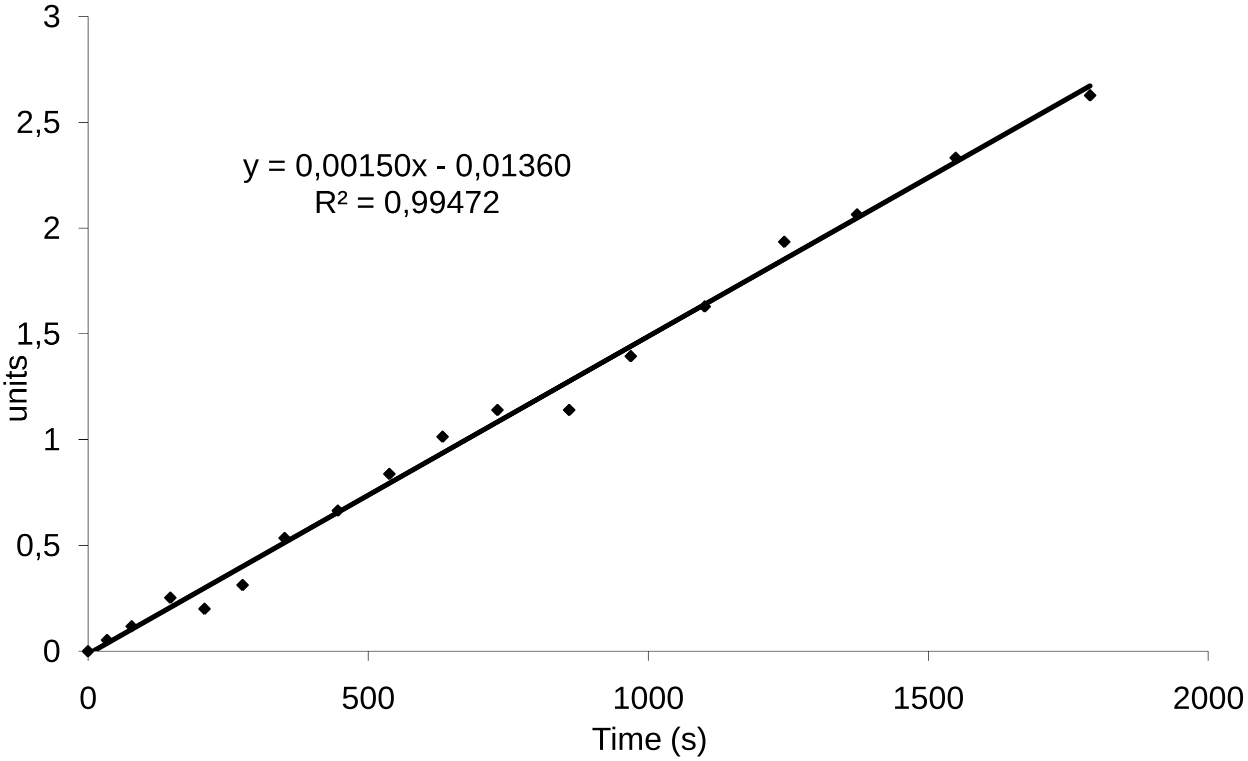

Kinetics at 283 K (run 2)

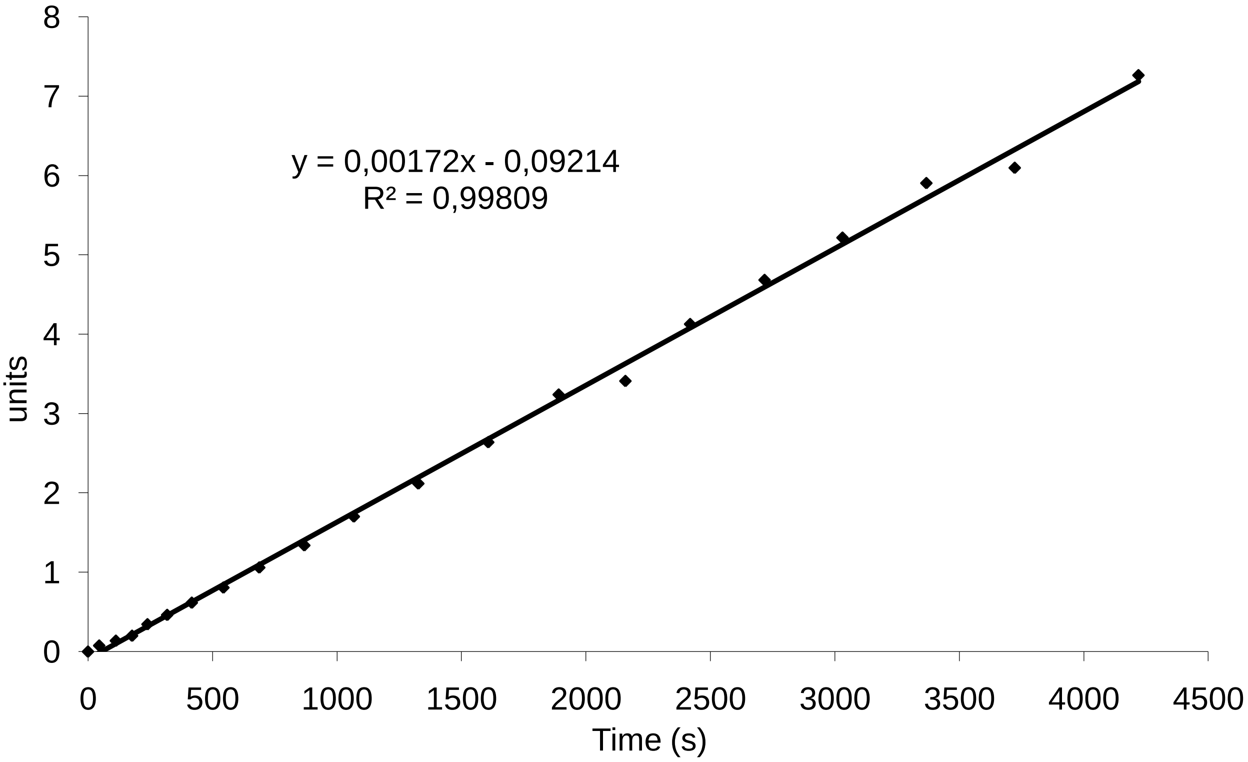

Kinetics at 293 K (run 1)

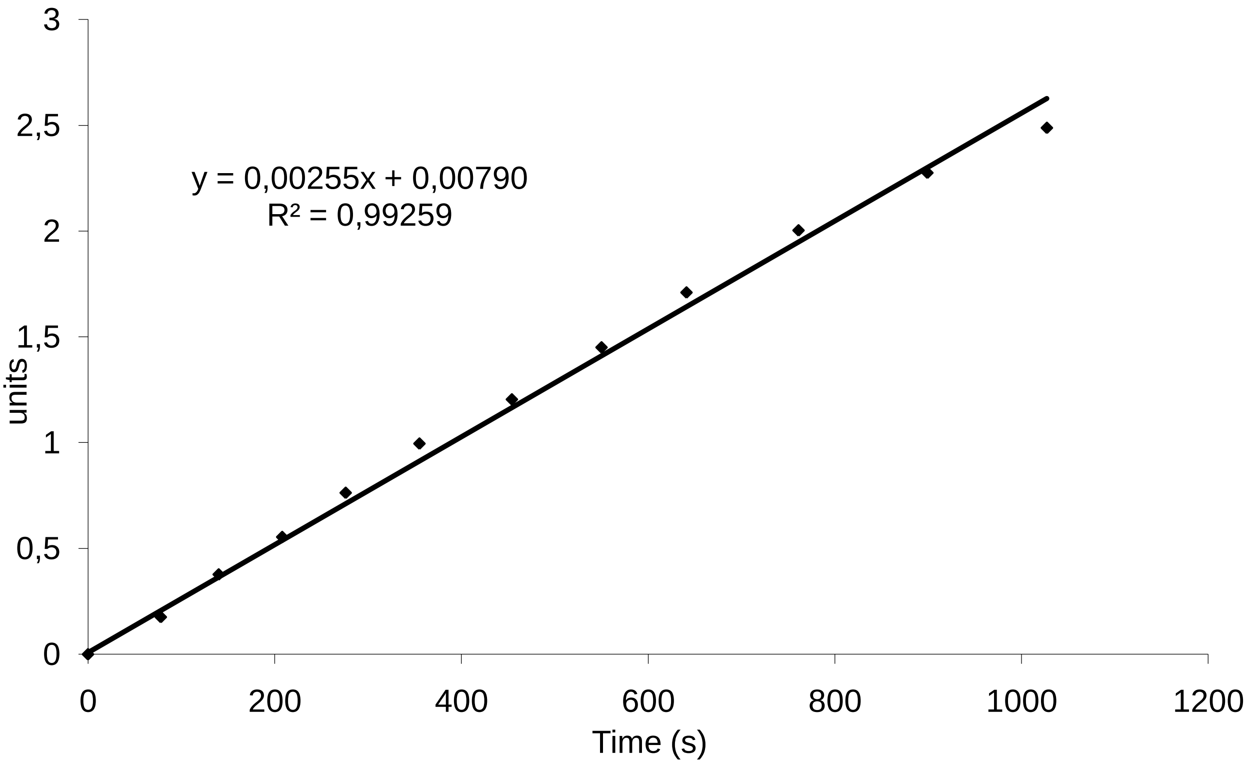

Kinetics at 293 K (run 1)

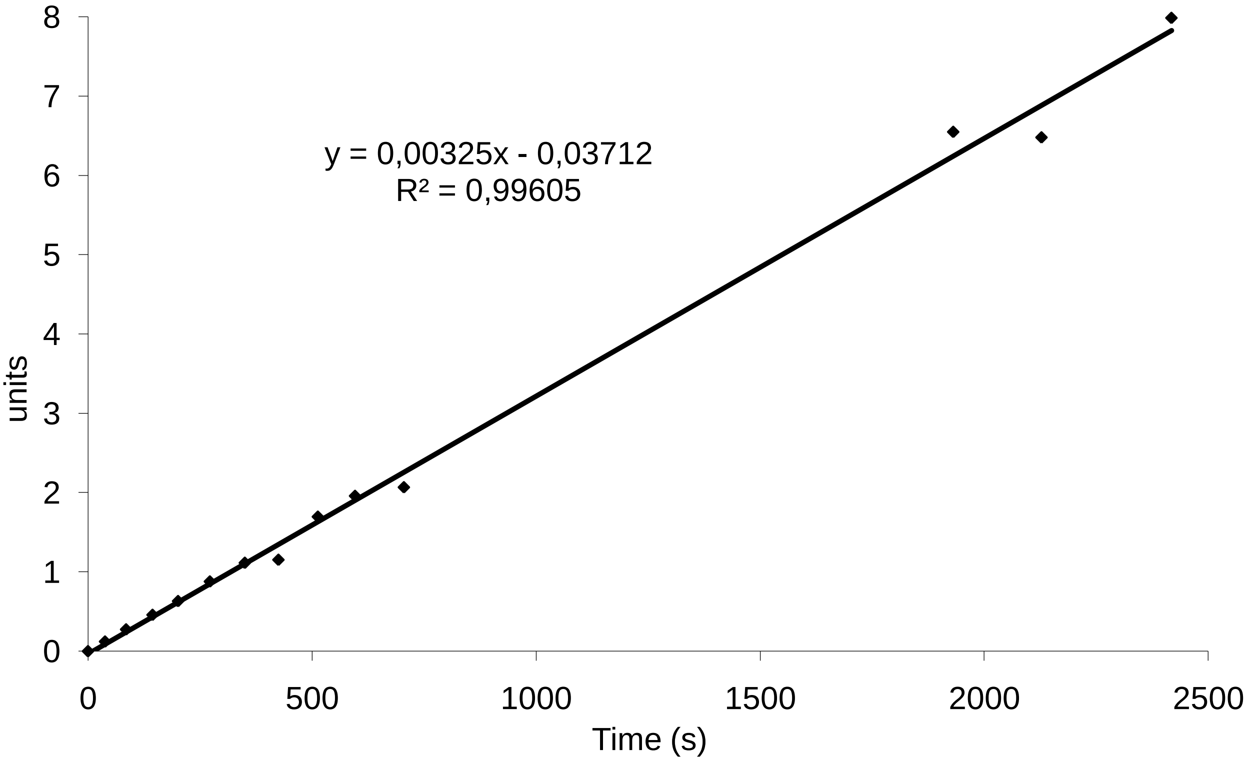

Kinetic plots used to construct the Hammett plot

The units for the vertical scales are $\ln[(B_0A_t)/(B_tA_0)]/(A_0–B_0)$, where A = [PhCHO], B = $[Me_3SiCN]$, and the subscripts 0 and t refer to initial concentrations and concentrations at time t, respectively.

Benzaldehyde run 1

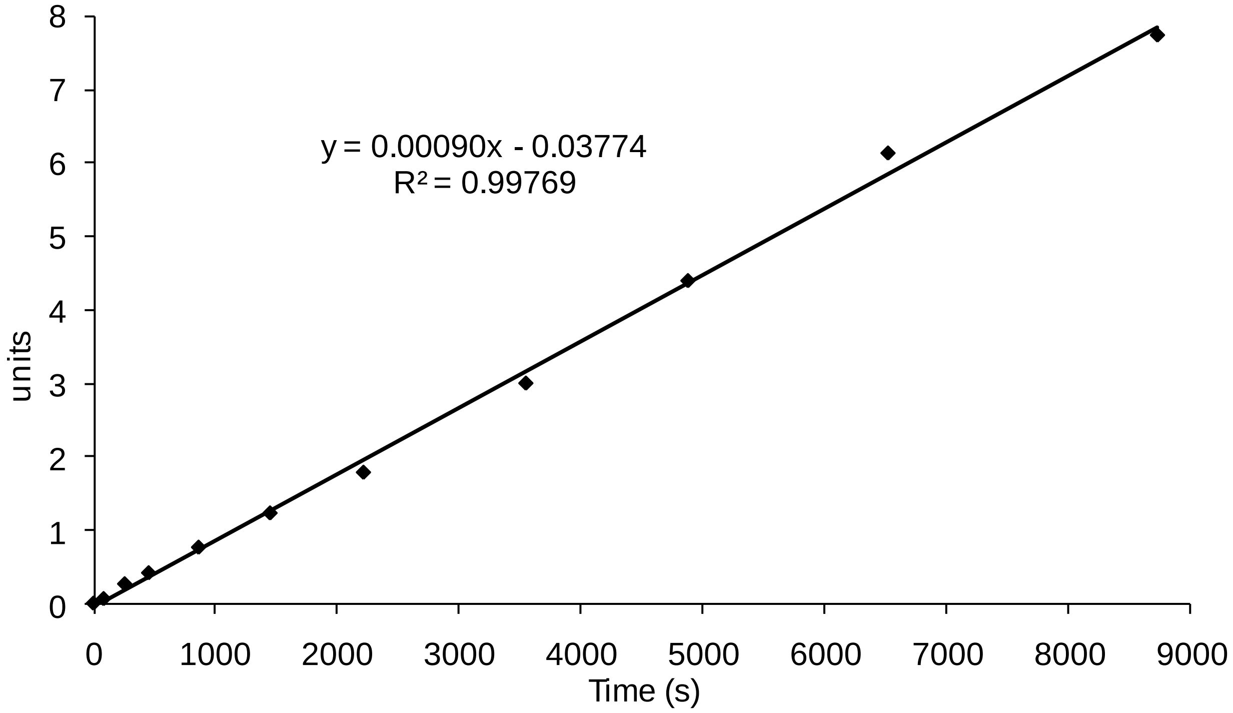

Benzaldehyde run 2

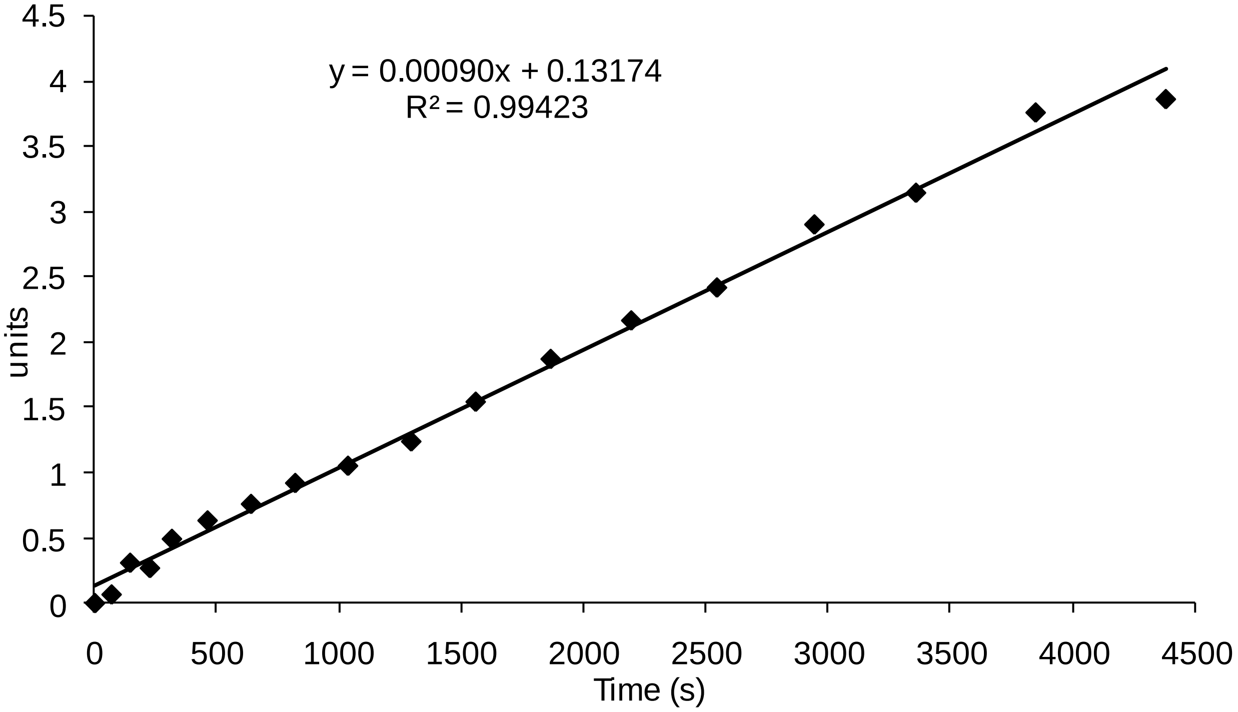

3,4-Dichlorobenzaldehyde run 1

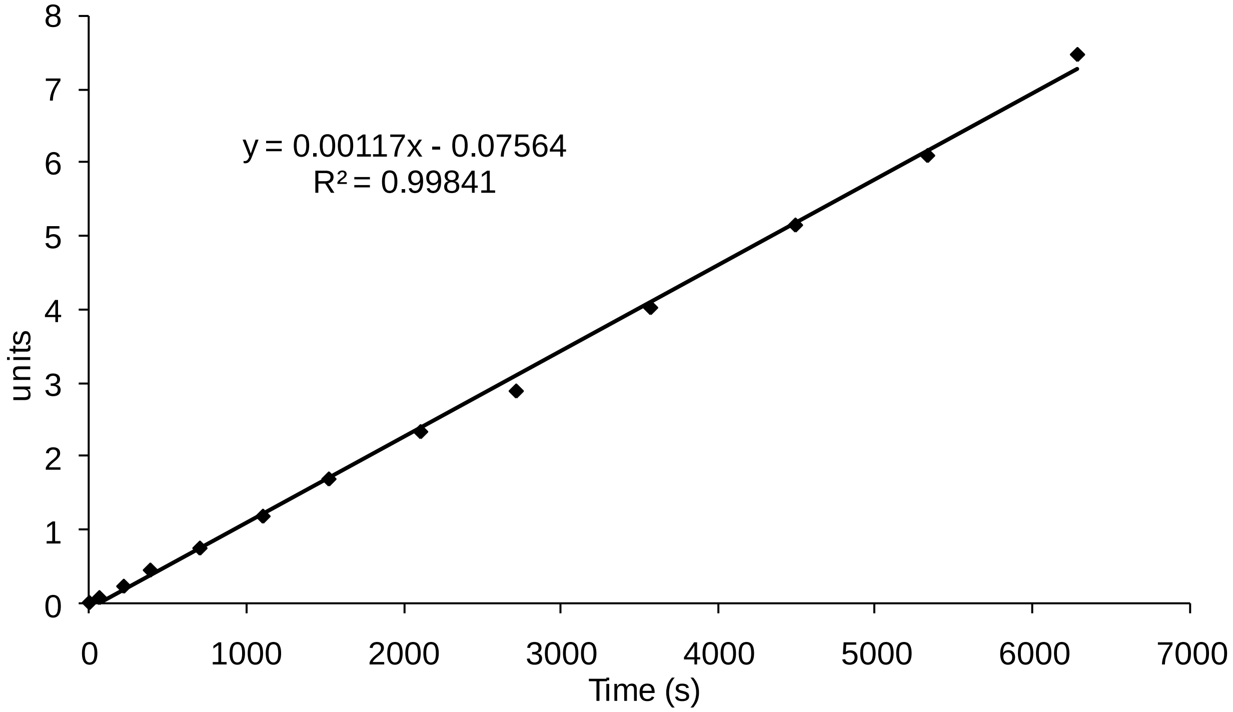

3,4-Dichlorobenzaldehyde run 2

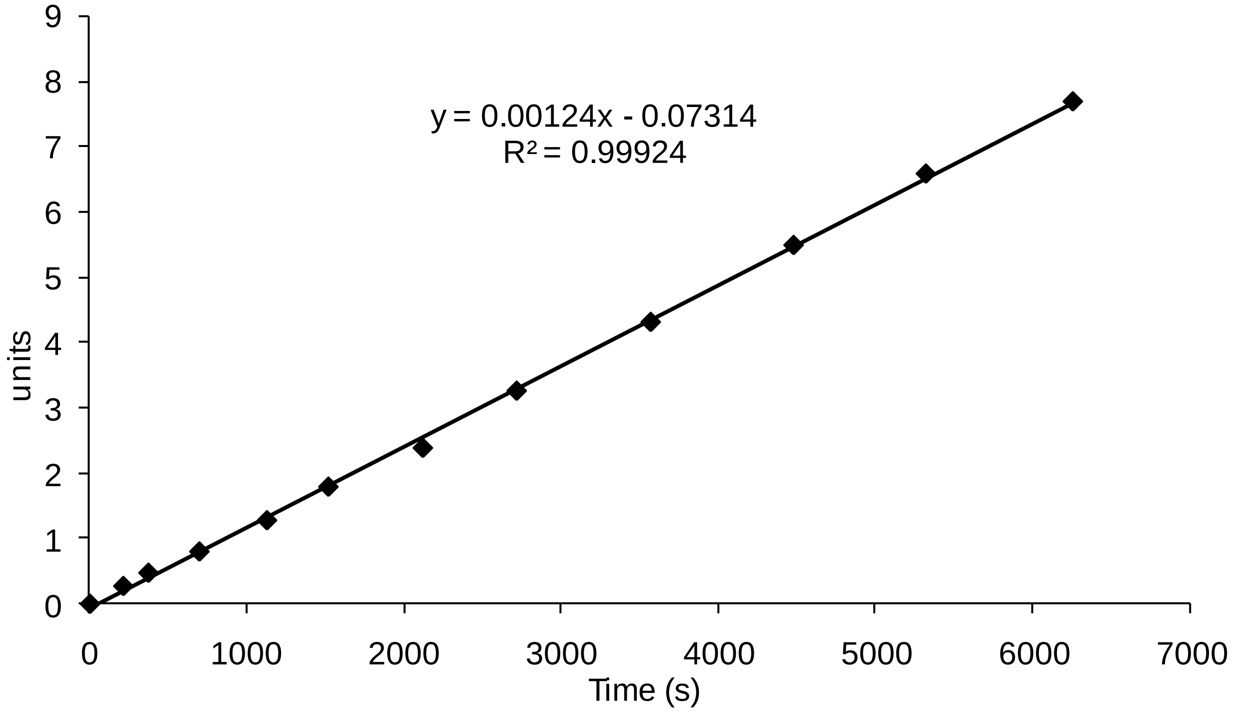

4-Chlorobenzaldehyde run 1

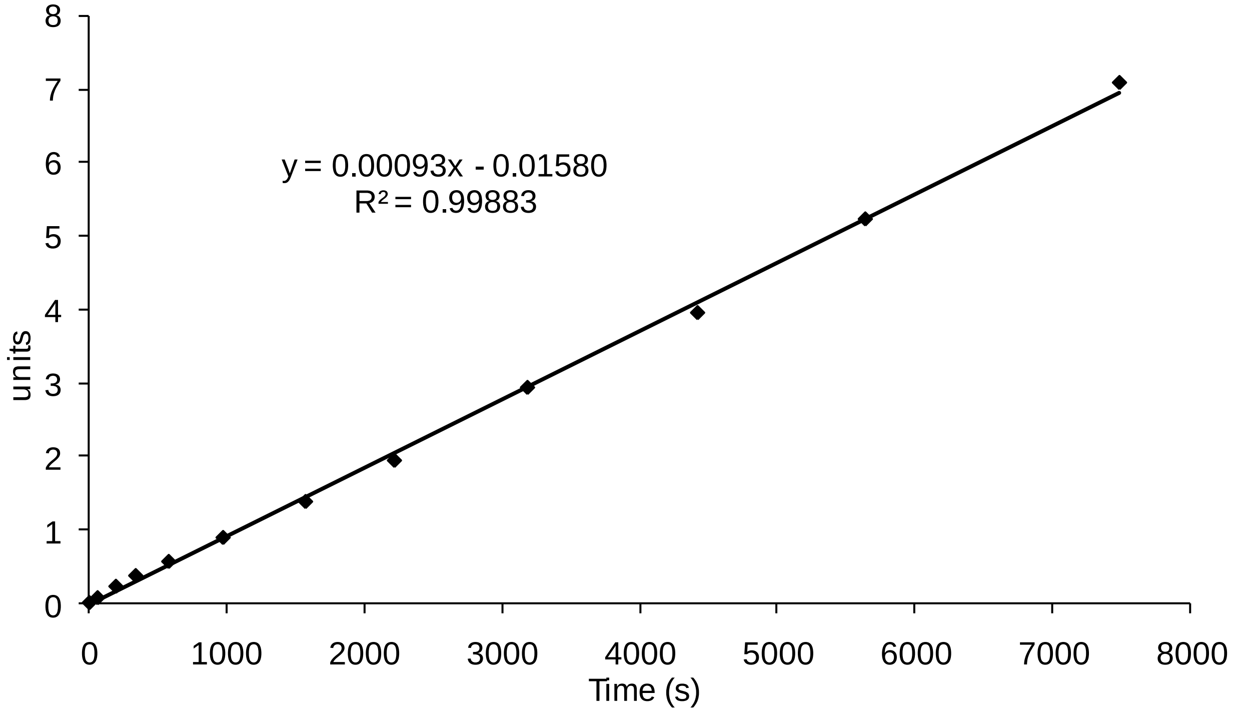

4-Chlorobenzaldehyde run 2

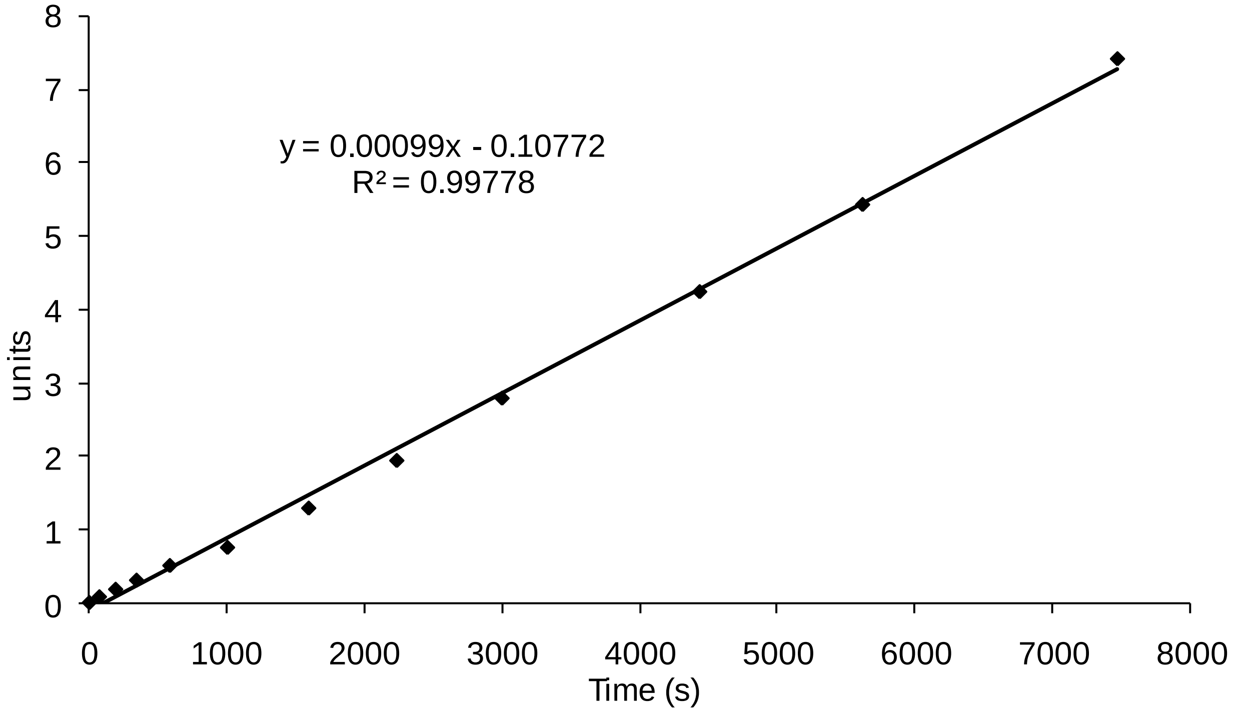

4-Methylbenzaldehyde run 1

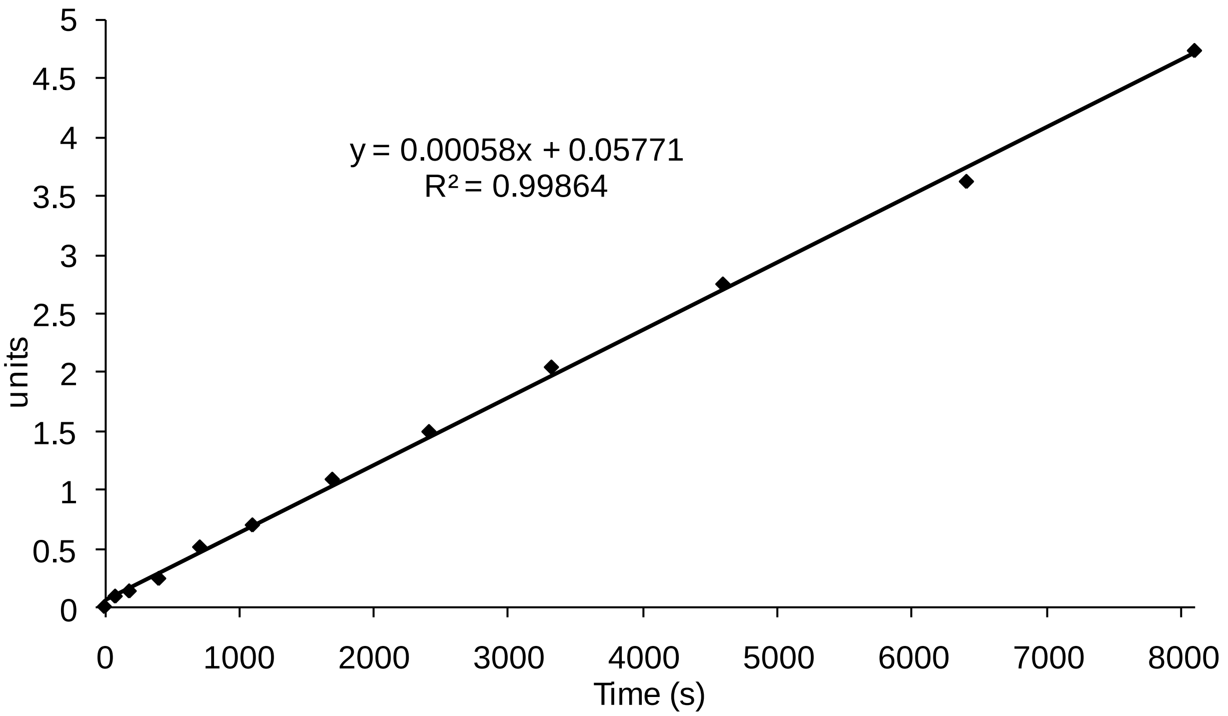

4-Methylbenzaldehyde run 2

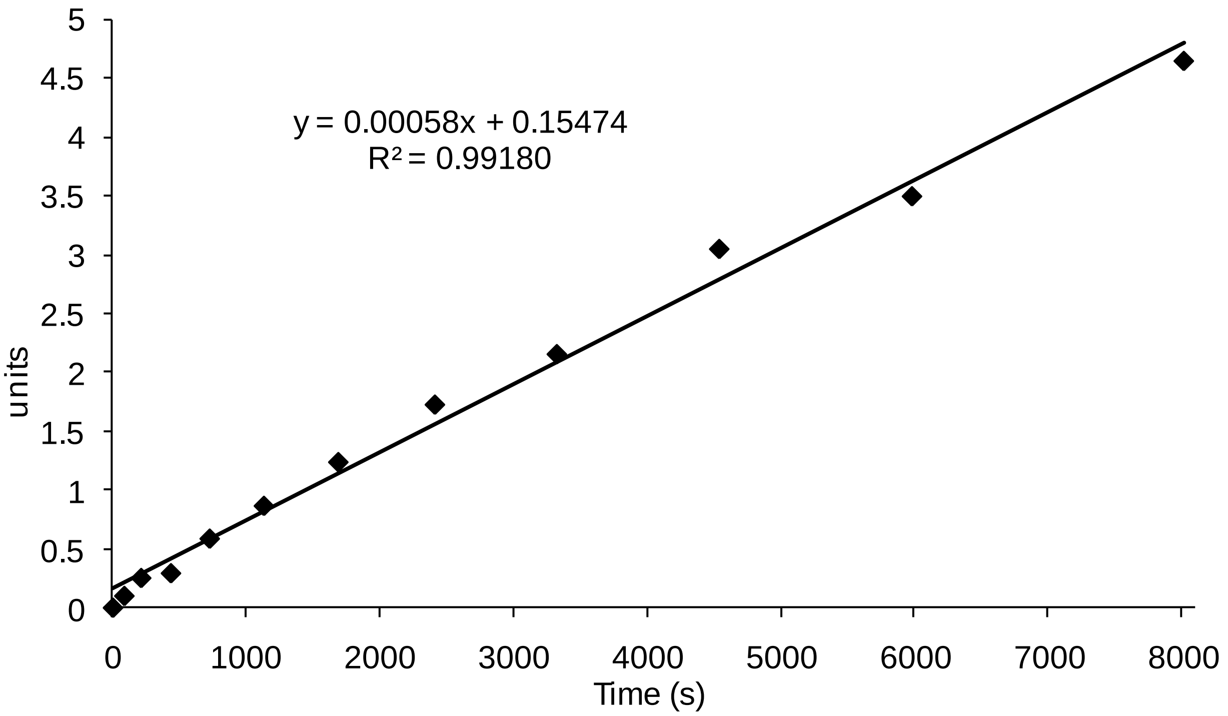

4-Fluorobenzaldehyde run 1

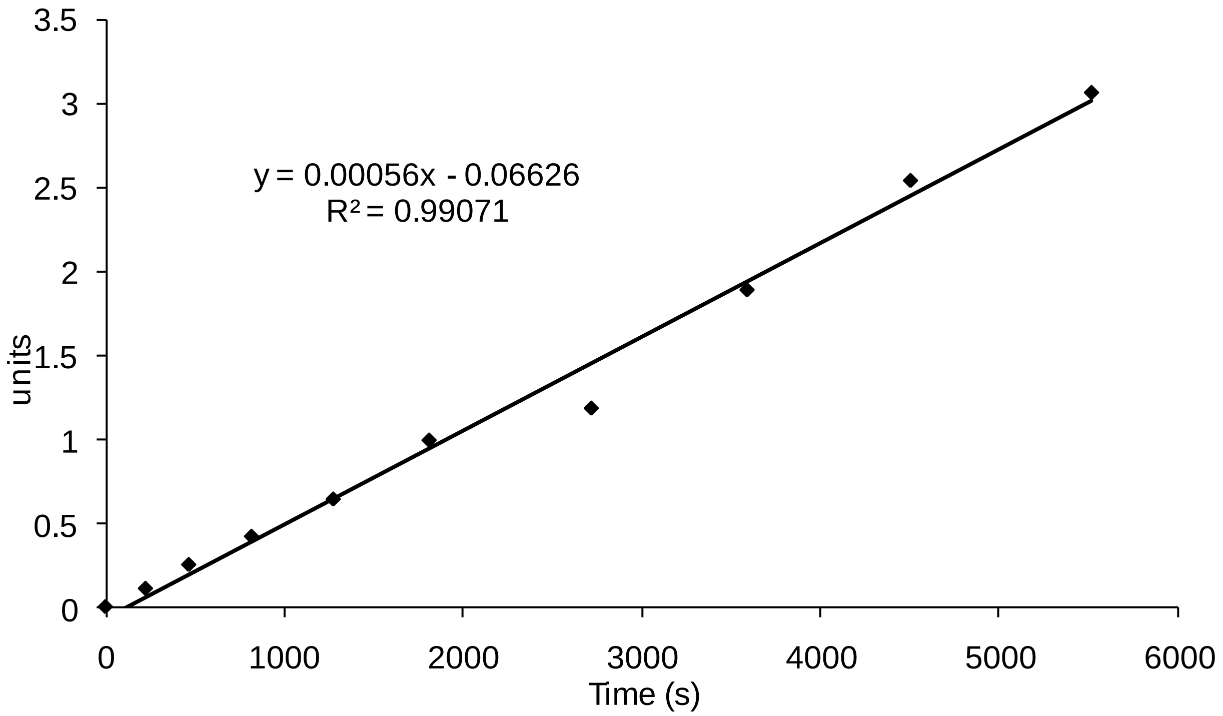

4-Fluorobenzaldehyde run 2

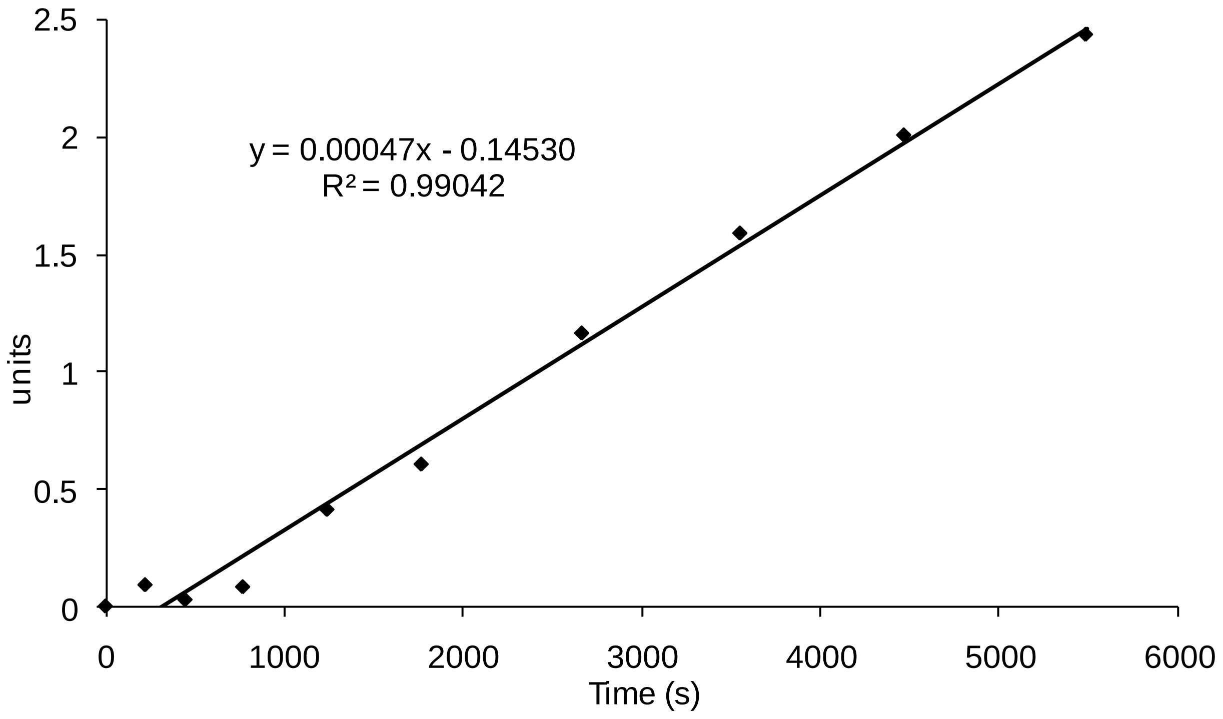

3-Fluorobenzaldehyde run 1

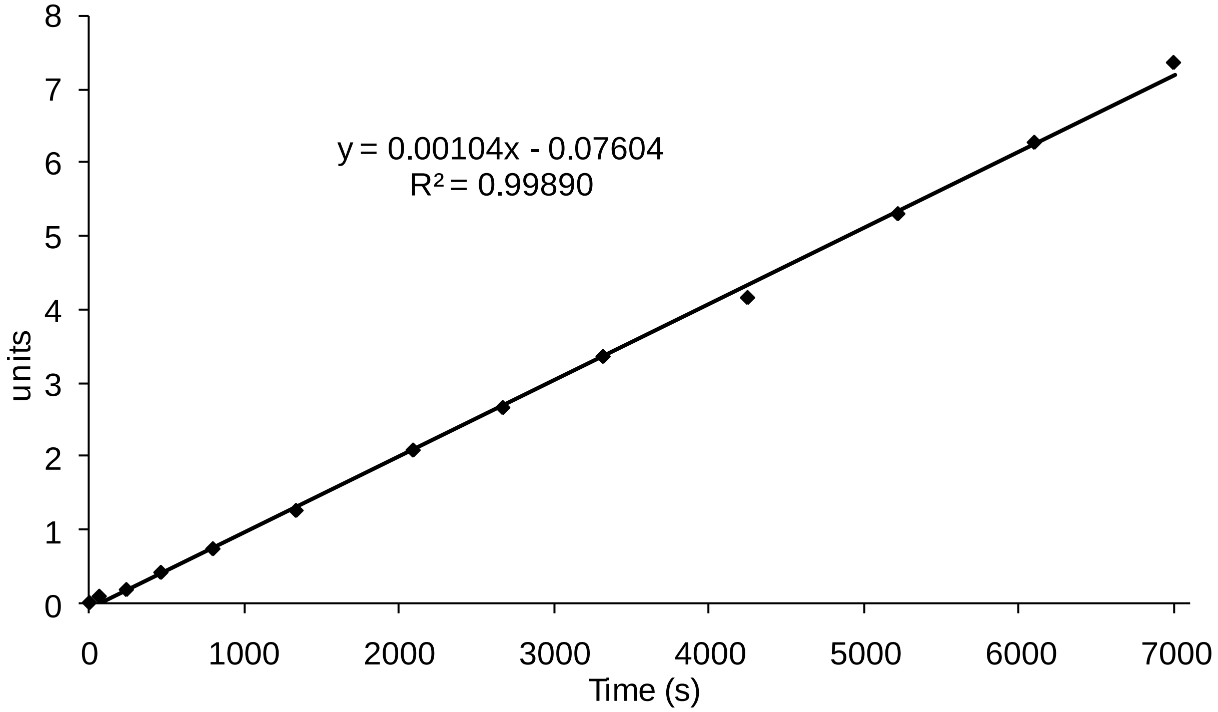

3-Fluorobenzaldehyde run 2

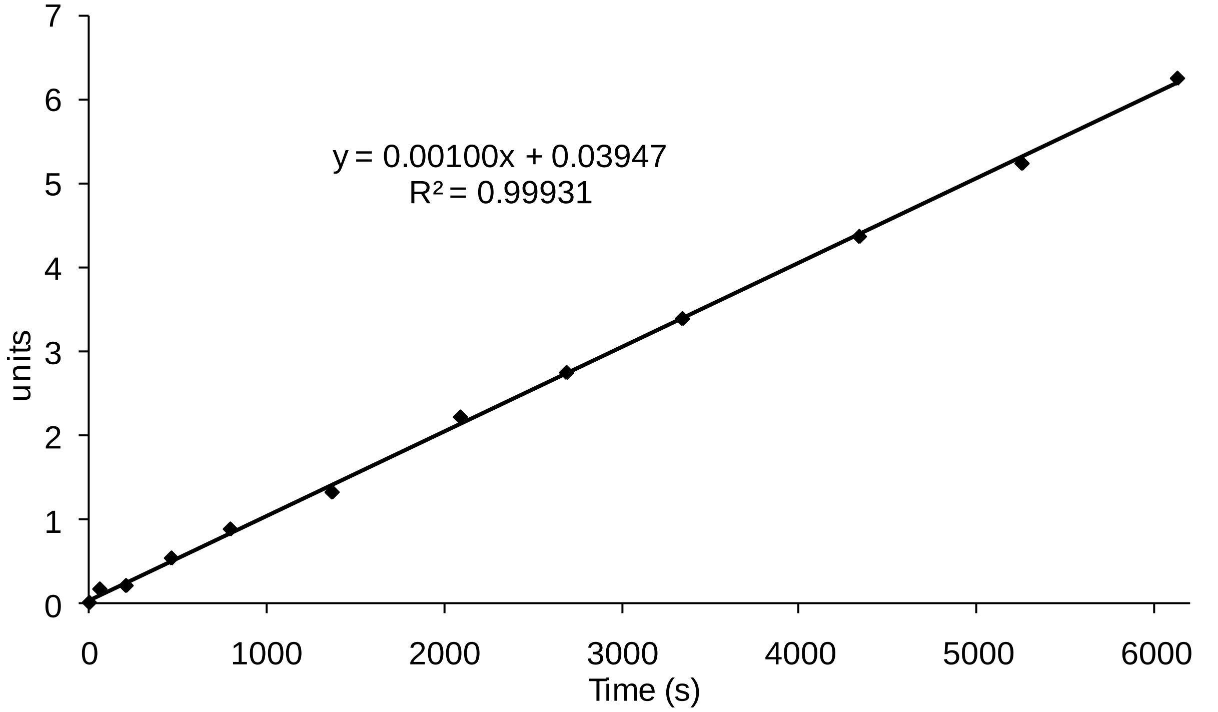

3-Methylbenzaldehyde run 1

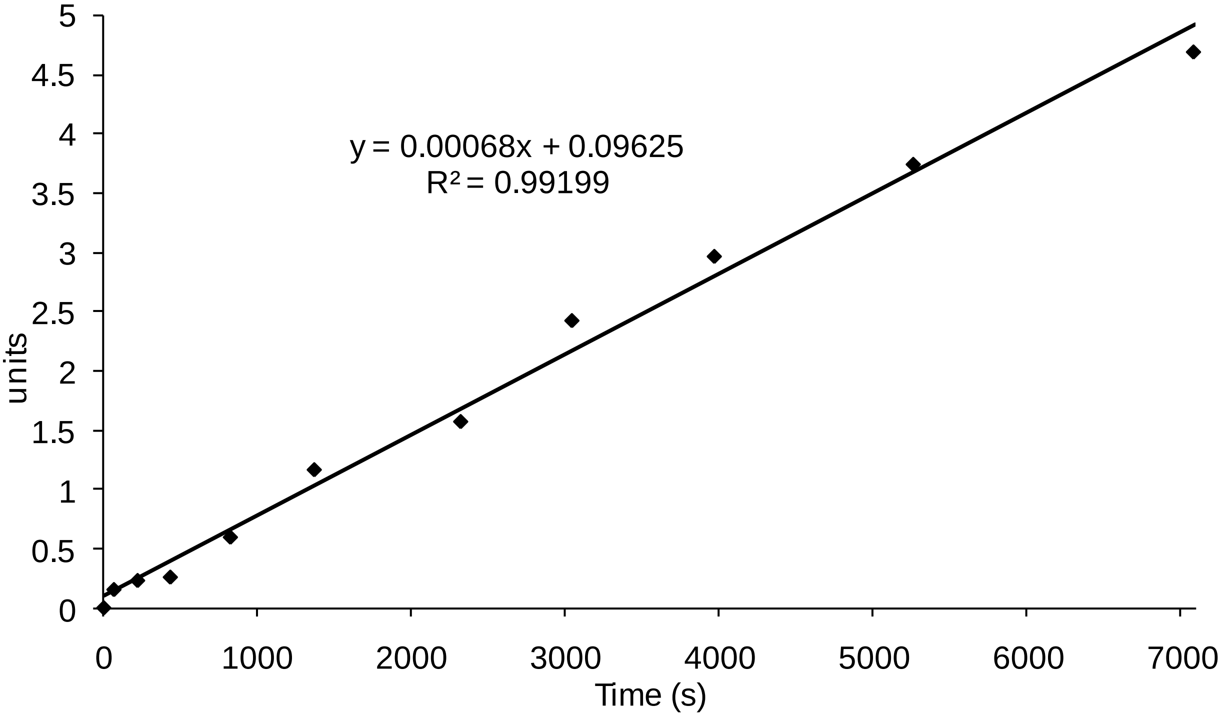

3-Methylbenzaldehyde run 2

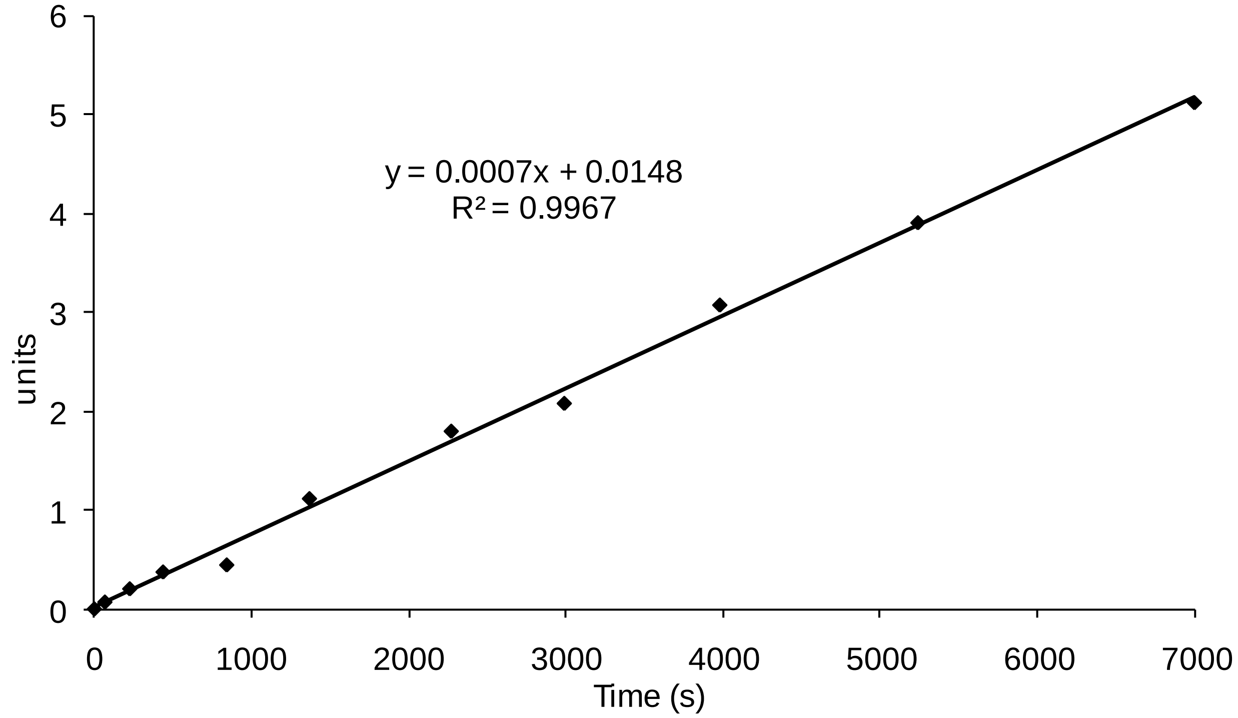

4-Trifluoromethylbenzaldehyde run 1

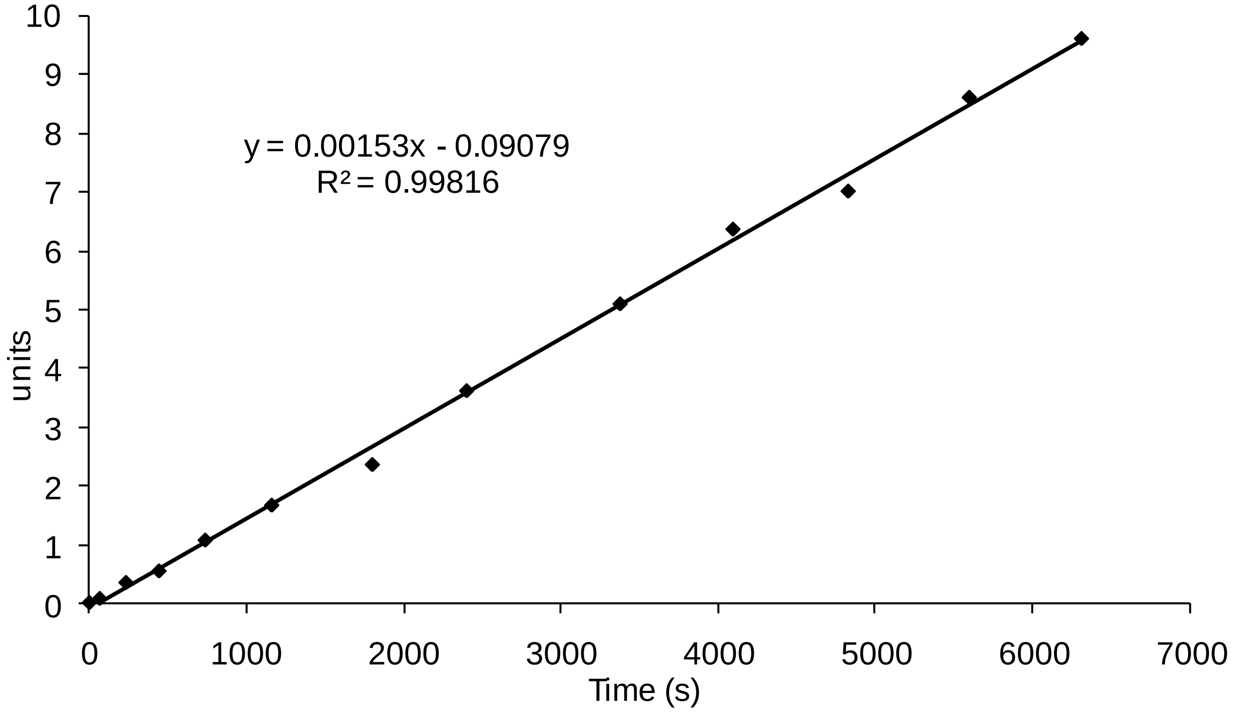

4-Trifluoromethylbenzaldehyde run 2

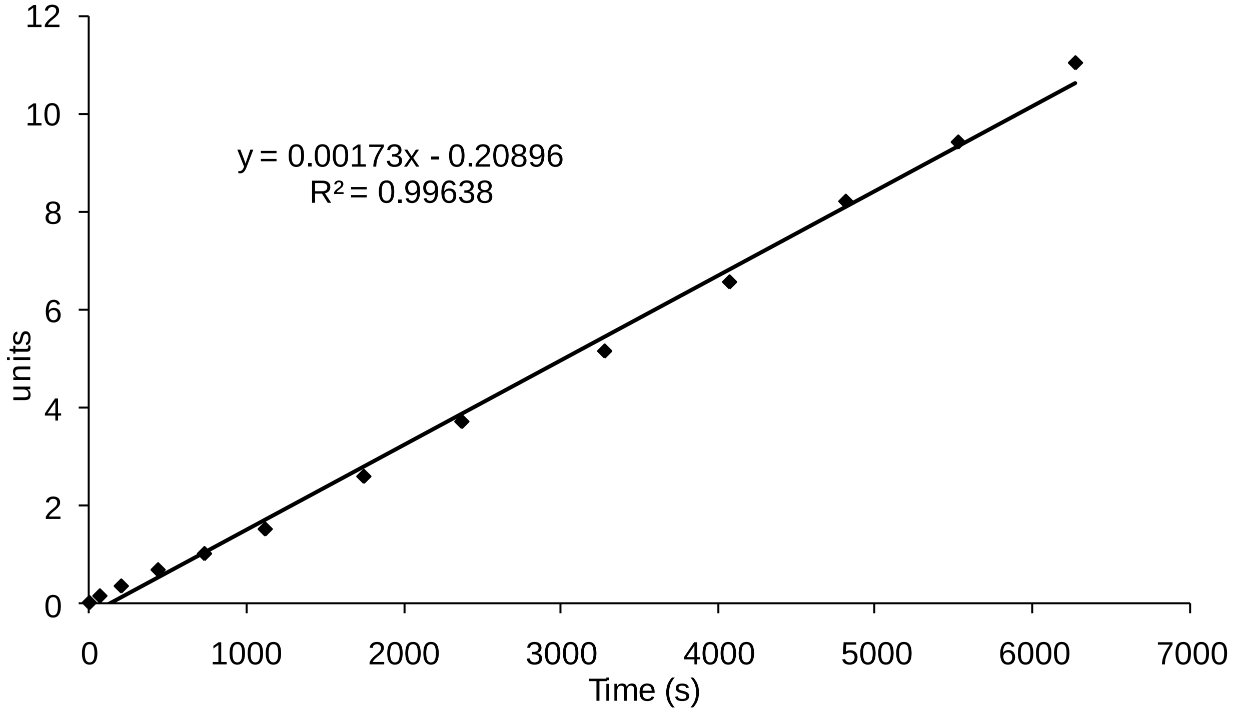

4-Bromobenzaldehyde run 1

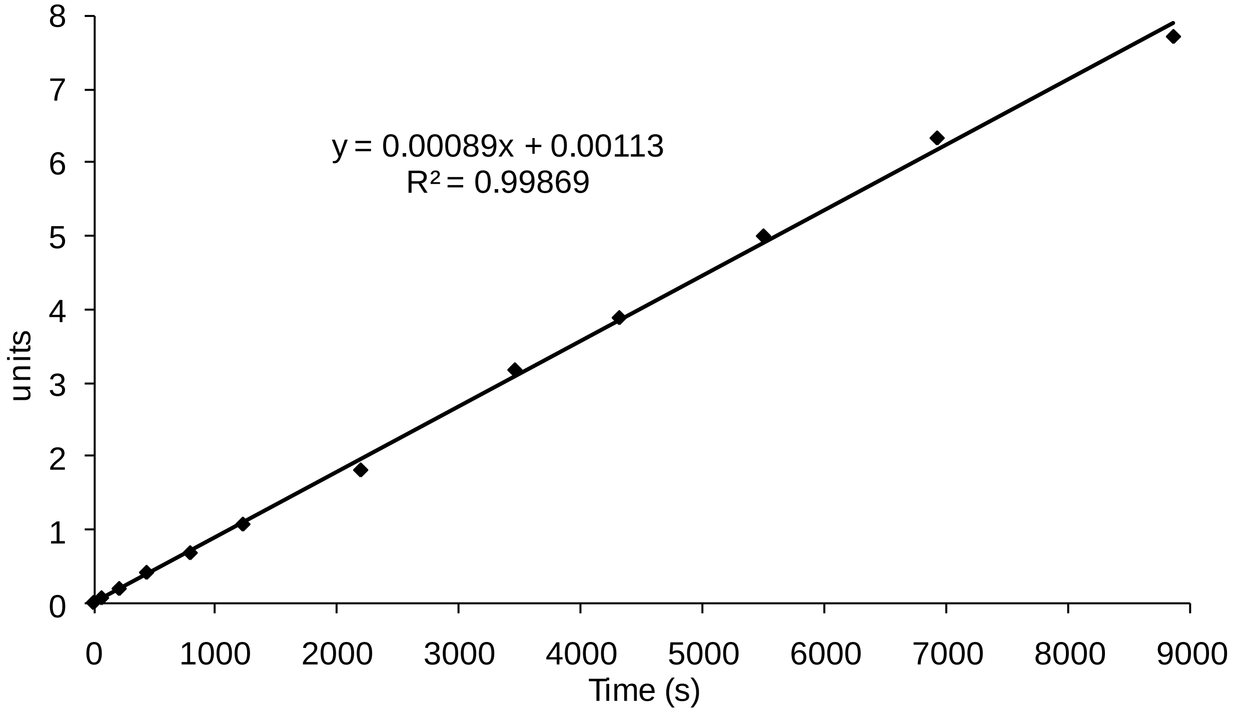

4-Bromobenzaldehyde run 2

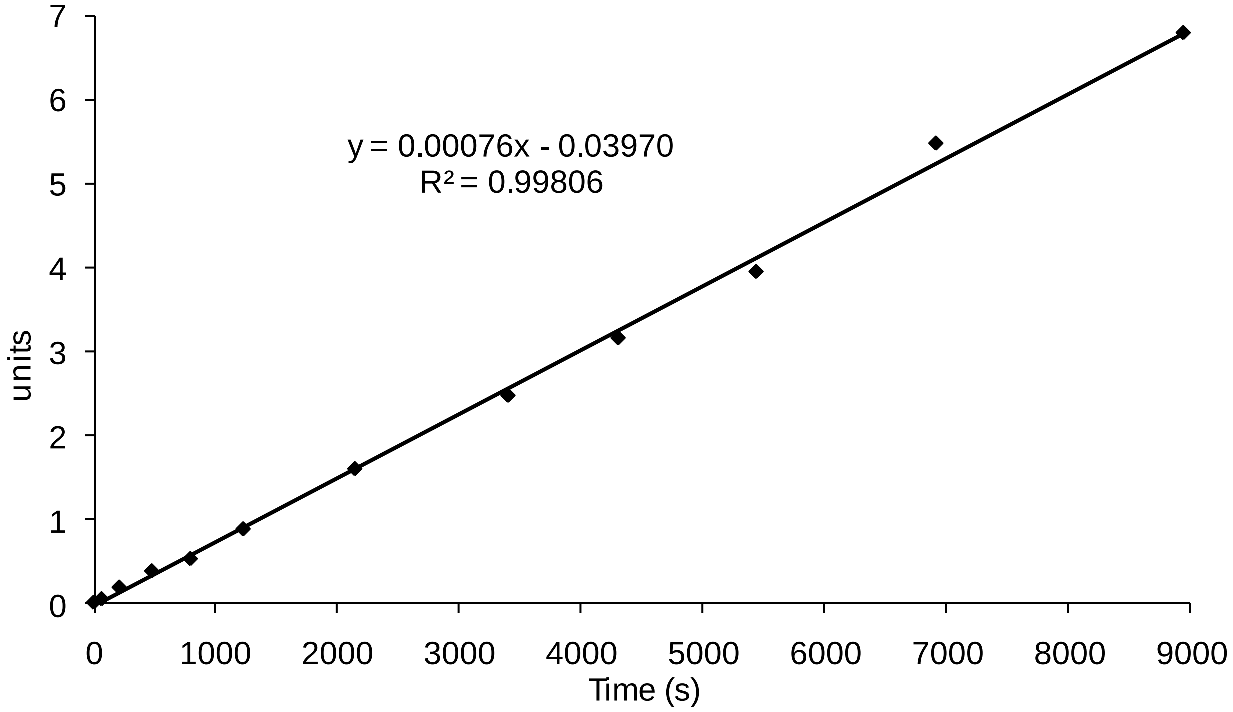

3,5-Difluorobenzaldehyde run 1

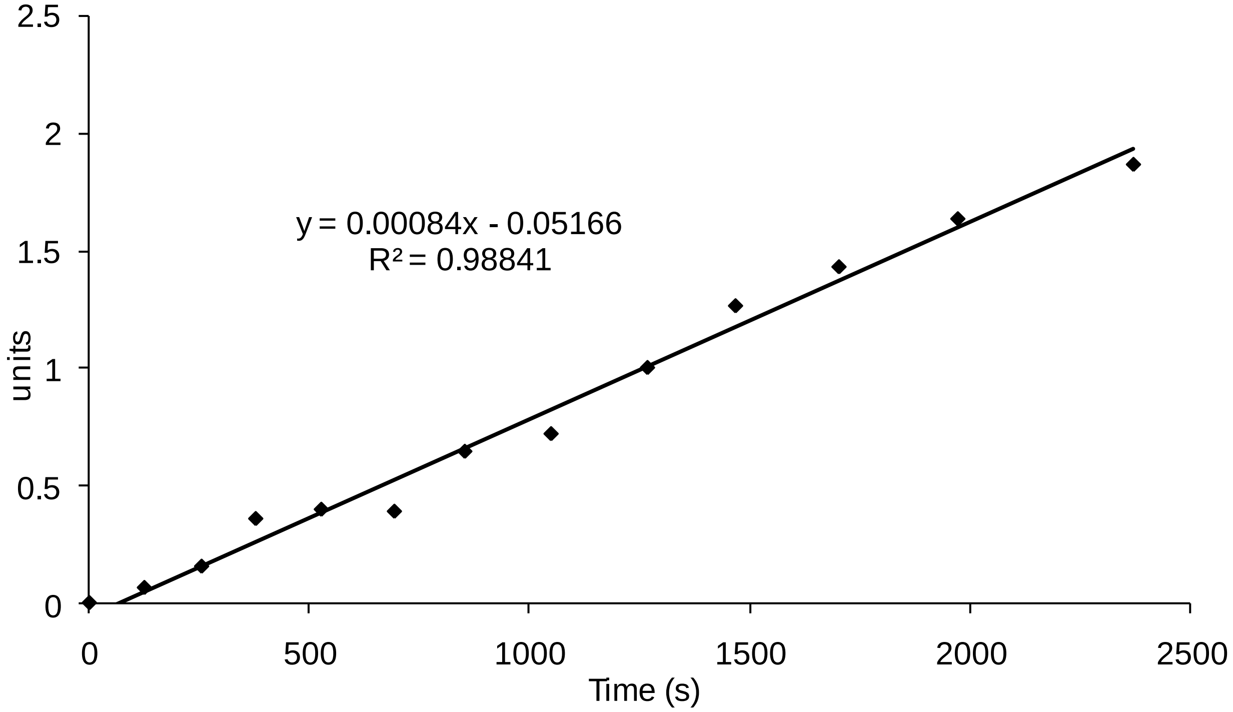

3,5-Difluorobenzaldehyde run 2

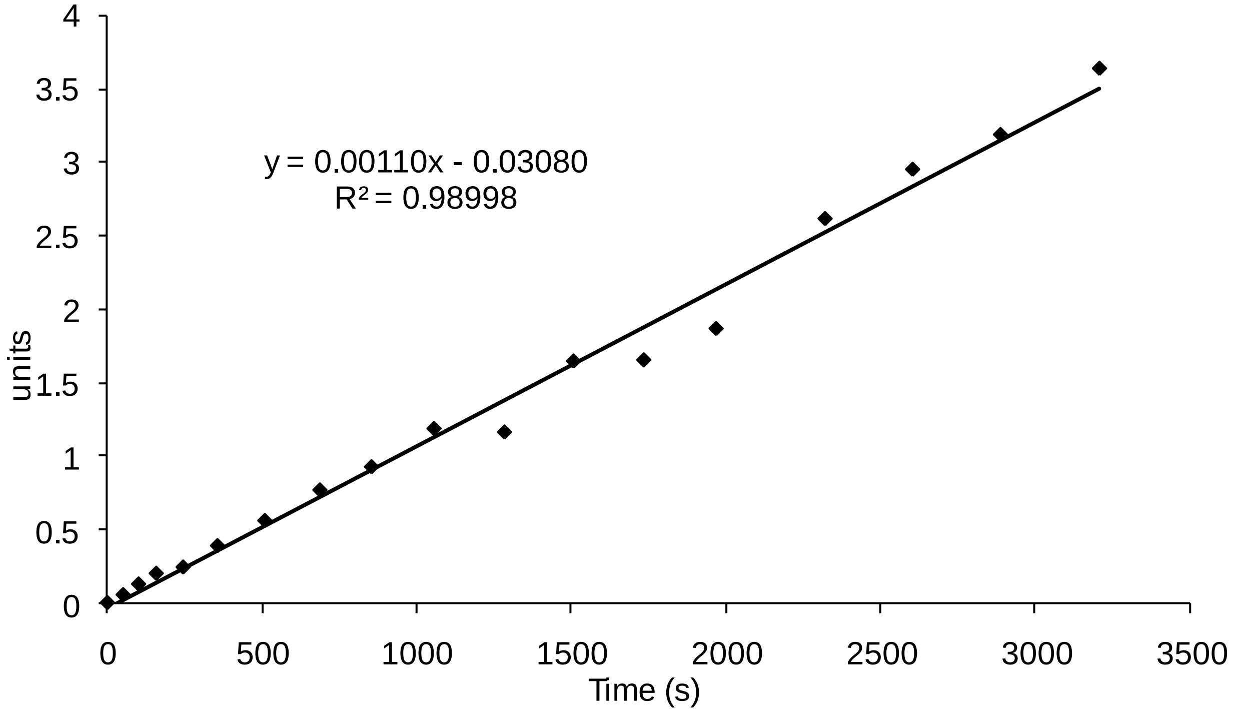

3,4-Dimethylbenzaldehyde run 1

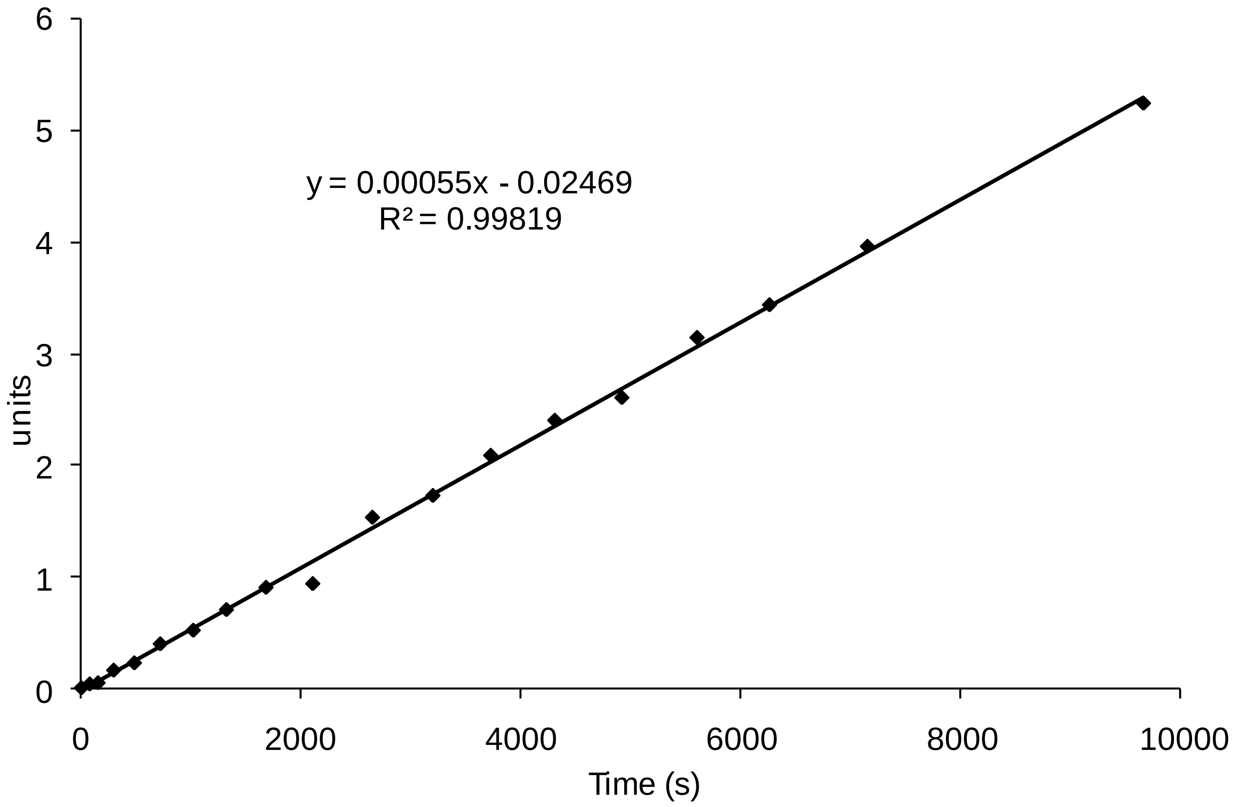

3,4-Dimethylbenzaldehyde run 2

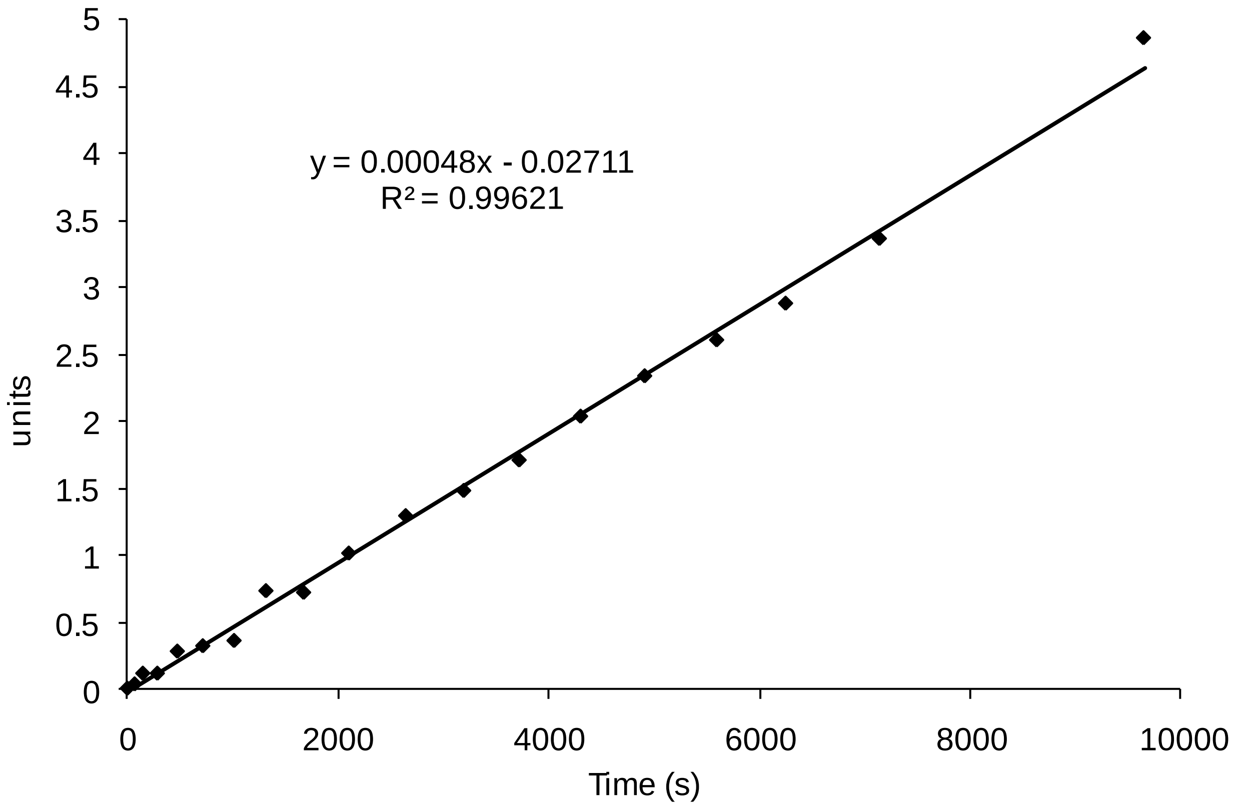

3-Chlorobenzaldehyde run 1

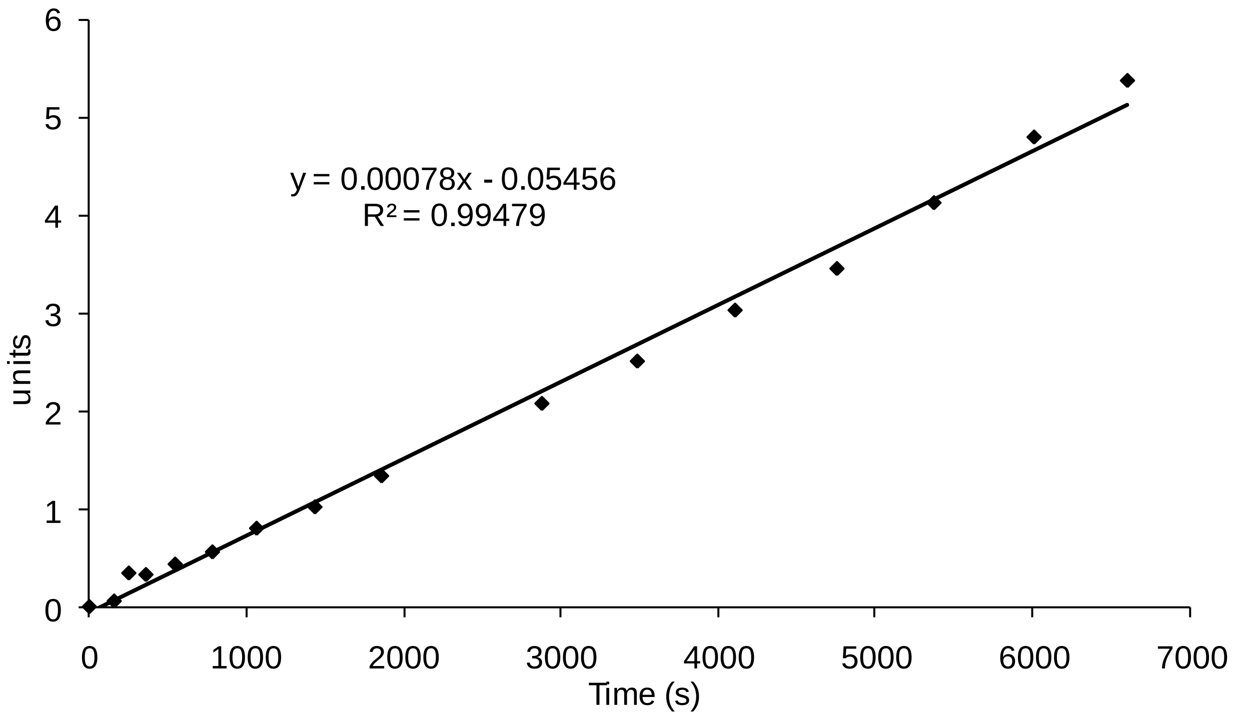

3-Chlorobenzaldehyde run 2

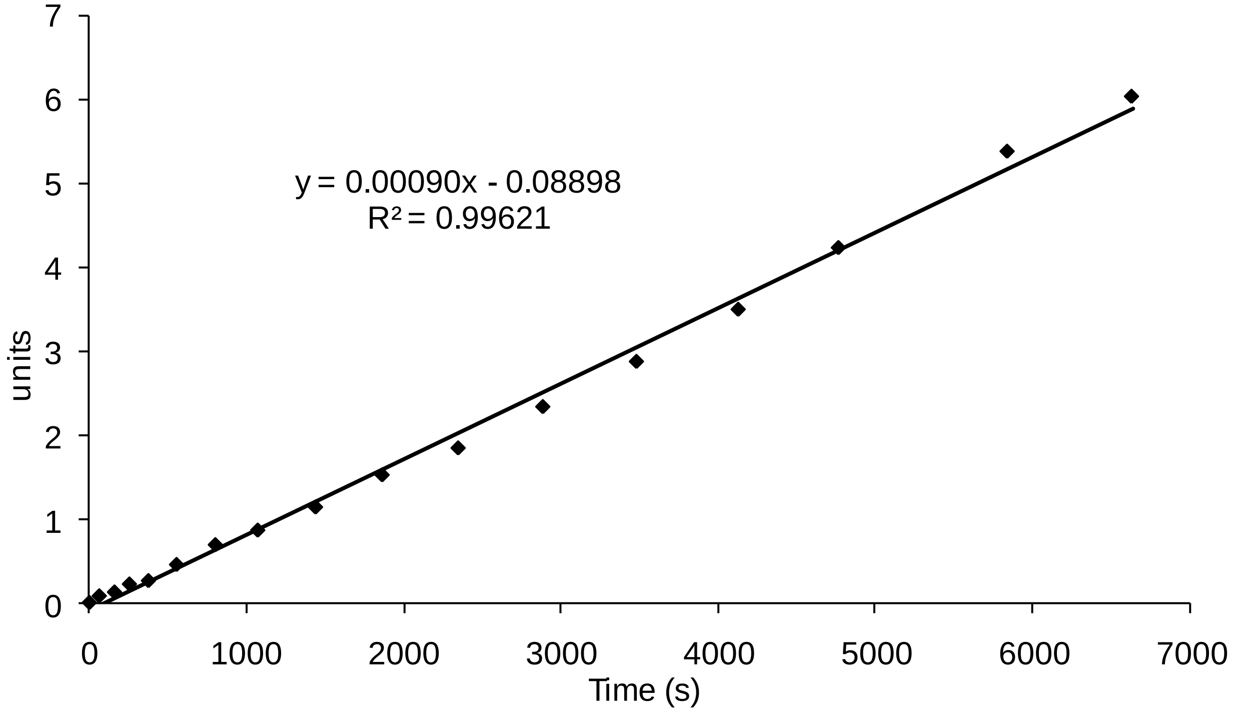

Supplement: File 1 — Analytical data for all chiral compounds. [file Beilstein_J_Org_Chem-06-1043-s001.pdf]
